# Discovery of New Apoptosis-Inducing Agents for Breast Cancer Based on Ethyl 2-Amino-4,5,6,7-Tetra Hydrobenzo[*b*]Thiophene-3-Carboxylate: Synthesis, In Vitro, and In Vivo Activity Evaluation

**DOI:** 10.3390/molecules25112523

**Published:** 2020-05-28

**Authors:** Emad M. Gad, Mohamed S. Nafie, Elsayed H. Eltamany, Magdy S. A. G. Hammad, Assem Barakat, Ahmed T. A. Boraei

**Affiliations:** 1 Chemistry Department, Faculty of Science, Suez Canal University, Ismailia 41522, Egypt; Mohamed_nafie@science.suez.edu.eg (M.S.N.); s.eltamany51@yahoo.com (E.H.E.); magdy_hammad83@yahoo.com (M.S.A.G.H.); 2 Chemistry Department, College of Science, King Saud University, P.O. Box 2455, Riyadh 11451, Saudi Arabia; ambarakat@ksu.edu.sa; 3 Chemistry Department, Faculty of Science, Alexandria University, P.O. Box 426, Ibrahimia, Alexandria 21321, Egypt

**Keywords:** benzo[*b*]thiophene, pyrimidinone, alkylation, MCF-7, HepG-2, JAK2 inhibitor

## Abstract

A multicomponent synthesis was empolyed for the synthesis of ethyl 2-amino-4,5,6,7-tetrahydrobenzo[*b*]thiophene-3-carboxylate **1**. An interesting cyclization was obtained when the amino-ester **1** reacted with ethyl isothiocyanate to give the benzo[4,5]thieno[2,3-*d*][1,3]thiazin-4-one **3**. Acylation of the amino-ester **1** with chloroacetyl chloride in DCM and Et_3_N afforded the acylated ester **4**. The amino-ester **1** was cyclized to benzo[4,5]thieno[2,3-*d*]pyrimidin-4(3*H*)-one **8,** which was reacted with some alkylating agents leading to alkylation at nitrogen **9–13**. Hydrazide **14** was utilized as a synthon for the synthesis of the derivatives **15**–**19**. Chloro-thieno[2,3-*d*]pyrimidine **20** was synthesized and reacted with the hydrazine hydrate to afford the hydrazino derivative **21,** which was used as a scaffold for getting the derivatives **22**–**28**. Nucleophilic substitution reactions were used for getting the compounds **29**–**35** from chloro-thieno[2,3-*d*]pyrimidine **20**. In the way of anticancer therapeutics development, the requisite compounds were assessed for their cytotoxicity in vitro against MCF-7 and HepG-2 cancer cell lines. Twelve compounds showed an interesting antiproliferative potential with IC_50_ from 23.2 to 95.9 µM. The flow cytometric analysis results showed that hit **4** induces the apoptosis in MCF-7 cells with a significant 26.86% reduction in cell viability. The in vivo study revealed a significant decrease in the solid tumor mass (26.6%) upon treatment with compound **4**. Moreover, in silico study as an agonist for inhibitors of JAK2 and prediction study determined their binding energies and predicted their physicochemical properties and drug-likeness scores.

## 1. Introduction

The development and discovery of high efficacy anticancer agents by many molecular oriented strategies and techniques have increased in the last decades [1]. Globcan in 2018 provided a statistical report worldwide on the global burden of cancer, which shows 18.1 million new cancer incidences and 9.6 million mortalities. The female breast cancer is the second type of cancer, leading to death with 11.6%. The statics show that in every 8–10 women, one gets developed with breast cancer. Moreover, the fourth fatal cancer mortality is liver cancer with 8.2% [2].

There are several reasons for the tremendous growth of cancer mortality and new cases worldwide due to an increasing number of the population, high rate of aging, as well as prevalence changes and distribution of the risk factor for cancer linked with socioeconomic development. More specifically, the risk factors responsible for the development of breast cancer are reproductive factors (e.g., late menopause, multiparty, premature menarche, do not breastfeed), genetic factors (if her mother has breast cancer), lifestyle and dietary related factors (e.g., obesity, drinking, and smoking), and environmental factors (exogenous estrogen exposure for a long time). There are several types of breast cancer treatments including chemotherapy, radiotherapy, surgery, immune, and hormone therapy but still these treatments have diverse side effects. The urgent need to discover and develop a high efficacy anticancer therapy is highly challenged. Several therapeutic potentials have been discovered for cancer treatment with different targets; one of the most promising targets for cancer therapies is the JAK family [3]. JAK family members (JAK1, JAK2, JAK3, and TYK2) play an important role in the pathogenesis of many immunological disorders and cellular malignancies. The activity of JAK kinase was described in a series of abnormal cell proliferation of the hematologic neoplasias, including B-cell non-Hodgkin’s and Hodgkin’s lymphomas, myeloid leukemias, and lymphoid [4]. One of the most molecular targets and techniques for antiproliferative and pro-apoptotic effects are to design potential JAK inhibitors.

**Figure 1:**
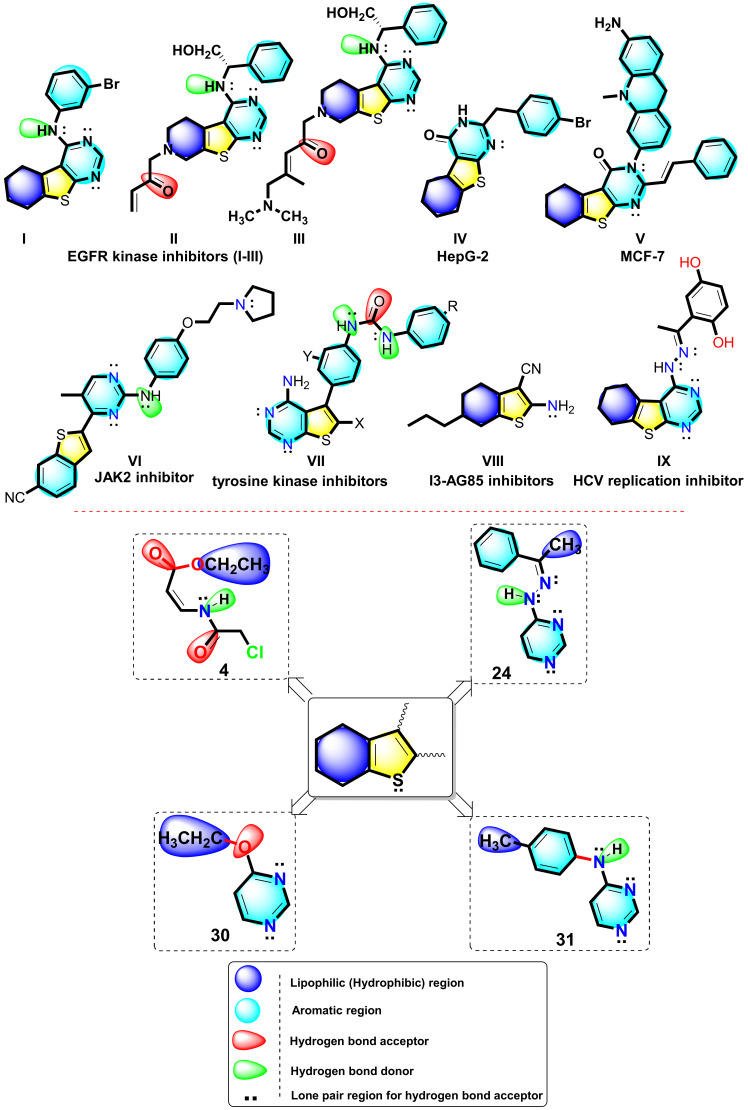
Biologically active compounds based on benzo[*b*]-thiophene and cycloalkyl[*b*]thiophene and pharmacophoric/functional groups relationship.

Fused heterocycles, based on pyrimidine scaffolds, have gained significant attention due to a wide range of pharmacological applications [5]. Especially, thienopyrimidines have been discovered as anticancer agents [6,7], EGFR kinase inhibitors (compound **I,** IC_50_ = 2.6 [8]; compound **II**, IC_50_ = 0.008, and compound **III**, IC_50_ = 0.007 µM, respectively) [9], compound **IV** selective against HepG-2 (IC_50_ = 14.9 µM) [10], compound **V** selective against the MCF-7 breast cancer line (IC_50_ = 0.10 µM) [11], and tyrosine kinase inhibitors (compound **VI**) [12]. TargeGen, Inc. et al. have been designed as an JAK2 inhibitor based on the core structure of anilinopyrimidine, and the cell proliferation assay has shown the EC_50_ = 3 nM in a JAK2 V617F (compound **VI**) [13]. The benzo[*b*]-thiophene scaffold plays a crucial rule for many STAT3 inhibitors [14,15]. Additionally, the substituted cycloalkyl[*b*]thiophene core structure possessed a cytotoxic activity [16], also exhibited biological activities such as the AChE inhibitor [17], I3-AG85 inhibitor (compound **VIII**) [18], as well as the HCV replication inhibitor (compound **IX**, Figure 1) [19], antiviral [20], anti-inflammatory [21,22], antibacterial [23,24], and antitumor activities [25,26,27,28,29]. In the proceeding text, we have synthesized a set of compounds based on the cycloalkyl[*b*]thiophene with different hybrids tethered pharmacophoric/functional groups diversity (Figure 1). The target hybrids were examined against two cancer cell lines (breast cancer MCF-7 and liver cancer HepG-2), followed by an in vivo study along with the flow cytometric analysis for the most active member were also carried out. Finally, an in silico molecular docking study such as the JAK2 inhibitor determined their binding energies and predicted their physicochemical properties and drug-likeness scores.

## 2. Results and Discussion

### 2.1. Chemistry

#### 2.1.1. Synthesis

**Scheme 1:**
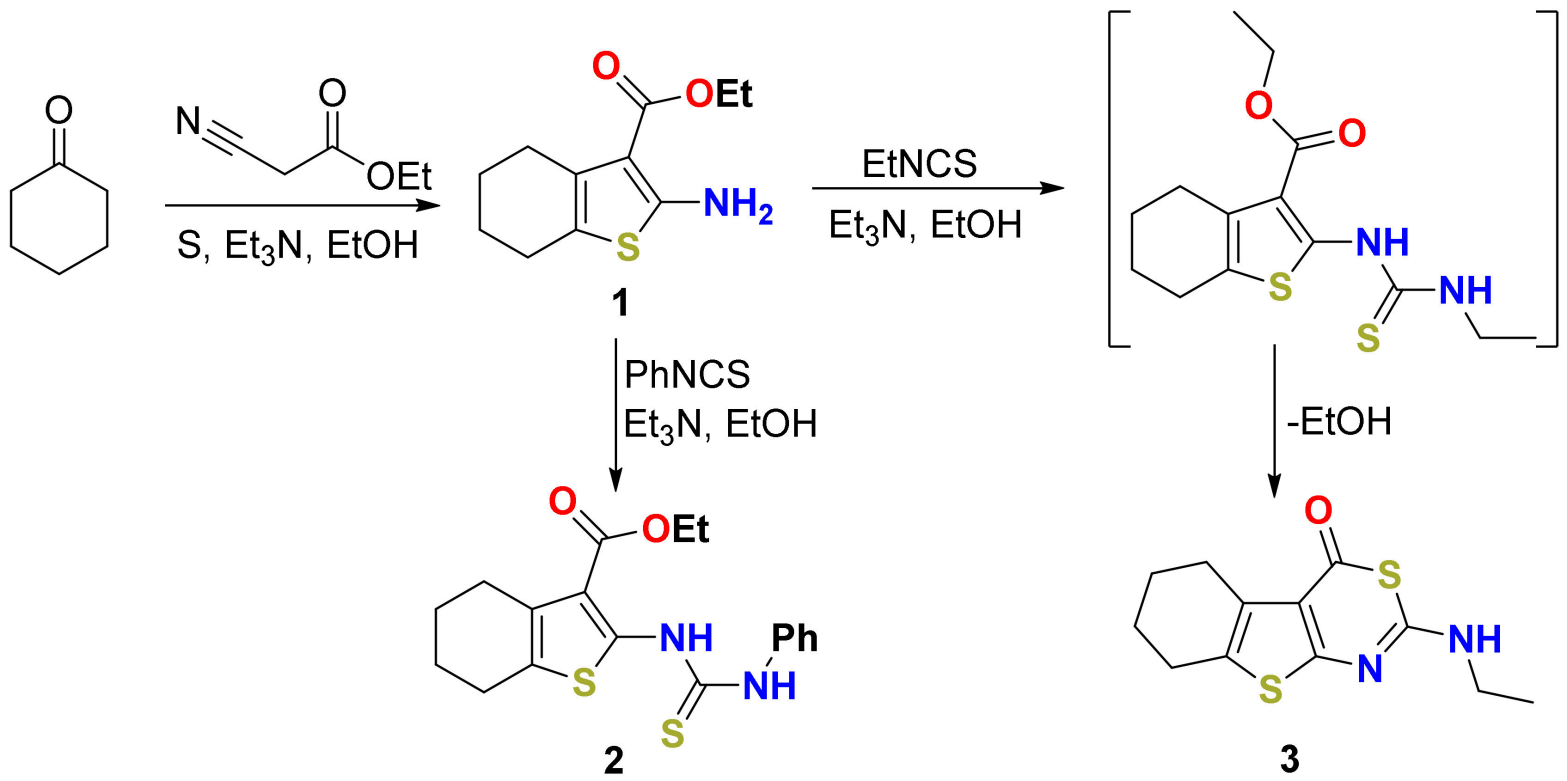
Multicomponent synthesis of **1** and its reaction with isothiocyanates.

The benzo[*b*]thiophene analogue **1** was synthesized in high yield using the multicomponent strategy from ethyl cyanoacetate, cyclohexanone, sulfur and triethylamine in ethanol. The amino-ester **1** was reacted with phenyl isothiocyanate in ethanol containing Et_3_N to afford the *N,N*-disubstituted thiourea derivative **2**. Whereas, when it was reacted with ethyl isothiocyanate under the same conditions, surprisingly it afforded the 2-(ethylamino)-5,6,7,8-tetrahydro-4*H*-benzo[4,5]thieno[2,3-*d*][1,3]thiazin-4-one **3** (Scheme 1).

**Scheme 2:**
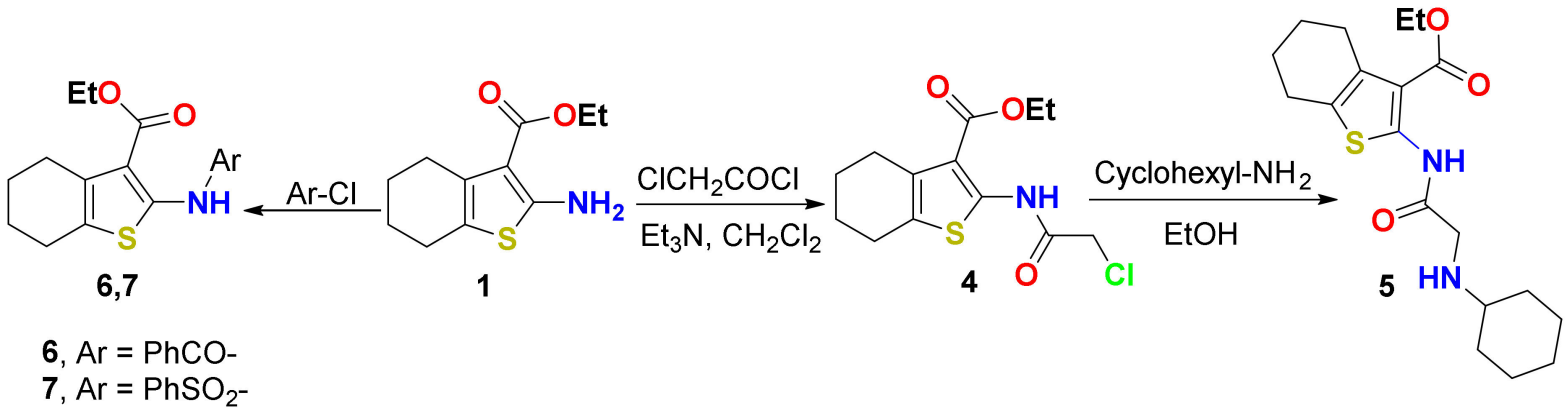
Synthesis of **4**–**7** starting from compound **1.**

Acylation of the amino-ester **1** with chloroacetyl chloride in DCM and Et_3_N afforded the acylated ester **4**. Compound **4** was used for the alkylation of cyclohexylamine to afford the alkylated product **5**. The benzoylation of the benzo[*b*]thiophene analogue **1** was done by a reaction with benzoyl chloride in benzene containing triethylamine to afford the amide **6**. The reaction of **1** with benzene sulfonyl chloride in ethanol yielded the sulfonamide **7** (Scheme 2).

**Scheme 3:**
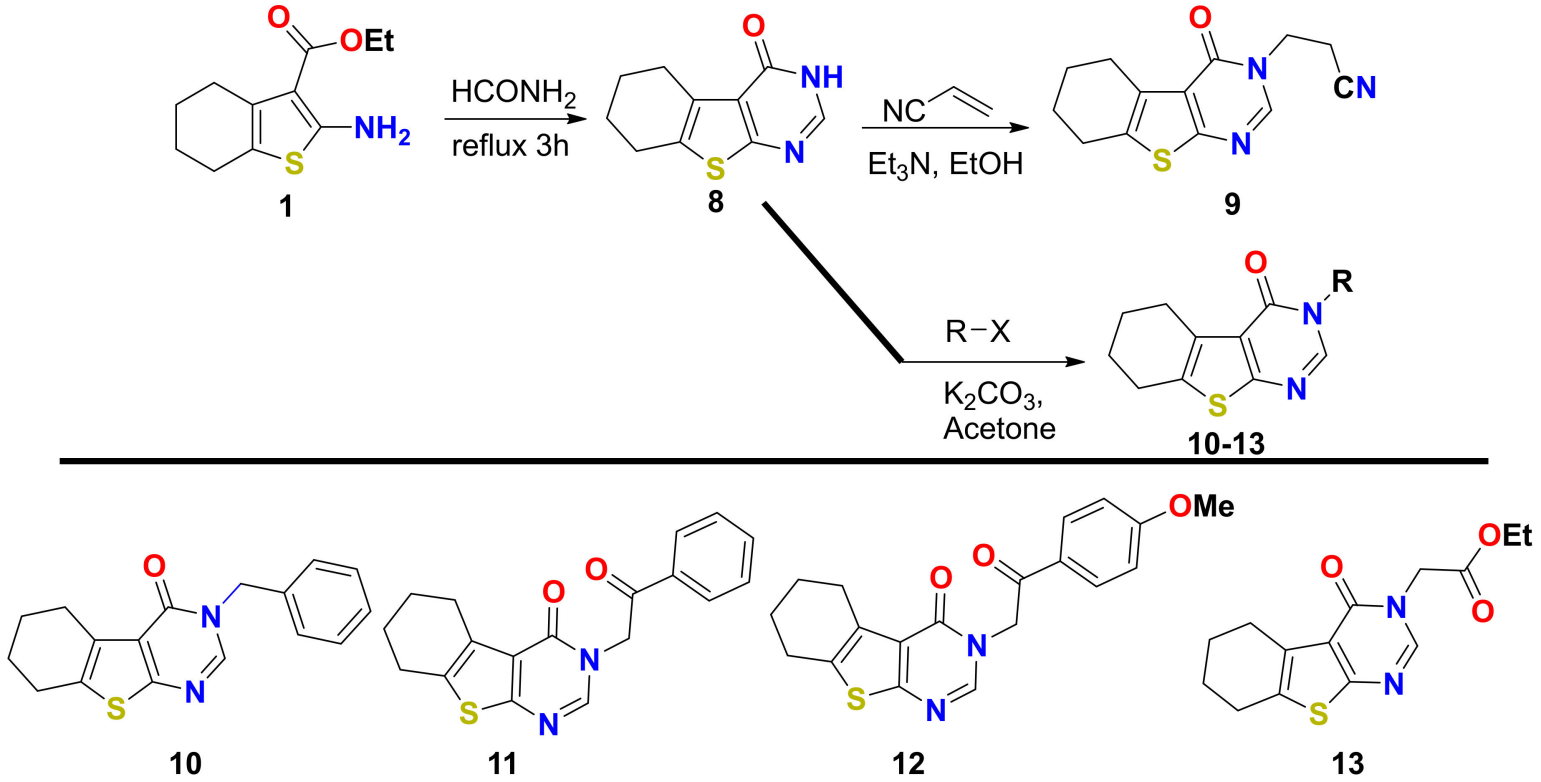
Alkylation of pyrimidinone **8** with different alkylating agents.

Cyclization of the amino-ester **1** was done by reflux with formamide for 3 h to give tetrahydrobenzo[4,5]thieno[2,3-*d*]pyrimidin-4(3*H*)-one **8**. Michael additions of pyrimidinone **8** to acrylonitrile as the Michael acceptor in ethanol containing Et_3_N led to an addition at nitrogen to give the Michael adduct **9**. Alkylation of **8** with benzyl bromide, phenacyl bromide, 4-methoxyphenacyl bromide, and ethyl chloroacetate in DMF and K_2_CO_3_, afforded the *N*-alkylated products **10**–**13,** respectively (Scheme 3).

**Scheme 4:**
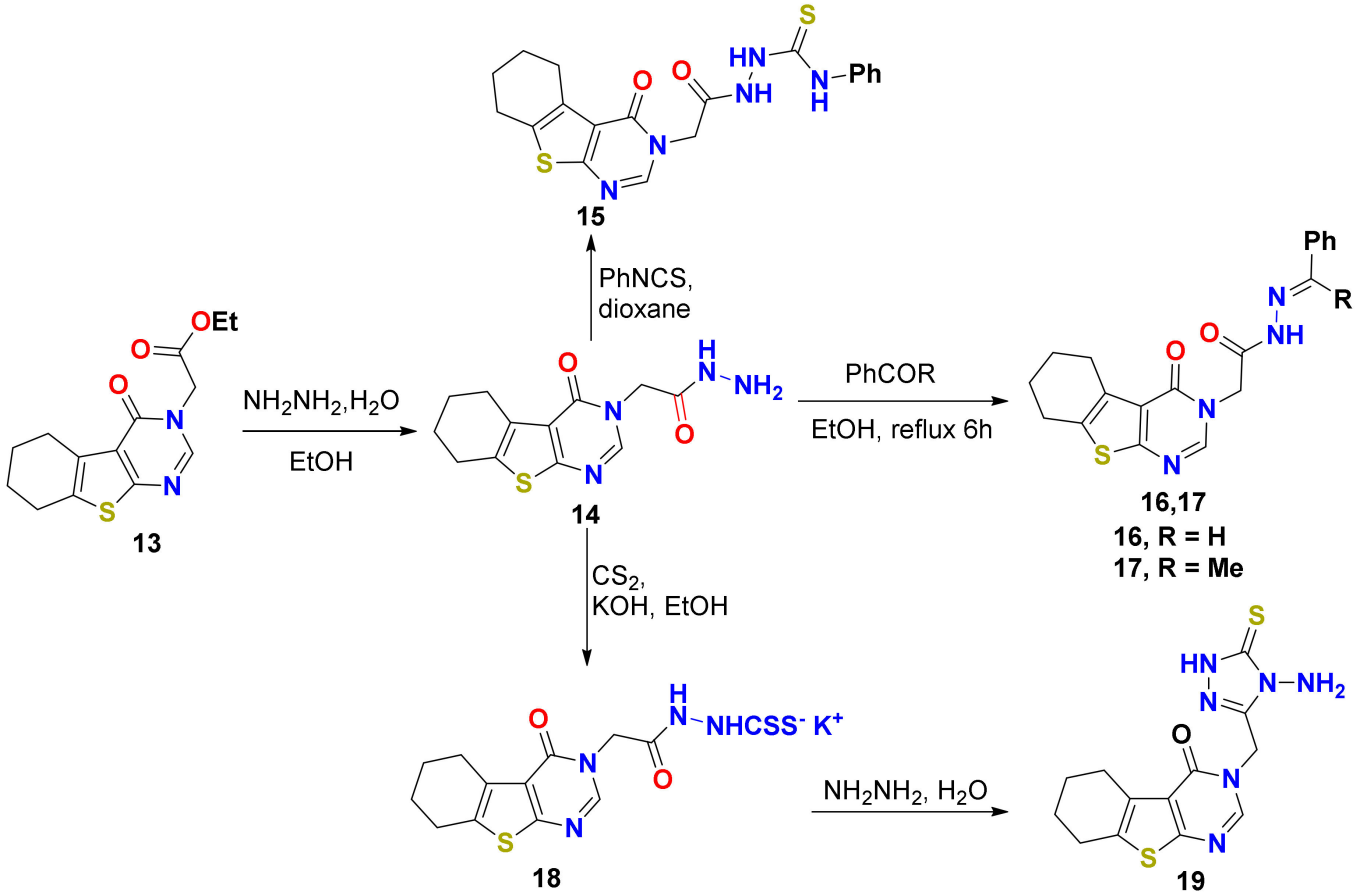
Formation of hydrazones, thiosemicarbazide, and linking new 1,2,4-triazole nucleus to the system via methylene spacer **19**.

Hydrazinolysis of the ester **13** to hydrazide **14** was done by a reaction with hydrazine hydrate in ethanol. The heating hydrazide **14** with phenyl isothiocyanate in dioxane for 6 h gives the phenyl thiosemicarbazide product **15**. The hydrazide **14** was refluxed with benzaldehyde and acetophenone in ethanol to give the hydrazones **16** and **17,** respectively. Linking the 4-amino-1,2,4-triazolethione moiety to the benzo[4,5]thieno[2,3-*d*]pyrimidin-4(3*H*)-one system separated by a methylene spacer was achieved by a reaction with CS_2_/aq. KOH/EtOH, to give potassium dithiocarbazate **18,** which was cyclized by a reaction with hydrazine hydrate and acidification to give **19** (Scheme 4).

**Scheme 5:**
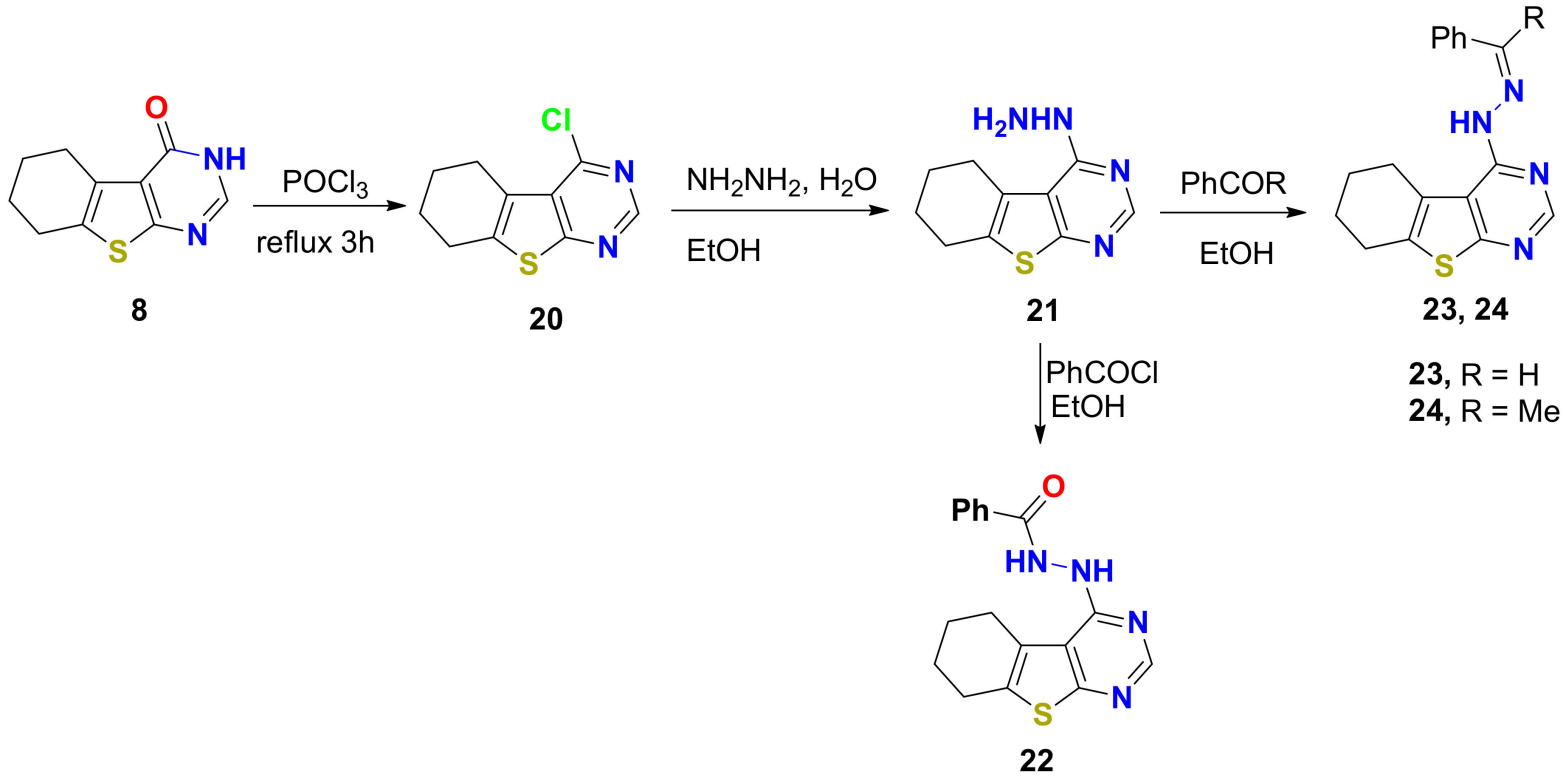
Synthesis and condensation of the hydrazino derivative **21.**

The chlorination of **8** was achieved by reflux in POCl_3_ to yield the chloro derivative **20**. A reaction of **20** with NH_2_NH_2_ in ethanol formed the hydrazino compound **21**. The benzoylation of **21** with benzoyl chloride provided the *N*-benzoyl derivative **22**, while the condensation of **21** with benzaldehyde and acetophenone afforded compounds **23** and **24**, respectively (Scheme 5).

**Scheme 6:**
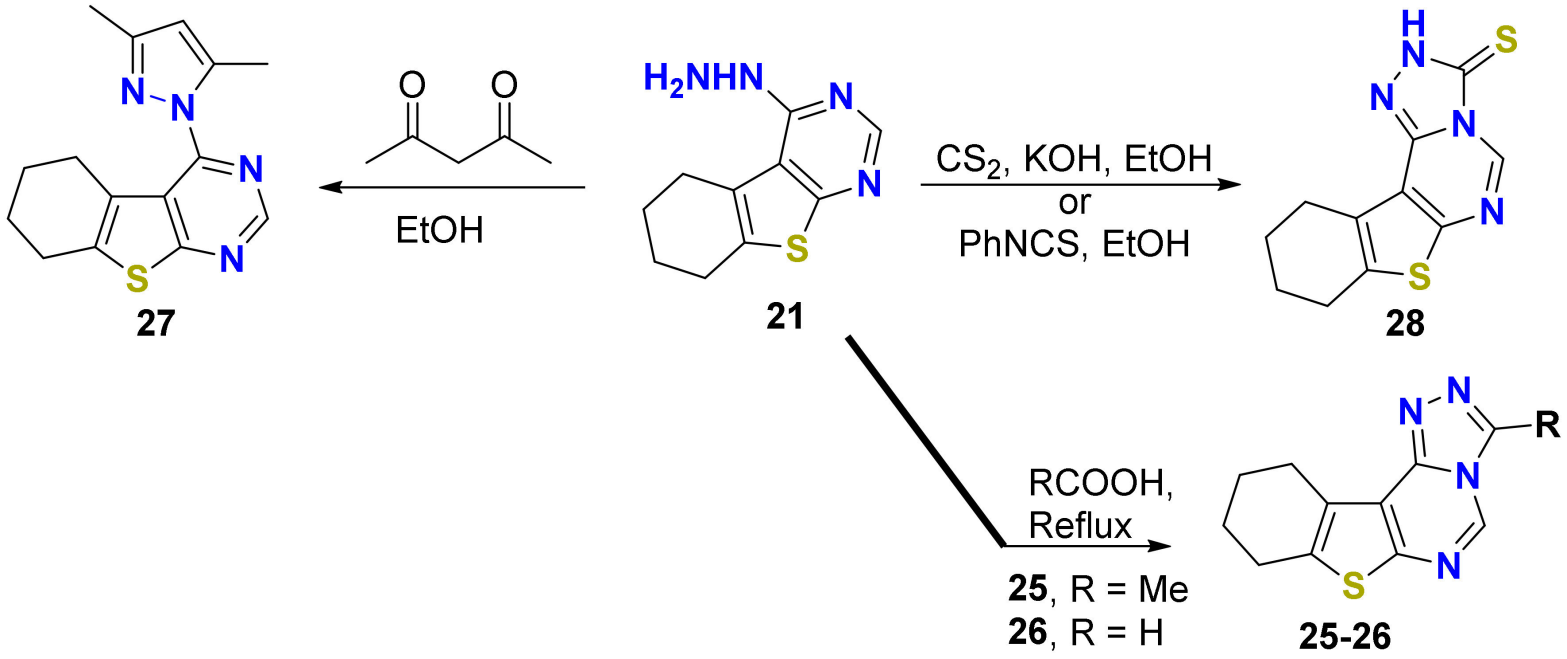
Cyclization of the hydrazino derivative **21**.

Cyclization of the hydrazino derivative **21** to triazolo-pyrimidines **25** and **26** was done by reflux in acetic and formic acids, respectively. The condensation of **21** with acetyl acetone in ethanol yielded the pyrazolo derivative **27**. A reaction of **21** with carbon disulfide or phenyl isothiocyanate led to cyclization to the triazolo-pyrimidine-thione **28** (Scheme 6).

**Scheme 7:**
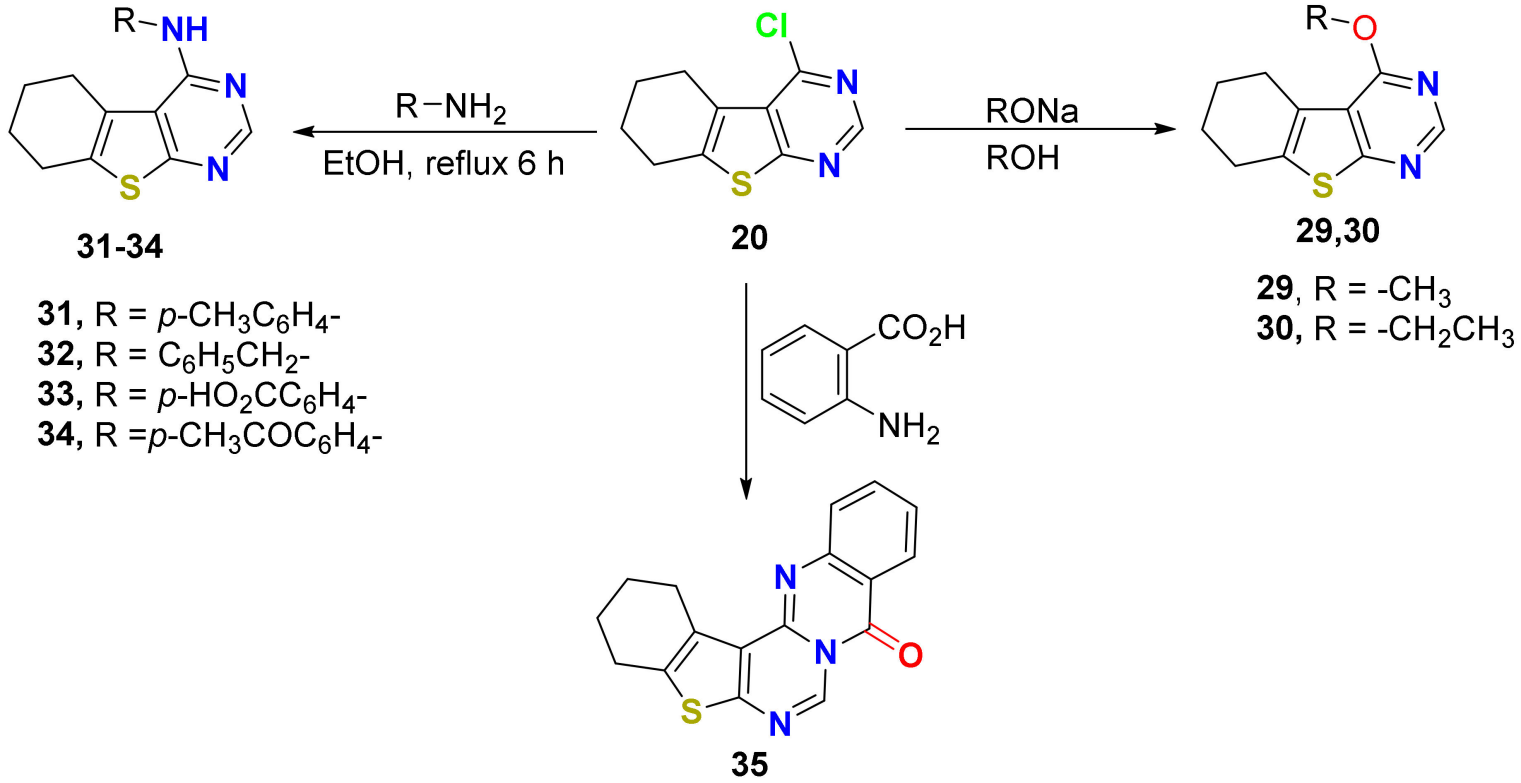
Synthesis of compounds **29**–**35** from the chloro derivative **20**.

Oxygen nucleophiles such as sodium methoxide and sodium ethoxide were used to substitute the chloride of **20** to afford the ethers **29** and **30**, respectively. A reaction of the chloro derivative **20** with nitrogen nucleophiles such as *p*-toluidine, benzyl amine, 4-aminobenzoic acid, and 4-acetylaniline afforded the substituted products **31**–**34**, respectively. The reaction of the chloro derivative **20** with 2-aminobenzoic acid afforded the pyrimido-quinazolinone **35** (Scheme 7).

#### 2.1.2. Structural Characterization

The ^1^H NMR of **1** displayed the amino group as an exchangeable signal at *δ* 7.15 ppm. The ethoxy group protons (CH_3_CH_2_O) appeared at *δ* 4.15 and 1.25 ppm. ^13^C NMR showed the ester group carbons at *δ* 165.53 (C=O), 59.03 (OCH_2_), and 14.79 ppm for CH_3_. The structure of **2** showed the two exchangeable signals at *δ* 11.78 and 10.88 ppm. The thiocarbonyl and carbonyl groups were detected at *δ* 176.36 and 166.02 ppm, respectively. Compound **3** missed the ester group signals and showed only the NH signal at *δ* 13.43 ppm in addition to the ethyl group signals at 4.40 and 1.20 ppm. The ethyl group carbon signals were found at *δ* 41.08 and 12.26 ppm, which strongly recommend the cyclic structure of **3**. The acetamido group signals in **4** were found at 11.66 ppm for NH and 4.57 ppm for CH_2_Cl. The two carbonyl groups were identified at *δ* 165.45 and 164.23 ppm. The benzamide **6** and sulfonamide **7** NMR showed the NH signals at *δ* 12.03 and 10.36 ppm, respectively. Due to cyclization, the structure of **8** showed the NH as an exchangeable signal at 12.20 ppm and pyrimidine CH at *δ* 7.96 ppm. The carbonyl group was identified at *δ* 162.89 ppm. The *aza*-Michael addition and nitrogen alkylation of **8** to produce **9**–**13** were deduced from the missing NH signal and illustrated the methylene carbon signals directly attached to the ring nitrogen (NCH_2_) at *δ* 41.84, 48.83, 52.05, 51.64, and 47.25 ppm, respectively. The NMR of hydride **14** showed the -CO-NHNH_2_ group as two exchangeable signals at *δ* 9.36 and 4.25 ppm, whereas the carbonyl carbon was identified at *δ* 166.55 ppm. The thiosemicarbazide **15** displayed three NH signals at *δ* 10.56, 9.77, and 9.33 ppm. The thiocarbonyl and carbonyl carbons were detected at *δ* 181.06, 167.10, and 162.50 ppm. Hydrazones **16** and **17** were confirmed from the disappearance of the NH_2_ signal and the detection of benzylidene CH signals around 8.30 ppm. Compound **19** displayed the NH of the ring at *δ* 13.54 ppm and the NH_2_ protons at 5.61 ppm. The C=S was found at *δ* 167.32 ppm. The structure of **20** missed the NH signal, while **21** illustrated the NHNH_2_ exchangeable signals at *δ* 7.81 and 4.55 ppm. The benzoylated product **22** showed two NH signals at *δ* 9.64 and 8.29 ppm. The hydrazones **23** and **24** displayed the NH signal at *δ* 11.79 and 11.49 ppm, respectively, while the benzylidene CH was observed at 7.80 ppm in the case of **23**. The cyclic structures **25** and **26** did not show any NH signal; only CH signals appeared in the aromatic region at 9.48 ppm in the case of **25** and two of the CH signals at 9.55 and 8.60 ppm in the case of **26**. The pyrazole ring structure **27** revealed two CH protons at *δ* 8.97 and 6.17 ppm for the pyrimidine and pyrazole CH. The triazole-thione moiety construction in the case of **28** was identified from the detection of an NH at *δ* 14.62 ppm and the thiocarbonyl carbon at 160.56 ppm. The ethers **29** and **30** showed the CH signal of the pyrimidine ring at *δ* 8.55 and 8.52 ppm in addition to the alkoxy group indicating signals. The NH signals appeared in the spectra of **31**–**34** at *δ* 8.34, 8.13, 5.09, and 8.49 ppm, respectively. The five-ring fused system **35** displayed the pyrimidine CH at *δ* 9.34 ppm, and the four phenyl protons appeared between *δ* 8.35 and 7.57 ppm.

### 2.2. Biological Activity

#### 2.2.1. Antitumor Activity Evaluation

The synthesized compounds were tested for their antiproliferative activity against MCF-7 and HepG-2 cancer cell lines. Twelve compounds displayed a significant activity, which ranged from 23.2 to 95.9 μM. Compounds **4**, **24**, **29**, **30**, and **31** were the most active with an IC_50_ range from 23.2 to 49.9 μM. Compounds **5**, **15**, **21**, **26**, **27**, **28**, and **33** revealed a moderate activity in the range from 52.9 to 95.9 μM. The remaining derivatives showed a lower activity (Table 1).

#### 2.2.2. Flow Cytometric Analysis

To demonstrate the apoptotic mechanistic mode of action, the MCF-7 cell line was treated with compound **4** (IC_50_ = 23.2 µM, 48 h incubation) compared to untreated cells as the control, which include flow cytometric analyses including an FITC/Annexin-V-FITC/PI differential apoptosis/necrosis assessment, DNA content-flow cytometry aided cell cycle analysis, and acridine orange quantitative autophagy assessment. All flow cytometric methodologies were performed according to the standard protocols, as previously described by Kattan et al., 2020 [30].

##### FITC/Annexin-V-FITC/PI Differential Apoptosis/Necrosis Assessment

**Figure 2:**
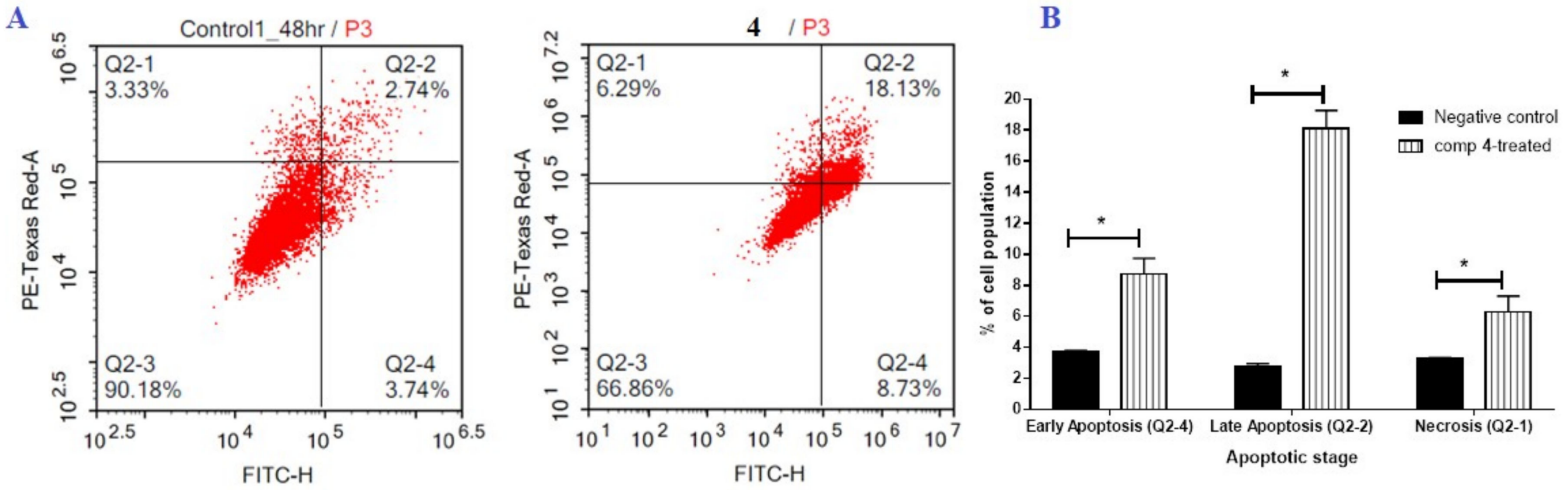
Induction of apoptosis in MCF-7 cells by compound **4** (23.2 μM, 48 h). Cytograms showing annexin-V/propidium iodide stained MCF-7 cells: (**A**) MCF-7 cells nontreated with the negative control and treated with compound **4**; (**B**) bar chart representation of the apoptosis analysis results, quadrant charts show Q2-1 (necrotic cells, AV–/PI+), Q2-2 (late apoptotic cells, AV+/PI+), Q2-3 (normal cells, AV–/PI–), Q2-4 (early apoptotic cells, AV+/PI–). * *p* < 0.05 unpaired *t*-test compared to negative control.

Double-staining with annexin-FITC and propidium iodide (PI) on MCF-7 cells treated with compound **4** (IC_50_ = 23.2 µM), and nontreated cells served as the negative control for 48 h to investigate whether it has an induction of apoptosis and/or cell cycle progression. As displayed in Figure 2, results show that the treatment caused apoptosis in MCF-7 cells with a substantial cell viability reduction of 26.86%. The percentage of cells sub-populating in early apoptosis (AV+/PI−) was 8.73, which is 2.3 times more relative to the untreated regulation. In addition, the average late apoptotic sub-population (AV+/PI+) count was 18.13%, which is 6.6 times higher than the untreated control. Moreover, the tested compound **4** induced an increase with 1.89-fold (6.29%, compared to 3.33% for control) in cell death via necrosis, as shown in Figure 2. The results proved that the tested compound **4** effectively induced apoptosis and necrosis in MCF-7 cells.

##### DNA Content-Flow Cytometry Aided Cell Cycle Analysis

**Figure 3:**
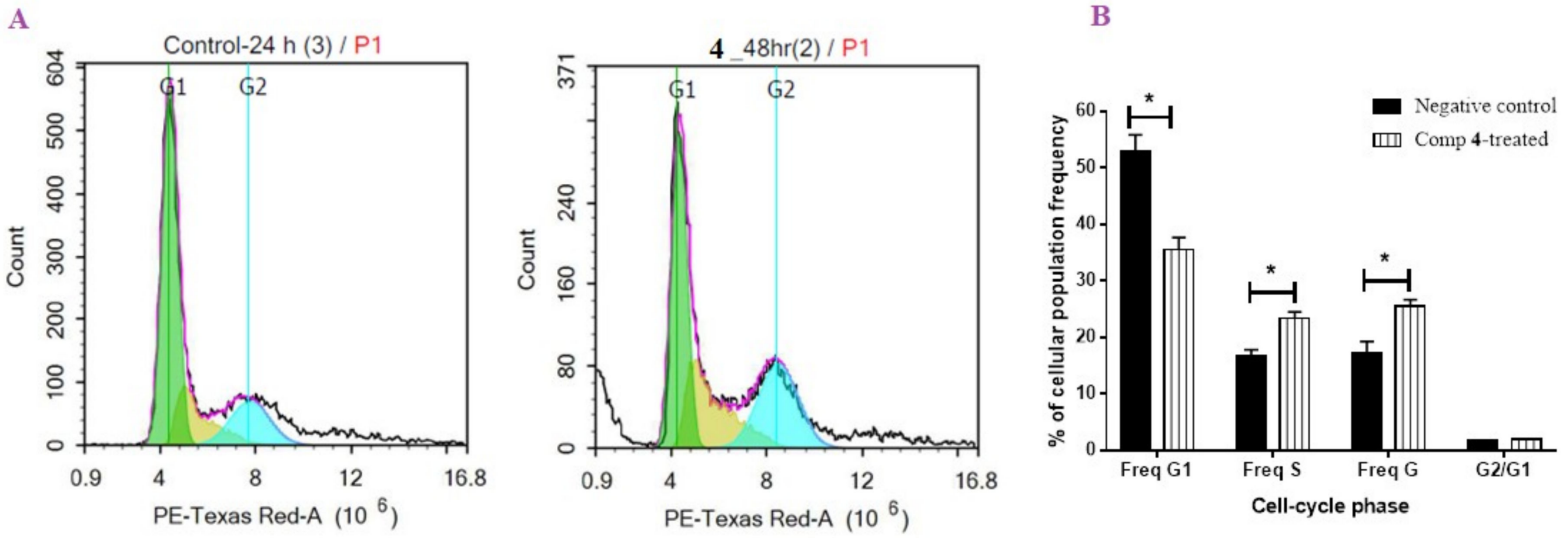
Cytogram reflecting the cell cycle distribution of MCF-7 cells (**A**): MCF-7 cells untreated and treated with compound **4** (23.2 μM, 48 h); (**B**) bar chart representation of the percentage of cell population in different MCF-7 cell cycle phases. * *p* < 0.05 unpaired *t*-test compared to negative control.

Cell cycle analysis is an important test that demonstrates the cell proliferation percentage during cell growth in every phase following cytotoxic compound treatment. Therefore, to analyze the cell cycle kinetics of MCF-7 cells treated with compound **4** (IC_50_ = 23.2 μM, 48h incubation), DNA flow cytometry was carried out to indicate phase interference with the cell cycle of the compound’s. As shown in (Figure 3), results showed an induction an increase at G2/M-phase cell-cycle arrest with 1.48-fold (25.56%, compared to 17.23% for control), and at S-phase cell-cycle arrest with 1.39-fold (23.38%, compared to 16.76% for control) in cell population distribution, this may have resulted in genetic material degradation due to the apoptosis induction indicating compound **4** antiproliferative.

##### Acridine Orange Quantitative Autophagy Assessment

**Figure 4:**
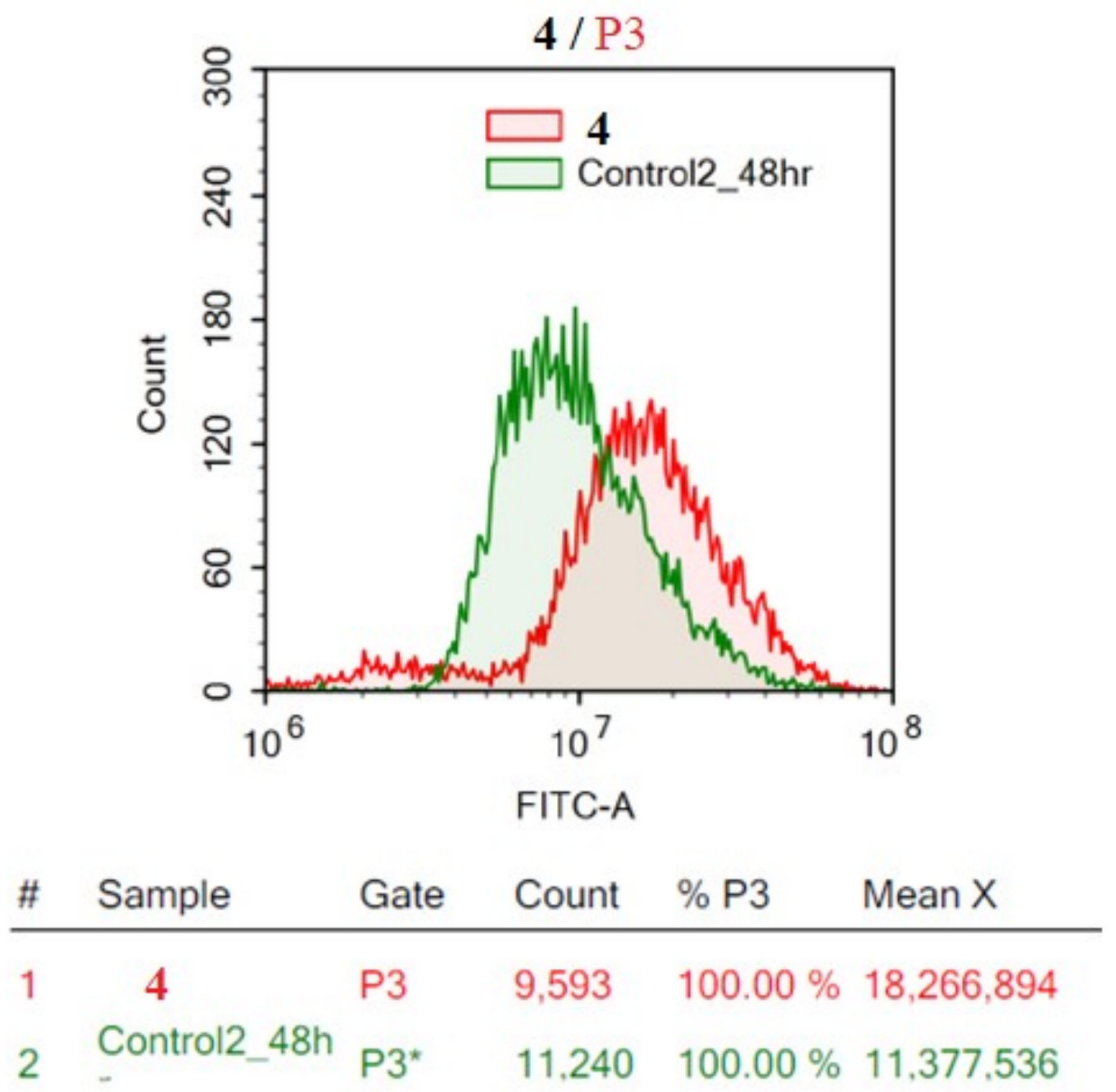
Autophagic cell death assessment in MCF-7 treated with compound **4** (IC_50_ = 23.2 μM) using the acridine orange lysosomal stain coupled with the flow cytometric analysis. The green curve for the negative control (untreated), and the red one for the compound **4**-treated cells.

Herein, we further investigated the effect of compound **4** (IC_50_ = 23.2 μM) on the autophagy process within MCF-7 using the acridine orange lysosomal stain coupled with the flow cytometric analysis. The tested compound **4** did not induce any significant cell death by autophagy, it inhibited autophagic cell death (9.59%, compared to 11.24% for control) and this proves the antiproliferative activity of compound **4** through apoptosis and necrosis as a dual activity (Figure 4).

#### 2.2.3. In Silico Molecular Docking Studies

A molecular docking study was made to investigate the binding interactions of hit **4** towards three proteins as JAK2 inhibitors; 3ZMM, 4C62, and 5AEP whose crystal structures complexed with their co-crystallized ligands were easily accessible from the Protein Data Bank. The co-crystallized ligands of the studied proteins form hydrogen bonds with Leu 932 as the key amino acid for interaction.

As seen in (Table 2) with 3D images, compound **4** was docked inside the protein active site of the studied proteins and formed one hydrogen bond with bond length (A^º^) through the carbonyl group oxygen as a hydrogen bond acceptor with the key amino acid Leu 932 as their co-crystallized ligand with binding energy −14.32, −13.39, and −11.38 Kcal/mol, respectively. Additionally, compound **4** formed lipophilic interactions with the nonpolar amino acids (Leu 155, Val 24, Ile 140, Leu 90, and Leu 141) inside the receptor pocket. Different models obtained from the docking studies indicated that the designed target **4** showed promising binding activity as JAK2 inhibitors, and this may be the proposed mode of action for the anti-breast cancer activity.

#### 2.2.4. Bioinformatics Study

**Figure 5:**
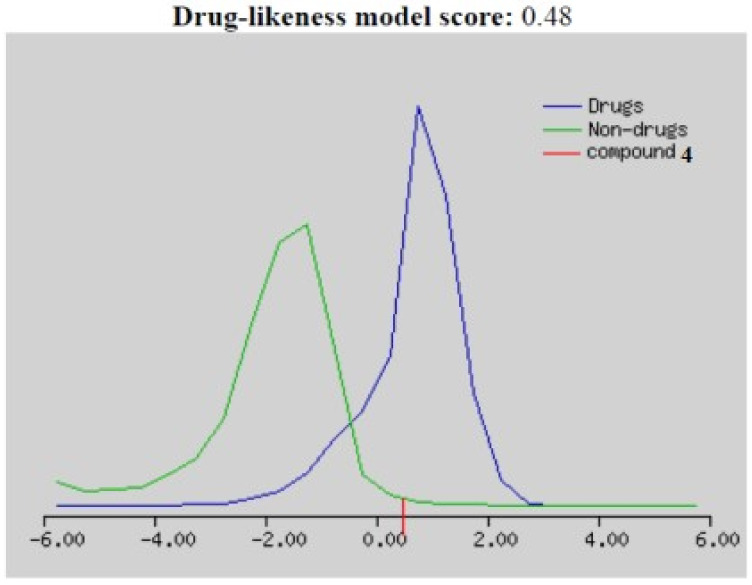
Plotting of Drug-likeness score of compound **4** using MolSoft. Non-drug like behavior (green-colored curve) and drug-like behavior (blue-colored curve). Compounds with zero or negative value should not seem to be as drug. Compound **4** has a value of 0.48 (positive value) as a drug-likeness score. It should be regarded as drug-like.

Compounds with high binding affinity through ligand-receptor interactions and binding energy towards the investigated target (Jak2/STAT3) were subjected to bioinformatics study to predict the ADME pharmacokinetics properties. Drugs consistent with Lipinski’s "five rule" (Ro5) are considered prospective in future [31,32,33,34]. For drug absorption through the intestine, TPSA surface topological polar area values should be 140, and the barrier to blood brain should be as low as 90 Å^2^ [30,31] The investigated compounds had good well-permeability and absorption. As shown in (Table 3) and (Figure 5), compounds had 0-1 Hydrogen-bond donor and 3-4 Hydrogen-bond acceptors. In addition, the tested compounds had log *P* range from 2.82 to 4.80, so they had strong toleration by cell membranes. For managing conformational changes and for oral bioavailability, the number of rotabile bond (nrotb) should be less than 10 [31,35,36]. All the investigated compounds had 1–5 nrotb. Regarding to drug-likeness scores, compounds with positive values should be considered like drugs; all the tested compounds showed 0.15–0.73 (positive values), so they seem to be drug-like.

#### 2.2.5. In Vivo Study

**Figure 6:**
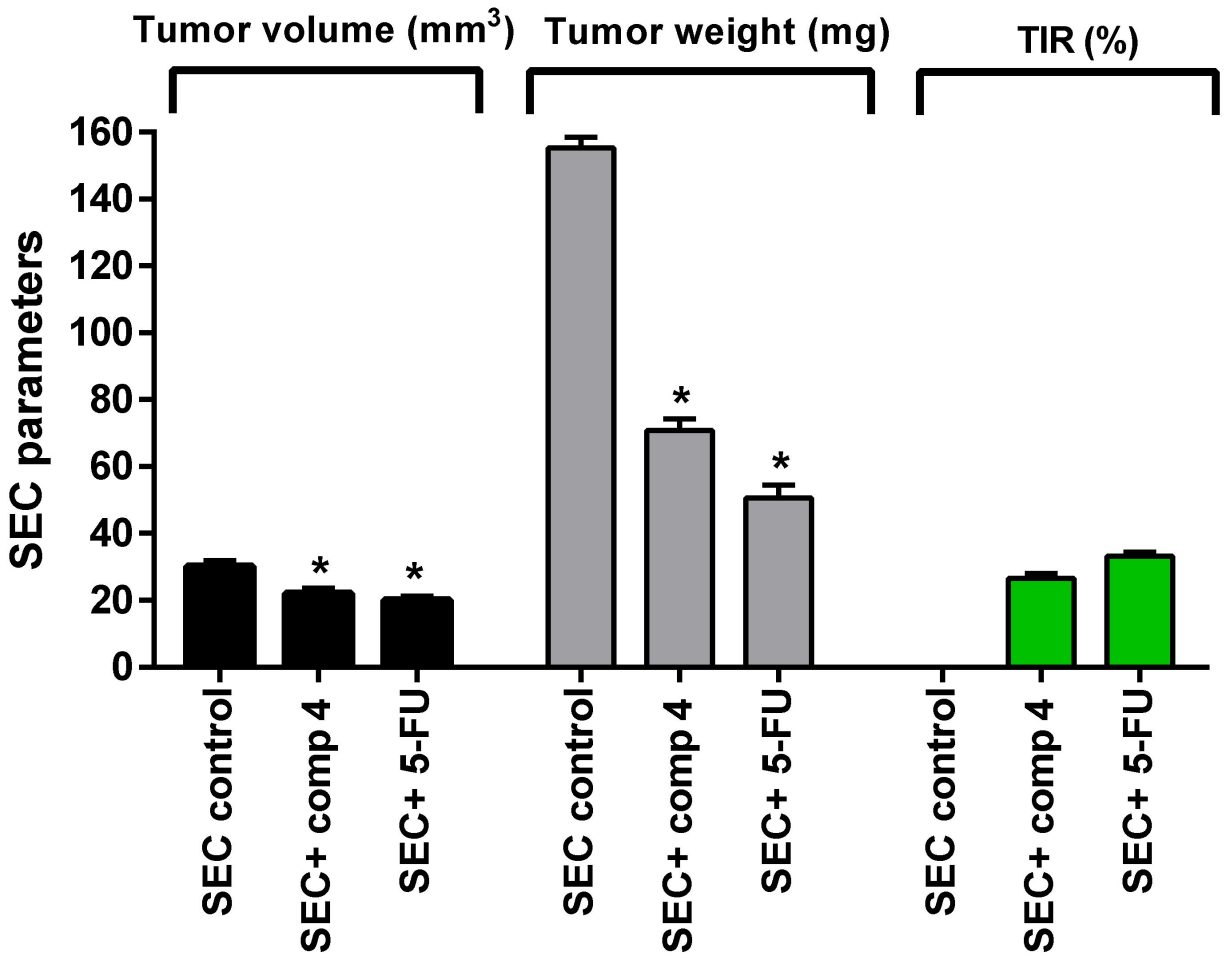
Solid Ehrlich Carcinoma (SEC)-bearing mice model. Different solid tumor mass, volume, tumor inhibition ratio (TIR %) values are attributed to the antitumor activity. Mean ± SEM values of mice in each group (n = 8). * Mean ± SEM values with a significant difference in tumor volume and tumor weight values compared to the SEC control using an unpaired *t*-test (*p* ≤ 0.05) using GraphPad prism.

Solid Ehrlich Carcinoma (SEC) was treated daily with compound **4** to evaluate the in vivo effect on tumor cell growth, beginning on day 7 following inoculation of tumor cells. The compound had a total of seven doses, starting on day 7. At the end of the procedure, the weight of the solid tumor mass was measured. There was an increase in the solid tumor mass in SEC-bearing mice. During the experimental duration, an increase in the solid tumor weight of around 155 mg was observed via the tumor development. The antitumor effect of compound **4** and 5-FU was elucidated; there was a significant decrease in the solid tumor mass by 54% (70 mg) and 67% (50 mg), respectively (Figure 6).

Accordingly, treatments with compound **4** significantly inhibited the percentage of tumor by 26.6% (tumor volume = 22 mm^3^) compared to the 5-FU treatment 33.3% (tumor volume = 20 mm^3^). The achieved results indicate that compound **4** has a promising therapeutic potential as an antitumor agent.

At the end of the experiment, animals from different groups were anesthetized, and blood samples were collected for the determination of liver enzymes ALT, AST levels, and CBC parameters, including Hb content, RBCs and WBCs counts. As seen in Table 4, in SEC-bearing mice, liver enzymes ALT and AST were found to be significantly increased to 84.65 and 97.64 (U/L), respectively, as compared to normal mice, this could be contributed to the hepatocellular damage following tumor inoculation. The treatment with compound **4**, such as 5-FU, substantially reduced liver enzyme, where elevated transaminases ALT were restored to 64.28, AST to 69.89 close to the normal values measured in normal mice (43.53 and 45.75, respectively), indicating a noticeable improvement in the hepatocellular toxicity induced by SEC proliferation.

Regarding hematological parameters in SEC-bearing mice, all CBC parameters were changed upon treatment with compound **4** where the Hb content and RBCs were significantly decreased to be 3.99 (g/dL) and 3.28 (10^6^/µL), respectively, while the WBCs count was significantly increased to be 4.89 (10^3^/µL) compared to the normal control levels. Myelosuppression and anemia are the main issues of cancer chemotherapy. Anemia in a tumor-bearing mouse is primarily due to reductions in the RBC count and hemoglobin, which are either hemolytic or myelopathic [30]. CBC parameters for hemoglobin, RBC, and WBC levels have been greatly improved by compound **4** treatments. CBC parameters improved to 5.99, 4.43, and 4.21, respectively, relative to normal control values 7.9, 5.14, and 3.69, hence demonstrating the ability of the tested compound to cure the change of the hematological parameters.

## 3. Materials and Methods

All general information about the equipment used in this text, biological activities assays (in vitro MTT assay, flow cytometric analysis, in silico molecular docking, bioinformatics study, and in vivo SEC model) of full protocols have been amended in the Supplementary Materials.

### 3.1. Synthesis

Synthesis of (**1**): Sulpher (0.02 mol) was added to a mixture of ethyl cyanoacetate (0.03 mol) and cyclohexanone (0.03 mol) in 30 mL of ethanol followed by the addition of Et_3_N (4.3 mmol), the mixture kept on stirring at an ambient temperature for 2 h, then refluxed for further 2 h. The reaction progress was monitored by TLC then, cooled, added to ice water, and kept overnight to complete the precipitation. The ppt was filtered, washed with water, dried, and recrystallized from ethanol to give yellow needle crystals.

*Ethyl 2-amino-4,5,6,7-tetrahydrobenzo[b]thiophene-3-carboxylate (**1**)*

Yield: 86%, m.p. 110–111 °C; ^1^H NMR (400 MH_Z_, DMSO-*d*_6_): *δ* 7.15 (s, 2H, D**_2_**O exchangeable, NH_2_), 4.15 (q, 2H, OC*H*_2_CH_3_), 2.42, 2.61 (2 m, 4H, 2 CH_2cyhex_), 1.67–1.69 (m, 4H, 2 CH_2cyhex_), 1.25 (t, 3H, *J* 6.0 Hz, CH**_3_**); ^13^C NMR (100 MH_Z_, DMSO-*d*_6_): *δ* 165.53, 163.33, 131.80, 115.97, 103.25 (4 C_thiophene_, C=O), 59.03 (OCH**_2_**), 26.98, 24.41, 23.31, 22.19 (4 CH_2cyhex_), 14.79 (CH_3_); CHN calcd. for: C_11_H_15_NO_2_S [225.08]: C, 58.64; H, 6.71; N, 6.22; S, 14.23; found: C, 58.54; H, 6.69; N, 6.26; S, 14.12.

Synthesis of (**2**) and (**3**): A mixture of amino-ester **1** (2.2 mmol) and the appropriate isothiocyanate (2.2 mmol) and Et_3_N 0.5 mL was refluxed in 30 mL of absolute ethanol for 3 h then left to cool. Acidified with concentrated HCl, the formed ppt was collected by filtrations, washed with water, dried, and recrystallized from ethanol.

*Ethyl 2-(3-phenylthioureido)-4,5,6,7-tetrahydrobenzo[b]thiophene-3-carboxylate (**2**)*

Yield: 73%, m.p. 173–174 °C; ^1^H NMR (400 MHz, DMSO-*d*_6_): δ 11.78 (s, 1H, D_2_O exchangeable, NH), 10.88 (s, 1H, D_2_O exchangeable, NHPh), 7.49 (d, 2H, Ph), 7.41 (t, 2H, Ph), 7.24 (t, 1H, Ph), 4.23 (q, 2H, OC*H_2_*CH_3_), 2.72 (s, 2H, CH_2cyhex_), 2.60 (s, 2H, 2 CH_2cyhex_), 1.73 (s, 4H, 2 CH_2cyhex_), 1.28 (t, 3H, OCH_2_C*H_3_*); ^13^C NMR (100 MHz, DMSO-*d*_6_): δ 176.36 (C=S), 166.02 (C=O), 150.00, 138.69, 130.49, 129.35, 126.34, 126.14, 124.78, 112.42, (4 C_thiophene_, 6 C_phenyl_), 60.72 (O*CH*_2_CH_3_), 26.34, 24.12, 23.01, 22.91, (4 CH_2cyhex_), 14.52 (CH_2_*CH_3_*); CHN calcd. for: C_18_H_20_N_2_O_2_S_2_ [360.49]: C, 59.97; H, 5.59; N, 7.77; S, 17.79; found: C, 59.95; H, 5.56; N, 7.73; S, 17.77.

*2-(Ethylamino)-5,6,7,8-tetrahydro-4H-benzo[4,5]thieno[2,3-d][1,3]thiazin-4-one (**3**)*

Yield: 70%, m.p. 265–267 °C; ^1^H NMR (400 MHz, DMSO-*d*_6_): δ 13.43 (s, 1H, D_2_O exchangeable, NH), 4.40 (q, 2H, NH*CH_2_*CH_3_), 2.78, 2.64 (2brs, 4H, 2 CH_2cyhex_), 1.75 (m, 4H, 2 CH_2cyhex_), 1.20 (t, 3H, CH_2_*CH_3_* ); ^13^C NMR (100 MHz, DMSO-*d*_6_): δ 173.56, 156.64 (C=O, C_thiazin_), 149.02, 131.38, 128.77, 116.17 (4 C_thiophene_), 41.08 (NH*CH_2_*CH_3_), 25.32, 24.40, 22.91, 22.01 (4 CH_2cyhex_), 12.26 (CH_2_*CH_3_*); CHN calcd. for: C_12_H_14_N_2_OS_2_ [266.38]: C, 54.11; H, 5.30; N, 10.52; S, 24.07; found: C, 54.22; H, 5.32; N, 10.50; S, 24.04.

Acylation with Chloroacetyl Chloride: A mixture of **1** (4.5 mmol) and chloroacetyl chloride (13.5 mmol) in 30 mL of methylene chloride containing Et_3_N (0.5 mL) was stirred for 3 h in an ice bath. The formed solid product was collected by filtration, dried, and recrystallized from ethanol.

*Ethyl 2-(2-chloroacetamido)-4,5,6,7-tetrahydrobenzo[b]thiophene-3-carboxylate (**4**)*

Yield: 75%, m.p. 110–112 °C; ^1^H NMR (400 MHz, DMSO-d_6_):δ 11.66 (s, 1H, D_2_O exchangeable, NH), 4.57 (s, 2H, *CH_2_*Cl), 4.32 (q, 2H, OCH_2_CH_3_), 2.71 (s, 4H, 2 CH_2cyhex_), 2.61 (s, 2H, 2 CH_2cyhex_), 1.73 (s, 4H, 2 CH_2cyhex_), 1.32 (t, 3H, CH_2_*CH_3_*); ^13^C NMR (100 MHz, DMSO-*d*_6_): δ 165.45, 164.23 (C=O_amide+ester_), 145.55, 131.18, 127.30, 112.78 (4 C_thiophene_), 61.00 (O*CH_2_*CH_3_), 43.01 (*CH_2_*Cl), 26.24, 24.20, 22.89, 22.70 (4 CH_2cyhex_), 14.52 (CH_2_*C*H_3_); CHN calcd. for: C_13_H_16_ClNO_3_S [301.79]: C, 51.74; H, 5.34; N, 4.64; S, 10.62; found: C, 51.76; H, 5.30; N, 4.62; S, 10.63.

Synthesis of (**5**): A mixture of **4** (1.6 mmol) and cyclohexylamine (1.6 mmol) was refluxed in 30 mL of absolute ethanol for 3 h and cooled to room temperature. The formed solid was filtered, dried, and recrystallized from ethanol.

*Ethyl 2-(2-(cyclohexylamino)acetamido)-4,5,6,7-tetrahydrobenzo[b]thiophene-3-carboxylate (**5**)*

Yield: 69%, m.p. 140–141 °C; ^1^H NMR (400 MHz, DMSO-*d*_6_): δ 1.29–4.69 (m, 28H); ^13^C NMR (100 MHz, DMSO-*d*_6_): δ 171.22, 164.75 (2 C=O_amide+ester_), 146.07, 131.04, 126.12, 111.77 (4 C_thiophene_,), 60.46 (O*CH*_2_CH_3_), 57.35, 49.99, 33.25, 26.33, 26.17, 24.89, 24.26, 23.01, 22.85 (9 CH_2cyhex_, CH_cyhex_), 14.67 (CH_2_*CH_3_*); CHN calcd. for: C_19_H_28_N_2_O_3_S [364.50]: C, 62.61; H, 7.74; N, 7.69; S, 8.80; found: C, 62.60; H, 7.70; N, 7.66; S, 8.81.

Synthesis of (**6**): A mixture of **1** (3.8 mmol), benzoyl chloride (4.3 mmol), and few drops of triethylamine was refluxed for 4 h in dry benzene. The reaction mixture was concentrated and cooled at room temperature. The formed ppt was collected and recrystallized from ethanol to obtain yellow crystals.

*Ethyl 2-benzamido-4,5,6,7-tetrahydrobenzo[b]thiophene-3-carboxylate (**6**)*

Yield: 8%, m.p. 170–172 °C; ^1^H NMR (400 MHz, DMSO-*d*_6_): δ 12.03 (s, 1H, D_2_O exchangeable, NH), 7.64–7.93 (m, 5H, phenyl), 4.35 (q, 2H, O*CH_2_*CH_3_), 2.66, 2.76 (2brs, 4H, 2 CH_2cyhex_), 1.76 (brs, 4H, 2 CH_2cyhex_), 1.35 (t, 3H, CH_2_*CH*_3_); ^13^C NMR (100 MHz, DMSO-*d*_6_): δ 166.23, 163.06 (2 C=O_ester+amide_)_,_ 147.01, 133.21, 132.54, 131.21, 129.64, 127.46, 127.03 (4 C_thiophene_, 6 C_phenyl_), 61.04 (O*CH_2_*CH_3_), 26.29, 24.27, 22.97, 22.75 (4 CH_2cyhex_), 14.52 (OCH_2_*CH_3_*); CHN calcd. for: C_18_H_19_NO_3_S [329.41]: C, 65.63; H, 5.81; N, 4.25; S, 9.73; found: C, 65.60; H, 5.84; N, 4.15; S, 9.5.

Synthesis of (**7**): A mixture of compound **1** (2.2 mmol) and benzenesulfonyl chloride (2.2 mmol) was refluxed in 30 mL of absolute ethanol for 4 h then cooled to room temperature. The ppt was collected by filtration, washed with ethanol, and recrystallized from ethanol.

*Ethyl 2-(phenylsulfonamido)-4,5,6,7-tetrahydrobenzo[b]thiophene-3-carboxylate (**7**)*

Yield: 73%, m.p. 107–108 °C; ^1^H NMR (400 MHz, DMSO-*d*_6_): δ 10.36 (s, 1H, NH), 7.81 (d, 2H, Ph), 7.68 (t, 1H, Ph), 7.60 (t, 2H, Ph), 4.14 (q, 2H, O*CH**_2_***CH_3_), 2.56–2.59 (m, 4H, 2 CH_2cyhex_), 1.68–1.69 (m, 4H, 2 CH_2cyhex_), 1.21 (t, 3H, OCH_2_*CH_3_*); ^13^C NMR (100 MHz, DMSO-*d*_6_): δ 163.84 (C=O_ester_), 142.70, 139.46, 133.87, 132.91, 130.13, 129.82, 127.29, 120.47 (4 C_thiophene+_, 6 C_phenyl_), 60.76 (O*CH_2_*CH_3_), 25.92, 24.47, 22.81, 22.42 (4 CH_2cyhex_), 14.38 (OCH_2_*CH_3_*); CHN calcd. for: C_17_H_19_NO_4_S_2_ [365.46]: C, 55.87; H, 5.24; N, 3.83; S, 17.54; found: C, 55.88; H, 5.26; N, 3.80; S, 17.56.

Synthesis of (**8**): A mixture of **1** (4.5 mmol) and formamide (0.44 mol) was refluxed for 3 h. Cooled, poured onto cold water, and left at room temperature overnight, the formed ppt was filtered off and recrystallized from methanol to give brown needle crystals.

*4,5,6,7,8-Tetrahydrobenzo[4,5]thieno[2,3-d]pyrimidin-4(3H)-one (**8**)*

Yield: 90%, m.p. 250–251 °C; **^1^**H NMR (400 MHz, DMSO-*d*_6_): *δ* 12.20 (s, 1H, D**_2_**O exchangeable, NH), 7.96 (s, 1H, CH_pyrimid._), 2.72, 2.86 (2brs, 4H, 2 CH_2cyhex_), 1.74–1.80 (m, 4H, 2 CH_2cyhex_); ^13^C NMR (100 MHz, DMSO-*d*_6_): *δ* 162.89, 158.1, 145.2, 132.57, 131.3, 123.2 (4 C_thiophene_, 2 C_pyrimidone_), 25.79, 24.90, 22.94, 22.24 (4 CH_2cyhex_); CHN calcd. for: C_10_H_10_N_2_OS [206.26]: C, 58.23; H, 4.89; N, 13.58; S, 15.54; found: C, 58.25; H, 4.90; N, 13.57; S, 15.50.

Michael Addition Procedures: A mixture of pyrimidinone **8** (3.4 mmol), acrylonitrile (4.6 mmol), and Et_3_N (0.3 mL) in 25 mL of ethanol was refluxed for 6 h, left to cool, a solid product formed, filtered off, and recrystallized from ethanol to give colorless crystals.

*3-(4-Oxo-5,6,7,8-tetrahydrobenzo[4,5]thieno[2,3-d]pyrimidin-3(4H)-yl)propanenitrile (**9**)*

Yield: 73%, m.p. 173–175 °C; ^1^H NMR (400 MHz, DMSO-*d*_6_): δ 8.37 (s, 1H, CH_pyrimidine_), 4.23–4.25 (m, 2H, NCH_2_), 3.02–3.04 (m, 2H, CH_2_C*H*_2_CN), 2.89 (brs, 2H, CH_2cyhex_), 2.75 (brs, 2H, CH_2cyhex_), 1.79 (brs, 4H, 2 CH_2cyhex_); ^13^C NMR (100 MHz, DMSO-*d*_6_): δ 162.10 (C=O _pyrimidone_), 157.20, 147.55, 133.79, 131.35, 122.15, 118.56 (6 C_(1pyrimidone+4thiophene+CN)_), 41.84 (N*C*H_2_), 25.77, 25.02, 22.85, 22.20 (4 CH_2cyhex_), 17.49 (*C*H_2_CN); CHN calcd. for: C_13_H_13_N_3_OS [259.33]: C, 60.21; H, 5.05; N, 16.20; S, 12.36; found: C, 60.23; H, 5.10; N, 16.23; S, 12.34.

General Procedure for Preparation of Compounds (**10**–**13**): To a mixture of pyrimidinone **8** (3.0 mmol), the appropriate alkyl halide (3.3 mmol) and anhydrous potassium carbonate (3.6 mmol) was refluxed in 20 mL of DMF for 4 h. Then cooled, poured in cold water, the ppt was filtered off and recrystallized from ethanol to afford the alkylated products **10**–**13**.

*3-Benzyl-5,6,7,8-tetrahydrobenzo[4,5]thieno[2,3-d]pyrimidin-4(3H)-one (**10**)*

Yield: 73%, m.p. 190 °C; ^1^H NMR (400 MHz, DMSO-*d*_6_): δ 8.47 (s, 1H, CH_pyrimidine_), 7.34 (s, 5 H_phenyl_), 5.18 (s, 2H, NCH_2_), 2.90–2.89 (m, 2H, CH_2cyhex_), 2.75–2.74 (m, 2H, CH_2cyhex_), 1.81–1.75 (m, 4H, 2 CH_2cyhex_); ^13^C NMR (100 MHz, DMSO-*d*_6_): δ 162.01 (C=O_pyrimidone_), 157.32, 147.92, 137.36, 133.62, 131.38, 129.07, 128.09, 122.42 (11 C_(1pyrimidone+4thiophene+6phenyl)_), 48.83 (NCH_2_), 25.81, 25.01, 22.84, 22.21 (4 CH_2cyhex_); CHN calcd. for: C_17_H_16_N_2_OS [296.39]: C, 68.89; H, 5.44; N, 9.45; S, 10.82; found: C, 68.88; H, 5.46; N, 9.43; S, 10.80.

*3-(2-Oxo-2-phenylethyl)-5,6,7,8-tetrahydrobenzo[4,5]thieno[2,3-d]pyrimidin-4(3H)-one (**11**)*

Yield: 85% pale brown crystals, m.p. 190–192 °C; ^1^H NMR (400 MHz, DMSO-*d*_6_ ): δ 8.29 (s, 1H, CH_pyrimidine_), 8.10 (d, 2 H_phenyl_), 7.75 (t, 2 H_phenyl_), 7.62 (t, 1 H_phenyl_), 5.60 (s, 2H, NCH_2_), 2.86 (brs, 2H, CH_2cyhex_), 2.78 (brs, 2H, CH_2cyhex_), 1.78–1.83 (m, 4H, 2CH_2cyhex_); ^13^C NMR (100 MHz, DMSO-*d*_6_ ): δ 193.25 (*C*=O_phenacyl_), 162.22 (C=O_pyrimidine_), 157.26, 148.28, 134.91, 134.59, 133.67, 131.33, 129.47, 128.50, 122.20 (11 C_(4thiophene+ 1pyrimidine+6phenyl)_), 52.05 (N*C*H_2_), 25.76, 25.03, 22.88, 22.22 (4 CH_2cyhex_); CHN calcd. for: C_18_H_16_N_2_O_2_S [324.40]: C, 66.64; H, 4.97; N, 8.64; S, 9.88; found: C, 66.60; H, 4.95; N, 8.66; S, 9.90.

*3-(2-(4-methoxyphenyl)-2-oxoethyl)-5,6,7,8-tetrahydrobenzo[4,5]thieno[2,3-d]pyrimidin-4(3H)-one (**12**)*

Yield: 90% as yellow crystals, m.p. 145–146 °C; ^1^H NMR (400 MHz, DMSO-*d*_6_ ): δ 8.27 (s, 1H, CH_pyrimidine_), 8.08 (d, 2 H_phenyl_), 7.14 (d, 2 H_phenyl_), 5.53 (s, 2H, NC*H*_2_), 3.90 (s, 3H, OC*H*_3_ ), 2.86 (s, 2H, CH_2cyhex_), 2.78 (s, 2H, CH_2cyhex_), 1.77–1.81 (m, 4H, 2 CH_2cyhex_); ^13^C NMR (100 MHz, DMSO-*d*_6_): δ 191.48 (C=O_phenacyl_), 164.35 (C=O_pyrimidine_), 162.24, 157.29, 148.36, 133.58, 131.32, 130.88, 127.81, 122.20, 114.72 (11 C_(4thiophene+ 1pyrimidine+6phenyl)_), 56.14 (O*C*H_3_), 51.64 (N*C*H_2_), 25.76, 25.02, 22.88, 22.22 (4 *C*H_2cyhex_); CHN calcd. for: C_19_H_18_N_2_O_3_S [354.42]: C, 64.39; H, 5.12; N, 7.90; S, 9.05; found: C, 64.41; H, 5.14; N, 7.93; S, 9.00.

*Ethyl 2-(4-oxo-5,6,7,8-tetrahydrobenzo[4,5]thieno[2,3-d]pyrimidin-3(4H)-yl)acetate (**13**)*

Yield: 82% pale brown needles, m.p. 113–114 °C; ^1^H NMR (400 MHz, DMSO-*d*_6_): δ 8.30 (s, 1H, CH_pyrimdine_), 4.79 (s, 2H, NC*H_2_*), 4.18 (q, *J*=8.0 Hz, 2H, OC*H_2_*CH_3_), 2.87 (brs, 2H, CH_2cyhex_), 2.65 (brs, 2H, CH_2cyhex_), 1.79 (m, 4H, 2CH_2cyhex_), 1.23 (t, 3H, CH_3_); ^13^C NMR (100 MHz, DMSO-*d*_6_): δ 168.29 (C=O_ester_), 162.17 (C=O_pyrimidine_), 157.21, 147.95, 133.85, 131.29, 122.08 (4 C_thiophene_, 1 C_pyrimidine_), 61.71 (O*C*H_2_CH_3_), 47.25 (N*C*H_2_), 25.72, 25.00, 22.85, 22.19 (4 CH_2cyhex_), 14.45 (CH_2_*C*H_3_); CHN calcd. for: C_14_H_16_N_2_O_3_S [292.35]: C, 57.52; H, 5.52; N, 9.58; S, 10.97; found: C, 57.48; H, 5.50; N, 9.61; S, 10.99.

Synthesis of (**14**): A mixture of ester **13** (3.0 mmol) and hydrazine hydrate (6.0 mmol) in 30 mL of ethanol was refluxed for 3 h, the reaction mixture was cooled, the solid product was filtered off, dried, and recrystallized from EtOH/DMF.

*2-(4-Oxo-5,6,7,8-tetrahydrobenzo[4,5]thieno[2,3-d]pyrimidin-3(4H)-yl)acetohydrazide (**14**)*

Yield: 91% as white crystals, m.p. 246–248 °C; ^1^H NMR (400 MHz, DMSO-d*_6_*): δ 9.36 (s, 1H, D_2_O exchangeable, NH), 8.22 (s, 1H, CH_pyrimdine_), 4.58 (s, 2H, NC*H*_2_), 4.27 (s, 2H, D_2_O exchangeable, NH_2_), 2.86 (brs, 2H, CH_2cyhex_), 2.76 (brs, 2H, CH_2cyhex_), 1.77–1.80 (m, 4H, 2 CH_2cyhex_); ^13^C NMR (100 MHz, DMSO-*d*_6_): δ 166.55, 162.13 (2 C=O_(amide+pyrimidone)_), 157.32, 148.55, 133.36, 131.28, 122.13 (5 C_(1pyrimidine+4thiophene)_), 46.85 (N*C*H_2_), 25.77, 25.00, 22.88, 22.24 (4 CH_2cyhex_); CHN calcd. for: C_12_H_14_N_4_O_2_S [278.33]: C, 51.78; H, 5.07; N, 20.13; S, 11.52; found: C, 51.80; H, 5.17; N, 20.11; S, 11.55.

Synthesis of (**15**): To a mixture of hydrazide **14** (3.0 mmol) in 30 mL of dioxane, phenyl isothiocyanate (3.7 mmol) was added, the reaction mixture was kept under reflux for 6 h, concentrated, cooled, and left overnight. The formed ppt was collected and recrystallized from ethanol to give **15** as white crystals.

*2-(2-(4-Oxo-5,6,7,8-tetrahydrobenzo[4,5]thieno[2,3-d]pyrimidin-3(4H)-yl)acetyl)-N-phenylhydrazine-1-carbothioamide (**15**)*

Yield: 85%, m.p. 155–156 °C; ^1^H NMR (400 MHz, DMSO-*d*_6_): δ 10.56, 9.77, 9.33 (3s, 3H, D_2_O exchangeable, 3NH), 8.30 (s, 1H, CH_pyrimidone_), 7.59 (d, 2H, *J* 8 Hz, 2HAr), 7.35 (t, 2H, 2HAr), 7.17–7.20 (m, 1H, 1HAr ), 4.74 (s, 2H, NC*H*_2_), 2.81 (brs, 2H, CH_2cyhex_), 2.77 (brs, 2H, CH_2cyhex_), 1.74–1.80 (m, 4H, 2 CH_2cyhex_); ^13^C NMR (100 MHz, DMSO-*d*_6_): δ 181.06 (C=S), 167.10, 162.5 (2 C=O_pyrimidone+amide_), 157.88, 148.23, 139.38, 133.91, 131.16, 128.57, 125.55, 125.21, 122.12 (11 C_(1pyrimidone+4thiophene+6phenyl)_), 47.98 (N*C*H_2_), 25.76, 25.01, 22.84, 22.18 (4 CH_2cyhex_); CHN calcd. for: C_19_H_19_N_5_O_2_S_2_ [413.51]: C, 55.19; H, 4.63; N, 16.94; S, 15.51; found: C, 55.23; H, 4.60; N, 16.92; S, 15.50.

General Procedures for Synthesis of Compounds (**16**) and (**17**): To a mixture of hydrazide (3.0 mmol) in 30 mL of ethanol, the selected aldehyde or ketone (3.3 mmol) was added and refluxed for 6 h. Then, concentrated and left overnight, the formed ppt was recrystallized from ethanol to give **16** and **17**.

*(E)-N’-Benzylidene-2-(4-oxo-5,6,7,8-tetrahydrobenzo[4,5]thieno[2,3-d]pyrimidin-3(4H)-yl)acetohydrazide (**16**)*

Yield: 52%, m.p. 230 °C; ^1^H NMR (400 MHz, DMSO-*d*_6_): δ 11.65 (s, 1H, D_2_O exchangeable, NH), 8.29 (s, 1H, N=CH), 8.08 (s, 1H, CH_pyrimidone_), 7.73–7.74 (m, 2H, Ph), 7.46 (brs, 3H, Ph), 5.20 (s, 2H, NC*H*_2_), 2.89 (brs, 2H, CH_2cyhex_), 2.78 (brs, 2H, CH_2cyhex_), 1.79–1.84 (m, 4H, 2CH_2cyhex_); ^13^C NMR (100 MHz, DMSO-*d*_6_): δ 168.69, 162.16 (2 C=O_amide+pyrimidone_), 157.46, 148.61, 144.78, 134.37, 133.52, 131.30, 130.55, 129.33, 127.38, 122.16 (12 C_(1pyrimidone+4thiophene+ 6phenyl+N=CH)_), 46.94 (N*C*H_2_), 25.78, 25.01, 22.87, 22.23 (4 *C*H_2cyhex_); CHN calcd. for: C_19_H_18_N_4_O_2_S [366.44]: C, 62.28; H, 4.95; N, 15.29; S, 8.75; found: C, 62.30; H, 4.92; N, 15.31; S, 8.73.

*(E)-2-(4-Oxo-5,6,7,8-tetrahydrobenzo[4,5]thieno[2,3-d]pyrimidin-3(4H)-yl)-N’-(1-phenylethylidene)acetohydrazide (**17**)*

Yield: 50%, m.p. 230 °C; ^1^H NMR (400 MHz, DMSO-*d*_6_): δ 11.00 (s, 1H, D_2_O exchangeable, NH), 8.30 (s, 1H, CH_pyrimidone_), 7.80–7.86 (m, 2H, 2HAr), 7.43 (brs, 3H, 3HAr), 5.23 (s, 2H, N*CH_2_*), 2.88 (brs, 2H, CH_2cyhex_), 2.78 (brs, 2H, CH_2cyhex_), 2.31 (s, 3H, C*H*_3_), 1.79–1.81 (m, 4H, 2 CH_2cyhex_); ^13^C NMR (100 MHz, DMSO-*d*_6_): δ 169.55, 162.19 (C=O_amide+pyrimidone_), 157.50, 149.44, 148.59, 138.32, 133.56, 131.29, 129.77, 128.90, 126.83, 126.58, 122.17 (12 C_(N=C+1pyrimidone+4thiophene+6phenyl)_), 47.37 (*NC*H_2_), 25.77, 25.00, 22.85, 22.21 (4 CH_2cyhex_), 14.09 (CH_3_); CHN calcd. for: C_20_H_20_N_4_O_2_S [380.46]: C, 63.14; H, 5.30; N, 14.73; S, 8.43; found: C, 63.12; H, 5.33; N, 14.75; S, 8.42.

Synthesis of (**19**): To hydrazide **14** (3.0 mmol) in 30 mL of absolute ethanol containing aq. KOH (6.0 mmol), carbon disulphide (6.0 mmol) and the mixture was stirred at room temperature for 3 h, then the solvent was removed on a water bath to get the yellow ppt of **18,** which was directly used for the next step without further purification. To potassium salt **18** (3.0 mmol) in 10 mL of water, hydrazine hydrate (6.0 mmol) was added and the mixture was refluxed for 5 h, then cooled, diluted with water, and acidified by concentrated HCl. The formed ppt was collected, dried, and recrystallized from ethanol to give white plates crystals from **19**.

*3-((4-Amino-5-thioxo-4,5-dihydro-1H-1,2,4-triazol-3-yl)methyl)-5,6,7,8-tetrahydrobenzo[4,5]thieno[2,3-d]pyrimidin-4(3H)-one (**19**)*

Yield: 77%, m.p. 269–271 °C; ^1^H NMR (400 MHz, DMSO-*d*_6_ ): δ 13.54 (s, 1H, exchangeable, NH), 8.38 (s, 1H, CH_pyrimidine_), 5.61 (s, 2H, D_2_O exchangeable, NH_2_), 5.25 (s, 2H, NC*H*_2_), 2.87 (t, 2H, CH_2cyhex_), 2.77 (t, 2H, CH_2cyhex_), 1.75–1.83 (m, 4H, 2 CH_2cyhex_); ^13^C NMR (100 MHz, DMSO-*d*_6_): δ 167.32 (C=S_thioamide_), 162.06 (C=O_pyrimidone_), 157.03, 148.62, 148.14, 133.80, 131.35, 122.17 (6 C_(triazole+pyrimidine+4thiophene_), 40.2 (NCH_2_), 25.75, 25.01, 22.84, 22.19 (4 CH_2cyhex_); CHN calcd. for: C_13_H_14_N_6_OS_2_ [334.42]: C, 46.69; H, 4.22; N, 25.13; S, 19.18; found: C, 46.66; H, 4.20; N, 25.15; S, 19.21.

Synthesis of (**20**): The benzothieno[2,3-*d*]pyrimidine **8** (4.5 mmol) was refluxed in POCl_3_ (20 mL) for 3 h. The mixture was cooled and added into ice/water. The formed ppt was filtered off, washed several times with water, dried, and recrystallized from EtOH to give colorless needle crystals.

*4-Chloro-5,6,7,8-tetrahydrobenzo[4,5]thieno[2,3-d]pyrimidine (**20**)*

Yield: 64%, m.p. 110–112 °C; ^1^H NMR (400 MHz, DMSO-*d*_6_): δ 8.78 (s, 1H, CH_pyrimdine_), 2.98 (s, 2H, CH_2cyhex_), 2.88 (s, 2H, CH_2cyhex_), 1.85 (s, 4H, 2 CH_2cyhex_); ^13^C NMR (100 MHz, DMSO-*d*_6_): δ 158.00, 145.25, 132.65, 131.31, 123.22 (5 C_CHpyrimidine+4Cthiophene)_), 25.76, 24.90, 22.92, 22.22 (4 CH_2cyhex_); CHN calcd. for: C_10_H_9_ClN_2_S [224.70]: C, 53.45; H, 4.04; N, 12.47; S, 14.27; found: C, 53.47; H, 4.03; N, 12.49; S, 14.25.

Synthesis of (**21**): A mixture **20** (3.6 mmol) and hydrazine hydrate (0.03 mmol) in 35 mL of ethanol was refluxed for 5 h, then concentrated to half, the formed solid product was filtered off, dried, and recrystallized from ethanol.

*4-Hydrazineyl-5,6,7,8-tetrahydrobenzo[4,5]thieno[2,3-d]pyrimidine (**21**)*

Yield: 85% as pale brown crystals, m.p. 175–176 °C; ^1^H NMR (400 MHz, DMSO-*d*_6_): δ 8.33 (s, 2H, CH_pyrimidine_), 7.81 (s, 1H, D_2_O exchangeable, NH), 4.55 (s, 2H, D_2_O exchangeable, NH_2_), 2.93 (brs, 2H, CH_2cyhex_), 2.76 (brs, 2H, CH_2cyhex_), 1.80 (brs, 4H, 2 CH_2cyhex_); ^13^C NMR (100 MHz, DMSO-*d*_6_): δ 164.42, 158.76 (2 C_pyrimidine_), 152.93, 131.97, 127.26, 115.32 (4 C_thiophene_), 25.89, 25.35, 22.63, 22.50 (4 CH_2cyhex_); CHN calcd. for: C_10_H_12_N_4_S [220.29]: C, 54.52; H, 5.49; N, 25.43; S, 14.55; found: C, 54.49; H, 5.51; N, 25.44; S, 14.57.

Synthesis of (**22**): To hydrazide **21** (1.8 mmol) in 20 mL of absolute ethanol, benzoyl chloride (2 mmol) was added, the reaction mixture was refluxed for 6 h, then cooled. The formed solid product was filtered off and recrystallized from acetic acid.

*N’-(5,6,7,8-tetrahydrobenzo[4,5]thieno[2,3-d]pyrimidin-4-yl)benzohydrazide (**22**)*

Yield: 68% brown crystals, m.p. 198–200 °C; ^1^H NMR (400 MHz, DMSO-*d*_6_): δ 9.64 (s, 1H, NH), 8.29 (s, 2H, CH_pyrimdine+NH_), 7.59–7.60 (m, 5H, Ar), 3.22 (brs, 2H, CH_2cyhex_), 2.97 (brs, 2H, CH_2cyhex_), 1.91–1.95 (m, 4H, 2 CH_2cyhex_); ^13^C NMR (100 MHz, DMSO-*d*_6_): δ 164.38 (C=O), 153.33, 149.51, 138.69, 137.41, 131.13, 130.43, 129.44, 129.18, 127.66, 120.05 (12 C_(2pyrimdine+ thiophene+phenyl)_), 25.40, 23.00, 22.17, 21.44 (4 CH_2cyhex_); CHN calcd. for: C_17_H_16_N_4_OS [324.40]: C, 62.94; H, 4.97; N, 17.27; S, 9.88; found: C, 62.90; H, 4.96; N, 17.24; S, 9.85.

The General Procedure for Synthesis of Compounds (**23**) and (**24**): A mixture of **21** (1.6 mmol) and the selected aldehyde or ketone (2.0 mmol) in 20 mL of ethanol, was refluxed for 4 h, on cooling, the solid product was filtered off, dried, and recrystallized from ethanol to give compounds **23** and **24**.

*(E)-4-(2-benzylidenehydrazineyl)-5,6,7,8-tetrahydrobenzo[4,5]thieno[2,3-d]pyrimidine (**23**)*

Yield: 80% pale brown needles, m.p. 108–110 °C; ^1^H NMR (400 MHz, DMSO-*d*_6_): δ 11.79 (s, 1H, D_2_O exchangeable, NH), 8.40 (s, 1H, CH_pyrimidine_), 7.94 (brs, 2 H_phenyl_), 7.80 (s, 1H, N=C*H*-Ph), 7.39–7.46 (m, 3 H_phenyl_), 3.01 (brs, 2H, CH_2cyhex_), 2.76 (brs, 2H, CH_2cyhex_), 1.80 (brs, 4H, 2 CH_2cyhex_); ^13^C NMR (100 MHz, DMSO-*d*_6_): δ 157.81, 153.32 (2 C_pyrimidine_), 149.51, 144.31, 135.91, 132.78, 131.35, 130.33, 129.80, 128.55, 119.37 (11 C_(4thiophene+7N=*C*H-*C*6H5)_), 26.99, 25.20, 22.86, 22.52 (4 CH_2cyhex_); CHN calcd. for: C_17_H_16_N_4_S [308.40]: C, 66.21; H, 5.23; N, 18.17; S, 10.40; found: C, 66.20; H, 5.26; N, 18.20; S, 10.20.

*(E)-4-(2-(1-phenylethylidene)hydrazineyl)-5,6,7,8-tetrahydrobenzo[4,5]thieno[2,3-d]pyrimidine (**24**)*

Yield: 85% red crystals, m.p. 178–180 °C; ^1^H NMR (400 MHz, DMSO-*d*_6_): δ 11.49 (s, 1H, D_2_O exchangeable, NH ), 8.02 (brs, 2 H_phenyl_), 7.78 (s, 1H, CH_pyrimidine_), 7.41–7.43 (m, 3 H_phenyl_), 3.04 (brs, 2H, CH_2cyhex_), 2.75 (brs, 2H, CH_2cyhex_), 2.45 (s, 3H, C*H*_3_), 1.81 (brs, 4H, 2 CH_2cyhex_); ^13^C NMR (100 MHz, DMSO-*d*_6_): δ 157.97, 157.32, 147.94, 144.42, 139.20, 132.73, 131.34, 129.36, 128.53, 127.01, 120.14 (13 C _(4thiophene+6phenyl+2pyrimidine+N=C_), 26.87, 25.18, 22.88, 22.65 (4 CH_2cyhex_), 14.68 (CH_3_); CHN calcd. for: C_18_H_18_N_4_S [322.43]: C, 67.05; H, 5.63; N, 17.38; S, 9.94; found: C, 67.04; H, 5.66; N, 17.34; S, 9.99.

Synthesis of (**25**) and (**26**): Hydrazino derivative **21** (2.7 mmol) was refluxed in a glacial acetic acid or formic acid (3.5 mmol) for 4 h, then concentrated, cooled, and poured into cold water. The formed ppt was filtered off, washed with water, dried, and recrystallized from ethanol.

*3-Methyl-8,9,10,11-tetrahydrobenzo[4,5]thieno[3,2-e][1,2,4]triazolo[4,3-c]pyrimidine (**25**)*

Yield: 80%, m.p. 180 °C; ^1^H NMR (400 MHz, DMSO-*d*_6_): δ 9.48 (s, 1H, CH_pyrimidine_), 3.05 (brs, 2H, CH_2cychex_), 2.91 (brs, 2H, CH_2cychex_), 2.56 (s, 3H, CH_3_), 1.90 (brs, 4H, 2 CH_2cyhex_); ^13^C NMR (100 MHz, DMSO-d*_6_*): δ 164.68, 153.05, 148.96 (3 C_(2pyrimidine+1triazol)_), 138.35, 136.97, 128.92, 119.96 (4 C_thiophene_), 25.33, 22.95, 22.15 (4 CH_2cyhex_), 14.64 (*C*H_3_); CHN calcd. for: C_12_H_12_N_4_S [344.32]: C, 58.99; H, 4.95; N, 22.93; S, 13.12; found: C, 58.98; H, 4.97; N, 22.90; S, 13.05.

*8,9,10,11-Tetrahydrobenzo[4,5]thieno[3,2-e][1,2,4]triazolo[4,3-c]pyrimidine (**26**)*

Yield: 65%, m.p. 136 °C; ^1^H NMR (400 MHz, DMSO-*d*_6_): δ 9.55 (s, 1H, CH_pyrimdine_), 8.60 (s, 1H, CH_triazole_), 3.02–3.04 (m, 2H, CH_2cyhex_), 2.89–2.90 (m, 2H, CH_2cyhex_), 1.89–1.93 (m, 4H, 2 CH_2cyhex_); ^13^C NM (100 MHz, DMSO-*d*_6_): δ 155.09, 152.86, 148.26, 138.69, 137.48, 128.86, 120.14 (7 C_(1triazole+2pyrimdine+4thiophene)_), 25.28, 25.22, 22.89, 22.10 (4 CH_2cyhex_); CHN calcd. for: C_11_H_10_N_4_S [230.29]: C, 57.37; H, 4.38; N, 24.33; S, 13.92; found: C, 57.39; H, 4.40; N, 24.31; S, 13.90.

Synthesis of (**27**): A mixture of hydrazino derivative **21** (2.7 mmol) and acetyl acetone (2.8 mmol) in 20 mL abs. ethanol was refluxed for 6 h on cooling, a ppt is formed which was filtered and recrystallized from ethanol.

*4-(3,5-Dimethyl-1H-pyrazol-1-yl)-5,6,7,8-tetrahydrobenzo[4,5]thieno[2,3-d]pyrimidine (**27**)*

Yield: 67% yellow needles, m.p. 151–153 °C; ^1^H NMR (400 MHz, DMSO-*d*_6_): δ 8.97 (s, 1H, CH_pyrimidine_), 6.17 (s, 1H, CH_pyrazolyl_), 2.92 (brs, 2H, CH_2cyhex_), 2.21–2.30 (m, 8H, 2 C*H*_3_+C*H*_2cyhex_), 1.83–1.84 (m, 2H, CH_2cyhex_), 1.66 (brs, 2H, CH_2cyhex_); ^13^C NMR (100 MHz, DMSO-*d*_6_): δ 170.45, 152.00 (2 C_pyrimidine_), 151.77, 149.42, 141.85, 139.55, 127.50, 126.14, 107.85 (7 C_(3pyrazole+4thiophene)_), 25.95, 24.91, 22.58, 22.13 (4 CH_2cyhex_), 13.70, 11.80 (2*C*H_3_); CHN calcd. for: C_15_H_16_N_4_S [284.38]: C, 63.35; H, 5.67; N, 19.70; S, 11.28; found: C, 63.38; H, 5.65; N, 19.71; S, 11.26.

Synthesis of (**28**)

Method A: To the hydrazino derivative **21** (2.7 mmol) in 20 mL of ethanol containing aq. KOH (6.0 mmol) carbon disulfide (3.0 mmol) was added, the reaction mixture was refluxed for 6 h, then cooled, diluted with water, and acidified with concentrated HCl. A yellow ppt is formed, filtered off, washed with water, dried, and recrystallized from ethanol to give brown crystals. 

Method B: A mixture of hydrazino derivative **21** (1.8 mmol) and phenyl isothiocyanate (2.0 mmol) in 10 mL of ethanol was refluxed for 6 h. After that cooling, the solid product is formed, filtered off, and recrystallized from ethanol.

*8,9,10,11-Tetrahydrobenzo[4,5]thieno[3,2-e][1,2,4]triazolo[4,3-c]pyrimidine-3(2H)-thione (**28**)*

Yield: 85_A_%, 65_B_%, m.p. 283–285 °C; ^1^H NMR (400 MHz, DMSO-*d*_6_): δ 14.62 (s, 1H, D_2_O exchangeable, NH), 8.87 (s, 1H, CH_pyrimidine_), 2.90 (brs, 4H, 2 CH_2cyhex_), 1.88 (brs, 4H, 2 CH_2cyhex_); ^13^C NMR (100 MHz, DMSO-*d*_6_): δ 160.56, 151.28, 143.19 (3 C_(pyrimidine+C=S thioamide)_), 138.78, 135.69, 129.59, 117.23 (4 C_thiophene_ ), 25.14, 22.88, 22.05 (4 CH_2cyhex_); CHN calcd. for: C_11_H_10_N_4_S_2_ [262.35]: C, 50.36; H, 3.84; N, 21.36; S, 24.4; found: C, 50.32; H, 3.86; N, 21.32; S, 24.3.

The General Procedure for Synthesis of Compounds (**29**) and (**30**): The chloro derivative **20** (1.8 mmol) was refluxed in sodium methoxide or sodium ethoxide (prepared from 0.23 g of sodium in 30 mL of ethanol or methanol) for 6 h after finishing and the solvent was concentrated. The formed solid product was filtered off and recrystallized from the water-ethanol mixture.

*4-Methoxy-5,6,7,8-tetrahydrobenzo[4,5]thieno[2,3-d]pyrimidine (**29**)*

Yield: 71.8% colorless needle crystals, m.p. 98–100 °C; ^1^H NMR (400 MHz, DMSO-*d*_6_): δ 8.55 (s, 1H, CH_pyrimidine_), 4.04 (s, 3H, OC*H*_3_), 2.86 (brs, 2H, CH_2cyhex_), 2.80 (brs, 2H, CH_2cyhex_), 1.82–1.81 (m, 4H, 2 CH_2cyhex_); ^13^C NMR (100 MHz, DMSO-*d*_6_): δ 166.88, 163.91 (2 C_pyrimidine_), 152.67, 135.01, 127.49, 118.56 (4 C_thiophene_), 54.28 (O*C*H_3_), 25.91, 25.32, 22.83, 22.26 (4 CH_2cyhex_); CHN calcd. for: C_11_H_12_N_2_OS [220.29]: C, 59.97; H, 6.35; N, 12.60; S, 14.42; found: C, 59.99; H, 6.34; N, 12.61; S, 14.38.

*4-Ethoxy-5,6,7,8-tetrahydrobenzo[4,5]thieno[2,3-d]pyrimidine (**30**)*

Yield: 67% white solid, m.p. 109 °C; ^1^H NMR (400 MHz, DMSO-d_6_): δ 8.52 (s, 1H, CH_pyrimidine_), 4.52 (q, 2H, *CH_2_*CH_3_), 2.89–2.91 (m, 2H, CH_2cyhex_), 2.79–2.81 (m, 2H, CH_2cyhex_), 1.82–185 (m, 4H, 2 CH_2cyhex_), 1.40 (t, 3H, CH_2_*CH_3_*); ^13^C NMR (100MHz, DMSO-d_6_): δ 167, 163.58 (2 C_pyrimidine_), 152.70, 134.88, 127.59, 118.57 (4 C_thiophene_), 62.69 (O*CH_2_*), 25.86, 25.35, 22.86, 22.32 (4 C_cyhex_), 14.66 (CH_2_*CH_3_*); CHN calcd. for: C_12_H_14_N_2_OS [234.31]: C, 61.51; H, 6.02; N, 11.96; S, 13.68; found: C, 61.53; H, 6.05; N, 11.99; S, 13.66.

The General Procedure for Synthesis of Compounds (**31**–**35**): A mixture of chloro derivative **20** (2.0 mmol) and the appropriate amine (2.1 mmol) in 20 mL of ethanol was refluxed for 6 h. The solvent was concentrated and on cooling a solid product is formed which was filtered off, dried, and recrystallized from ethanol to afford the corresponding compound **31**–**35.**

*N-(p-Tolyl)-5,6,7,8-tetrahydrobenzo[4,5]thieno[2,3-d]pyrimidin-4-amine (**31**)*

Yield: 71%, brown plates crystals, m.p. 140–142 °C; ^1^H NMR (400 MHz, DMSO-*d*_6_): δ 8.34 (s, 1H, D_2_O exchangeable, NH), 7.98 (s, 1H, CH_pyrimidine_), 7.55 (d, *J* 8.0 Hz, 2H, 2HAr), 7.17 (d, *J* 8 Hz, 2H, 2CH, 2HAr), 3.12 (brs, 2H, CH**_2_**_cyhex_), 2.82 (brs, 2H, CH_2cyhex_), 2.30 (s, 3H, CH_3_), 1.86 (brs, 4H, 2 CH**_2_**_cyhex_); ^13^C NMR (100 MHz, DMSO-*d*_6_): δ 166.06, 155.51 (2 C_pyrimidine_), 152.55, 137.02, 133.20, 132.96, 129.29, 127.01, 122.83, 117.02 (10 C_(4thiophene+6phenyl)_), 25.89, 25.52, 22.61, 22.48 (4 CH_2cyhex_), 20.93 (CH_3_); CHN calcd. for: C_17_H_17_N_3_S [295.40]: C, 69.12; H, 5.80; N, 14.22; S, 10.85; found: C, 69.10; H, 5.81; N, 14.25; S, 10.79.

*N-Benzyl-5,6,7,8-tetrahydrobenzo[4,5]thieno[2,3-d]pyrimidin-4-amine (**32**)*

Yield: 65.5%, m.p. 162–164 °C; ^1^H NMR (400 MHz, DMSO-d*_6_*): δ 8.54 (d, 1H, *J* 8.0 Hz, phenyl), 8.43 (s, 1H, CH_pyrimidine_), 8.13 (s, 1H, D_2_O exchangeable, NH), 6.98–7.11 (m, 4H, phenyl), 3.92 (s, 2H, NH*CH_2_*Ph), 3.07–3.09 (m, 2H, CH_2cyhex_), 2.82–2.84 (m, 2H, CH_2cyhex_), 1.87–1.94 (m, 4H, 2 CH_2cyhex_); ^13^C NMR (100 MHz, DMSO-d*_6_*): δ 165.60, 154.84 (2 C_pyrimidine_), 152.71, 149.38, 134.05, 128.79, 126.24, 123.66, 121.03, 117.15, 111.42 (10 C_(6phenyl+4thiophene)_), 56.77 (CH2-NHBn), 25.94, 25.49, 22.58, 22.40 (4 CH_2cyhex_); CHN calcd. for: C_17_H_17_N_3_S [295.40]: C, 69.12; H, 5.80; N, 14.22; S, 10.85; found: C, 69.10; H, 5.81; N, 14.25; S, 10.79.

*4-((5,6,7,8-Tetrahydrobenzo[4,5]thieno[2,3-d]pyrimidin-4-yl)amino)benzoic acid (**33**)*

Yield: 88% yellow crystals, m.p. 269–271 °C; ^1^H NMR (400 MHz, DMSO-*d*_6_): 8.50 (s, 2H, CH_pyrimidine_+COOH), 7.93 (d, 2H, *J* 8.0 HZ, 2HAr), 7.82 (d, 2H, *J* 8.0 Hz, 2HAr), 5.09 (s, 1H, D_2_O exchangeable, NH), 3.16 (brs, 2H, CH_2cyhex_), 2.86 (brs, 2H, CH_2cyhex_), 1.92 (brs, 4H, 2 CH_2cyhex_); ^13^C NMR (100 MHz, DMSO-*d*_6_): δ 167.43 (C=O), 165.69, 154.72 (2 C_pyrimidine_), 151.97, 143.99, 134.49, 130.50, 127.13, 125.30, 120.89, 118.14 (10 C_(4thiophene+ 6phenyl)_), 25.71, 25.59, 22.60, 22.40 (4 CH_2cyhex_); CHN calcd. for: C_17_H_15_N_3_O_2_S [325.39]: C, 62.75; H, 4.65; N, 12.91; S, 9.85; found: C, 62.80; H, 4.60; N, 12.93; S, 9.87.

*1-(4-((5,6,7,8-Tetrahydrobenzo[4,5]thieno[2,3-d]pyrimidin-4-yl)amino)phenyl)ethan-1-one (**34**)*

Yield: 67% brown crystals, m.p. 173–175 °C; ^1^H NMR (400 MHz, DMSO-d_6_): δ 8.49 (s, 1H, D_2_O exchangeable, NH), 8.38 (s, 1H, CH_pyrimidine_), 7.94 (d, 2H, *J* 8.0 Hz, 2HAr), 7.83 (d, 2H, *J* 8.0 Hz, 2HAr), 3.14 (brs, 2H, CH_2cyhex_), 2.84 (brs, 2H, CH_2cyhex_), 2.54 (s, 3H, OC*H_3_*), 1.87 (brs, 4H, 2 CH_2cyhex_); ^13^C NMR (100 MHz, DMSO-d_6_): δ 196.81 (C=O), 166.86, 154.51 (2 C_pyrimidine_), 152.27, 144.48, 134.42, 131.65, 129.55, 126.94, 120.42, 118.17 (10 C_(6phenyl+4thiophene)_), 26.81 (CH_3_), 25.7, 25.59, 22.60, 22.42 (4 CH_2cyhex_); CHN calcd. for: C_18_H_17_N_3_OS [323.41]: C, 66.85; H, 5.30; N, 12.99; S, 9.91; found: C, 66.82; H, 5.31; N, 12.96; S, 9.90.

*1,2,3,4-Tetrahydro-9H-benzo[4’,5’]thieno[2’,3’:4,5]pyrimido[6,1-b]quinazolin-9-one (**35**)*

Yield: 70% yellow crystals, m.p. 285 °C; ^1^H NMR (400 MHz, DMSO-*d*_6_): δ 9.34 (s, 1H, CH_pyrimdine_), 8.35 (d, 1 H_phenyl_), 7.96 (t, 1 H_phenyl_), 7.78 (d, 1 H_phenyl_), 7.57 (t, 1 H_phenyl_) 2.91, 1.90 (4 CH_2cyhex_); CHN calcd. for: C_17_H_13_N_3_OS [307.37]: C, 66.43; H, 4.26; N, 13.67; S, 10.43; found: C, 66.40; H, 4.24; N, 13.69; S, 10.40.

### 3.2. Biological Experimental Assays (Full Protocols, see Supplementary Materials)

#### 3.2.1. In Vitro

In vitro work regarding cell culturing, cytotoxic screening using the MTT assay, and the IC_50_ calculations were made according to Mosmann 1983 (see Supplementary Materials) [38].

#### 3.2.2. Flow Cytometry

All flow cytometry including FITC/Annexin-V-FITC/PI differential apoptosis/necrosis assessment, DNA content-flow cytometry aided cell cycle analysis, and acridine orange quantitative autophagy assessment were made according to Nafie et al., 2020 [39].

#### 3.2.3. In Silico Molecular Docking

All in silico studies including ligand optimization, protein preparation, and molecular docking calculation were investigated followed by the reported [40].

#### 3.2.4. Bioinformatics

Bioinformatics study (in silico and bioactivity prediction) of the most active compounds were calculated using a set of software’s including MolSoft, Molinspiration [41], and SwissADME [42] websites as previously described by Youssef et al., 2020 [37].

#### 3.2.5. In Vivo (SEC) Model

**Figure 7:**
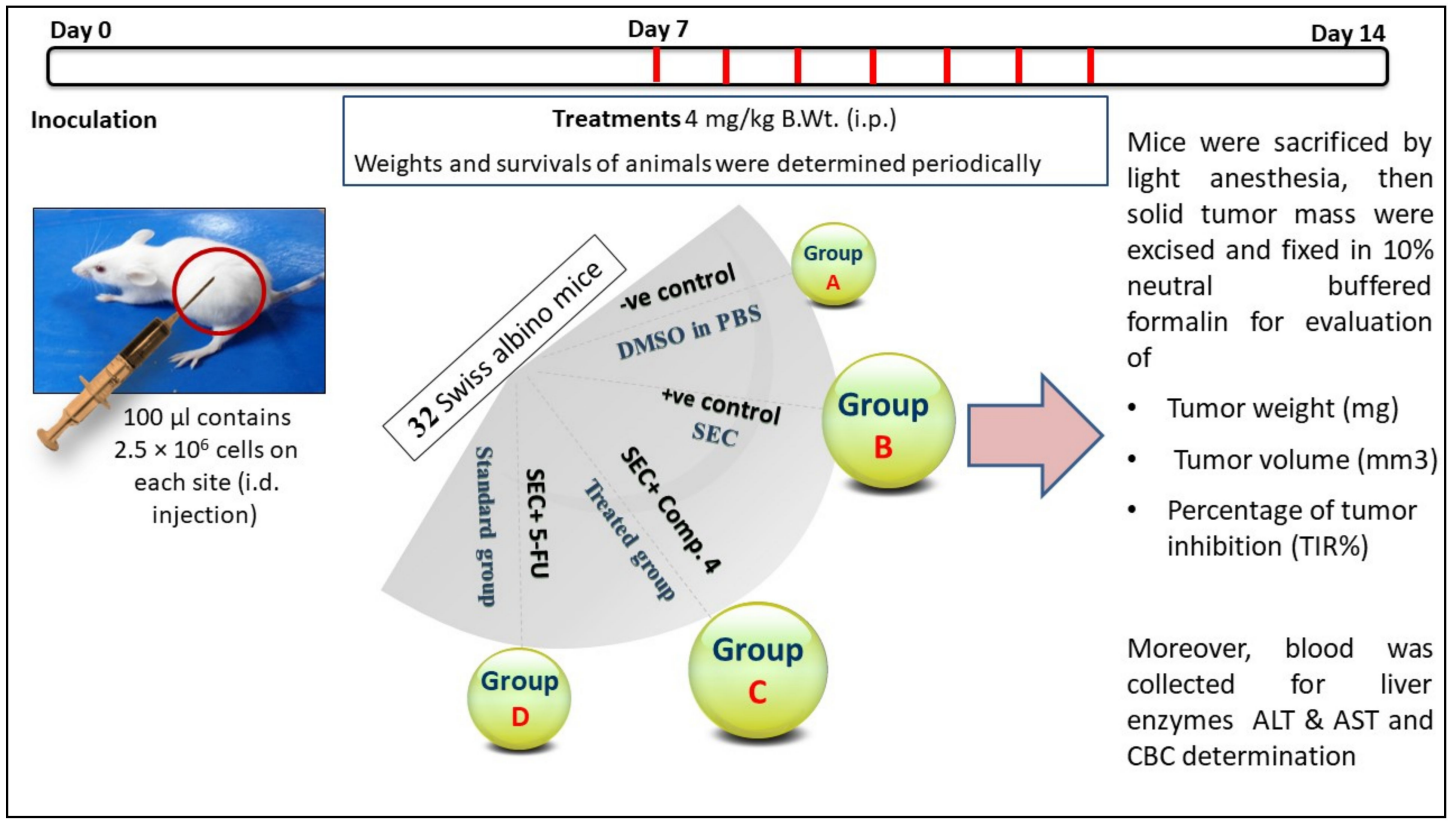
Summarized methodology of Solid Ehrlich Carcinoma (SEC)-bearing mice in vivo.

Experiment design and methodology including tumor volume and percentage of tumor inhibition were summarized in Figure 7.

#### 3.2.6. Statistical Analysis

Data were represented by the value of the mean for three different replicates, with the standard error of the mean (mean ± SEM). All statistical analysis was done as using GraphPad Prism software version 7.0. The statistical difference between two groups was examined using the unpaired *t*-test. The significance level was set at *p* < 0.05.

## 4. Conclusions

In conclusion, approximately 33 compounds were synthesized and characterized. The target compounds were evaluated in vitro against HepG-2 and MCF-7 cancer cell lines. Hit **4** was found to be the most active, and the cell cycle analysis showed that this lead induces apoptosis. Moreover, the in vivo study demonstrates that our target significantly reduces the tumor mass. The in silico molecular docking shows the binding of **4** as an agonist for JAK2 inhibitors. 

## Figures and Tables

**Table 1:** The in vitro inhibition % found using (100 µg/mL) of a single dose and IC_50_ in µg/mL and µM of the tested compounds on MCF-7 and HepG-2 cell lines.

Entry	MCF-7			HepG-2		
	Inhibition %	IC_50_ µg/mL	IC_50_ µM	Inhibition %	IC_50_ µg/mL	IC_50_ µM
2	27.12551	ND	ND	42.85714	ND	ND
3	46.93572	ND	ND	43.42105	ND	ND
4	80.625	7	23.2	78.63636	11.7	38.8
5	75.91912	19.3	52.9	76.41221	19.3	52.9
6	38.56502	ND	ND	45.17544	ND	ND
7	74.77941	48	131.3	73.58779	37	101.2
8	49.0284	ND	ND	52.41228	ND	ND
9	45.06726	ND	ND	81.79825	36.5	140.7
10	67.70833	ND	ND	78.18182	26.5	89.4
11	45.29148	ND	ND	72.98246	16.5	50.9
12	38.93871	ND	ND	73.07018	31	87.5
13	56.65172	ND	ND	66.22807	27.5	94.0
14	61.13602	43	154.5	69.34211	33.6	120.7
15	60.46338	22	53.2	73.24561	22	53.2
16	66.66667	ND	ND	77.09091	25	68.2
17	67.70833	ND	ND	79.09091	42	110.4
19	68.41912	ND	ND	77.17557	38.5	334.42
20	34.90284	ND	ND	34.21053	ND	ND
21	60.38864	28	127.1	69.73684	13.6	61.7
22	31.98381	ND	ND	30.61224	ND	ND
23	1.00005	ND	ND	3.508772	ND	ND
24	72.39583	13	40.3	78.63636	10	31.0
25	38.7145	ND	ND	29.82456	ND	ND
26	70.67708	16.4	71.2	77.27273	29	125.9
27	79.0625	21.7	76.3	77.72727	32.6	114.6
28	70.40359	15.5	59.1	47.36842	ND	ND
29	80.20833	11	49.9	76.36364	14.5	65.8
30	72.91667	7.4	31.6	78.72727	8.8	37.6
31	76.04167	9.8	33.2	80.45455	17	57.5
32	47.36842	ND	ND	51.83673	ND	ND
33	71.875	31.2	95.9	81.36364	40	122.9
34	57.8125	ND	ND	77.63636	ND	ND

ND: Not determined.

**Table 2:** Analysis of ligand-receptor interactions with binding energies of docked compound **4** inside three proteins **3ZMM**, **4C62**, and **5AEP** as Jak2 inhibitors.

Protein (PDB Code)	Binding Affinity (Kcal/mol)	Type of Interaction	Bond Length (Aº)	Interaction Moiety Involved	Amino Acid
**3ZMM**	**–14.32**	**H-acceptor**	**1.97**	**C=O**	**Leu 932**
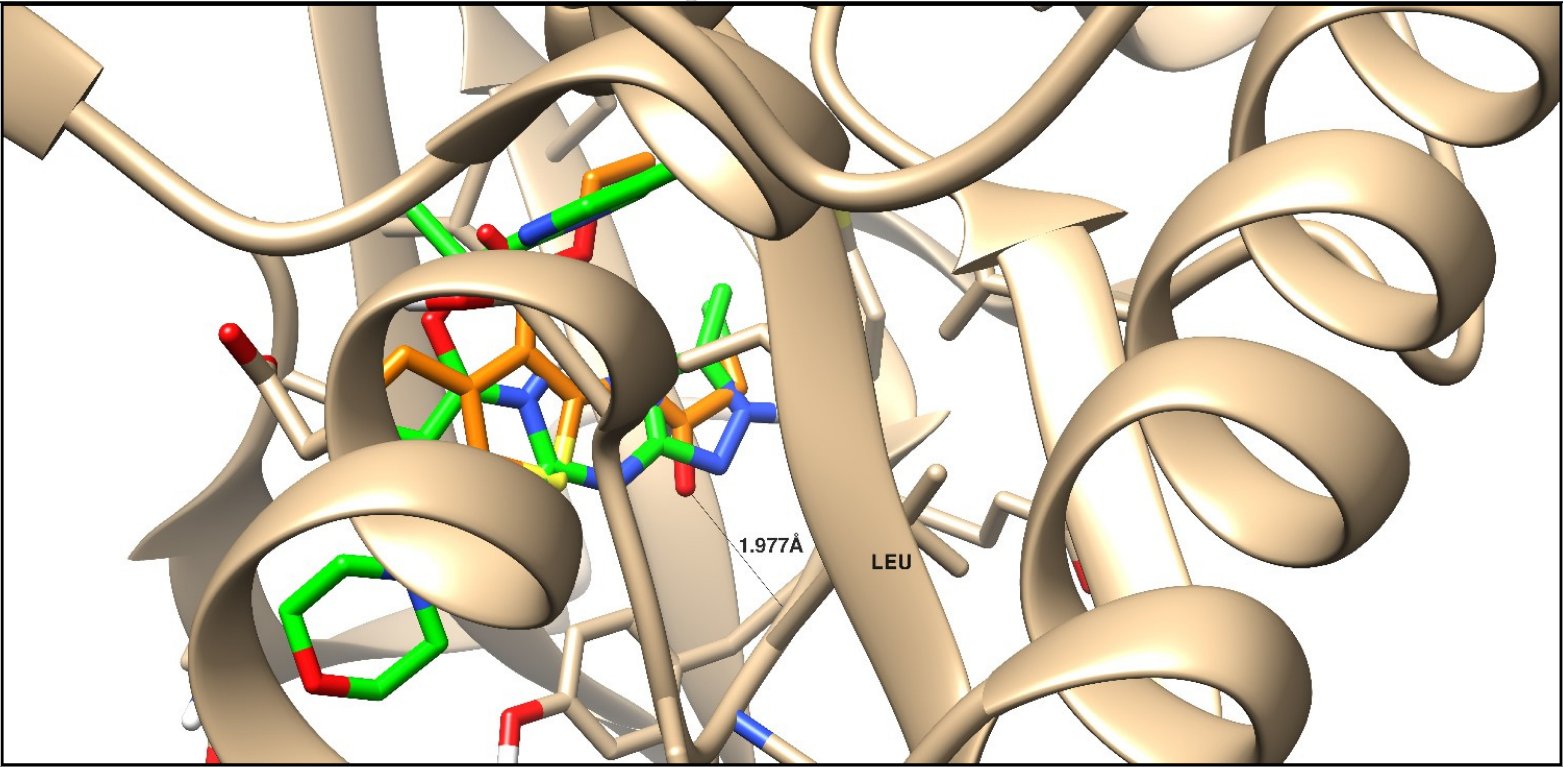					
**4C62**	**–13.39**	H-acceptor	1.84	**C=O**	**Leu 932**
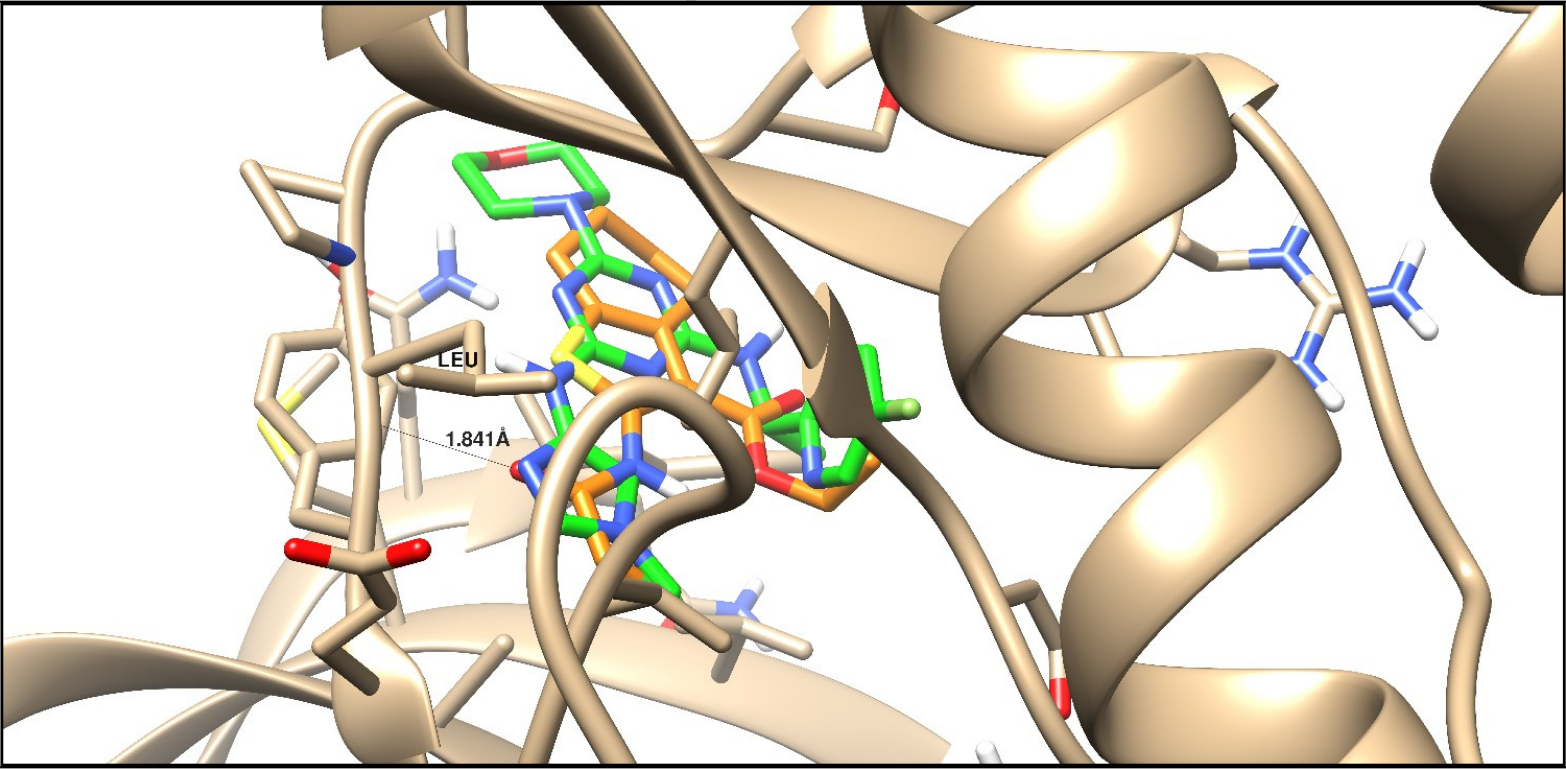					
**5AEP**	**–11.38**	H-acceptor	1.67	**C=O**	**Leu 932**
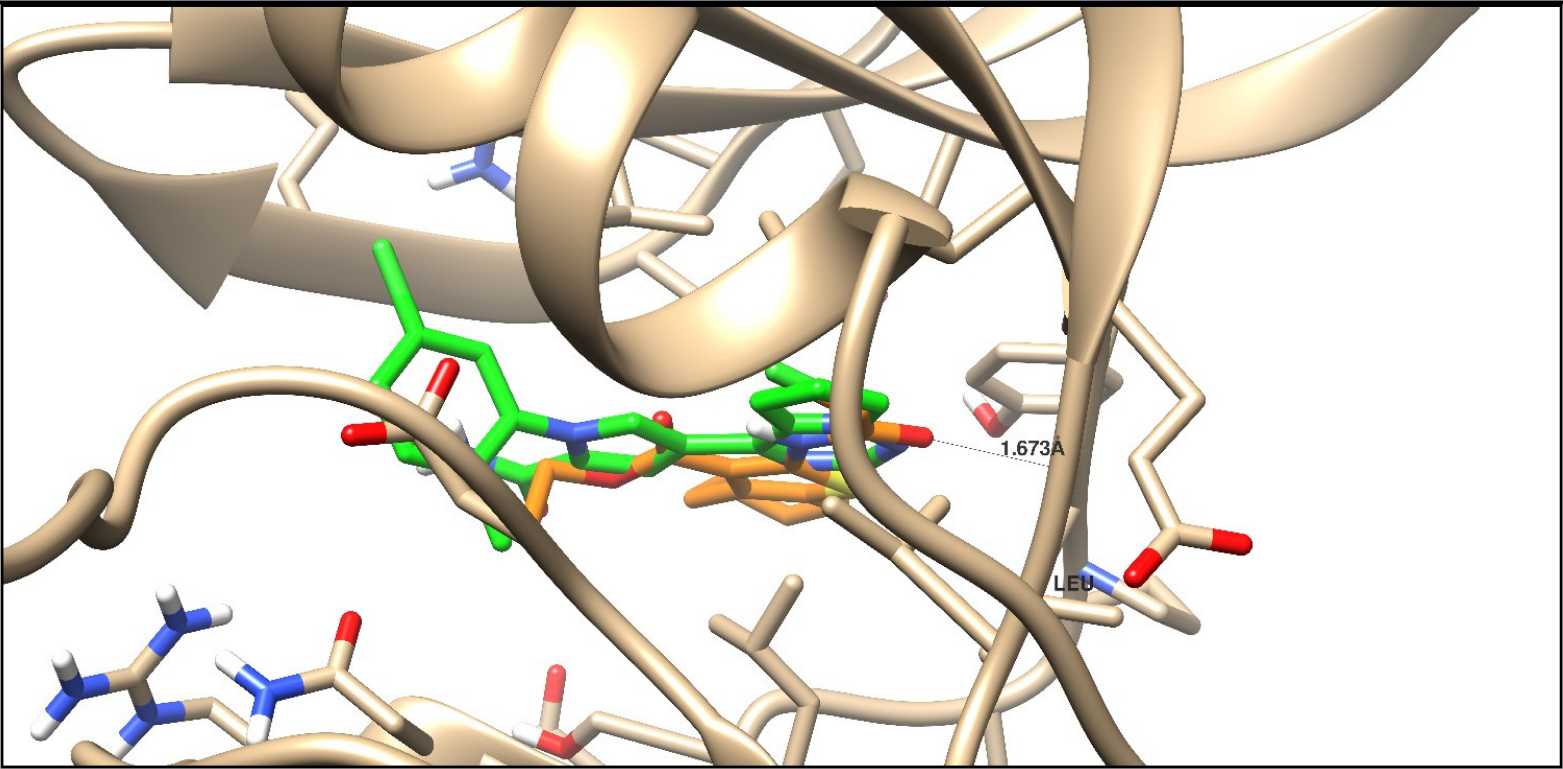					

Superimposed compound **4** (orange), and the co-crystalized ligand (green) of the three studied **3ZMM**, **4C62,** and **5AEP** proteins.

**Table 3:** In silico ADME pharmacokinetics properties.

#	Molinspiration 2018.10						MolSoft			
	MWt (D)	MV (A^3^)	PSA (A^2^)	Log *P*	nrotb	nviolations	HBA	HBD	Solubility (mg/L)	Drug-Likeness Score
**4**	301.79	254.18	55.40	2.82	5	0	4	1	187.99	0.48
**31**	295.40	267.17	37.81	4.80	2	0	3	1	2.51	0.48
**24**	322.43	290.43	50.17	4.33	3	0	4	1	2.13	0.73
**30**	234.31	209.15	35.02	3.03	2	0	4	0	14.58	0.15

“Mwt: Molecular Weight, MV: Molecular Volume, PAS: Polar Surface Area, Log *P*: Octanol-water partition coefficient, nrotb: number of rotatable bond, nviolations: number of violations, HBA: Hydrogen Bond Acceptor, HBD: Hydrogen Bond Donor” [37].

**Table 4:** Evaluation of liver enzymes and hematological parameters in the studied groups.

Treatments	Positive Control	SEC Control	SEC + Comp 4	SEC + 5-FU
ALT *^#^ (I/U)	43.53 ± 1.06	84.65 ± 2.1	64.28 ± 1.64	51.31 ± 1.41
AST *^#^ (I/U)	45.75 ± 0.97	97.64 ± 1.75	59.89 ± 1.85	50.64 ± 0.97
Hb *^#^ (g/dL)	7.9 ± 0.86	3.99 ± 0.21	5.99 ± 0.49	7.02 ± 0.83
RBCs count *^#^ (× 10^6^/μL)	5.14 ± 0.65	3.28 ± 0.73	4.43 ± 0.47	4.98 ± 021
WBCs count *^#^ (× 10^3^/μL)	3.69 ± 0.53	4.89 ± 0.49	4.21 ± 0.43	3.98 ± 0.32

* Mean ± SEM values of mice in each group (n = 8). ^#^ Values are significantly different (*p* ≤ 0.05), un-paired test using GraphPad prism.

## Supplementary Materials

The following are available online, Full protocols for the biological assays; Figure S1–S87 copies of the HNMR and CNMR spectrum of the synthesized compounds.
