# Supplementary material for: Discovery of New Apoptosis-Inducing Agents for Breast Cancer Based on Ethyl 2-Amino-4,5,6,7-Tetra Hydrobenzo[b]Thiophene-3-Carboxylate: Synthesis, In Vitro, and In Vivo Activity Evaluation
Source: Molecules. 2020 May 28;25(11):2523. doi: 10.3390/molecules25112523 (PMC7321303; doi:10.3390/molecules25112523)
Supplement: Supplementary file 1 [file molecules-25-02523-s001.pdf]

SUPPORTING INFORMATION

Discovery of New Apoptosis-Inducing Agents for Breast Cancer Based on Ethyl 2-Amino-4,5,6,7-Tetra Hydrobenzo[*b*]Thiophene-3-Carboxylate: Synthesis, In Vitro, and In Vivo Activity Evaluation

Emad M. Gad [1,*], Mohamed S. Nafie [1], Elsayed H Eltamany [1], Magdy S. A. G. Hammad [1], Assem Barakat [2,3] and Ahmed T. A. Boraei [1,*]

[1] Chemistry Department, Faculty of Science, Suez Canal University, Ismailia 41522, Egypt. Emails: Mohamed_nafie@science.suez.edu.eg (M.S.N.); s.eltamany51@yahoo.com (E-S.H.E-T.); magdy_hammad83@yahoo.com (M.G.H).

[2] Chemistry Department, College of Science, King Saud University, P.O. Box 2455, Riyadh 11451, Saudi Arabia. Email: ambarakat@ksu.edu.sa (A.B.)

[3] Chemistry Department, Faculty of Science, Alexandria University, P.O. Box 426, Ibrahimia, Alexandria 21321, Egypt

* Correspondence: emad_gad@science.suez.edu.eg (E.M.G), ahmed_tawfeek83@yahoo.com (A.T.A.B.)

Experimental

Melting points were determined on a Temp-melt II melting-point apparatus, and the values are uncorrected. TLC was carried out on silica gel 60 F254 Aluminum plates (E. Merck, layer thickness 0.2 mm), and purity detection was performed using UV lamp. ^{1}H NMR and ^{13}C NMR spectra were recorded on a Bruker NMR instrument operating at 400 and 100 MHz, respectively. Chemical shifts were reported in parts per million (ppm). CHNS-microanalysis was performed on a Flash EA-1112 instrument.

Anti-tumor Activity

In vitro antitumor activity:

Potential cytotoxicity of the newly synthesized compounds was tested against breast (MCF-7) and hepatocellular carcinoma cell line (HepG2) using the method of Mosmann [1]. The *in vitro* anticancer screening was done by pharmacology unit in cancer biology department at the National Cancer Institute, Cairo University, Cairo, Egypt. Cells were plated in 96-multiwell plate (104 cells/well) for 24 h. Different concentrations of the compounds under test (0, 5, 12.5, 25, and 50 μg/mL) were added to the cell monolayer triplicate wells were prepared for each dose. Monolayer cells were incubated with the compounds for 48 h at 37 °C and in atmosphere of 5% CO_2. After 48 hrs, cells were fixed, washed, and stained with Sulfo-Rhodamine-B stain.

Excess stained was washed with acetic acid, and the attached stained was recovered with Tris EDTA buffer. Colour intensity was measured in an ELISA reader. The relation between surviving fraction and compound concentration is plotted to get the survival curve of the tumor cell line after the specified compound.

FITC/Annexin -V-FITC/PI differential apoptosis/necrosis assessment

Apoptosis and necrosis cell populations are determined using Annexin V-FITC apoptosis detection kit (Abcam Inc., Cambridge Science Park, Cambridge, UK) coupled with 2 fluorescent channels flowcytometry. After treatment with test compounds for 48 h, cells (10^5 cells) are collected by trypsinization and washed twice with ice-cold PBS (pH 7.4). Then, cells are incubated in dark with 0.5 mL of Annexin V-FITC/PI solution for 30 min in dark at room temperature according to manufacturer protocol. After staining, cells are injected via ACEA Novocyte™ flowcytometer (ACEA Biosciences Inc., San Diego, CA, USA) and analyzed for FITC and PI fluorescent signals using FL1 and FL2 signal detector, respectively (λex/em 488/530 nm for FITC and λex/em 535/617 nm for PI). For each sample, 12,000 events are acquired and positive FITC and/or PI cells are quantified by quadrant analysis and calculated using ACEA NovoExpress™ software (ACEA Biosciences Inc., San Diego, CA, USA).

DNA content-flowcytometry aided cell cycle analysis

After treatment with test compounds for 48 h, cells (105 cells) are collected by trypsinization and washed twice with ice-cold PBS (pH 7.4). Cells are re-suspended in two milliliters of 60% ice-cold ethanol and incubated at 4 °C for 1 h for fixation. Fixed cells are washed twice again with PBS (pH 7.4) and re-suspended in 1 mL of PBS containing 50 μg/mL RNAase A and 10 μg/mL propidium iodide (PI). After 20 min of incubation in dark at 37 °C, cells are analyzed for DNA contents using flow cytometry analysis using FL2 (λex/em 535/617 nm) signal detector (ACEA Novocyte™ flowcytometer, ACEA Biosciences Inc., San Diego, CA, USA). For each sample, 12,000 events are acquired. Cell cycle distribution is calculated using ACEA NovoExpress™ software (ACEA Biosciences Inc., San Diego, CA, USA).

Acridine Orange quantitative autophagy assessment

Autophagic cell death is quantitatively assessed using acridine orange lysosomal stain coupled with flowcytometric analysis. After treatment with test compounds for 48h, cells (10^5 cells) are collected by trypsinization and washed twice with ice-cold PBS (pH 7.4). Cells are stained with acridine orange (10 μM) and incubated in dark at 37 °C for 30 minutes. After staining, cells are injected via ACEA Novocyte™

flowcytometer (ACEA Biosciences Inc., San Diego, CA, USA) and acridine orange fluorescent signals is analyzed using FL1 signal detector (λex/em 488/530 nm). For each sample, 12,000 events are acquired and net fluorescent intensities (NFI) are quantified using ACEA NovoExpress™ software (ACEA Biosciences Inc., San Diego, CA, USA).

Molecular docking studies

All molecular modeling studies were conducted on a computational software basis using the Molecular Operating Environment (MOE 2008-10 Chemical Computing Group, Canada) towards three proteins as Jak2 inhibitors; 3ZMM, 4C62, and 5AEP whose crystal structures complexed with their co-crystallized ligands were easily accessible from the Protein Data bank. Principles of modeling regarding receptor and ligand preparation and molecular docking were carried out according to Nafie *et al.* [2]. Each ligand-receptor complex was tested for interaction analysis, 2D images were made using the MOE visualizing tool, and 3D images were taken by Chimera as a visualizing software.

I*n vivo* evaluation

Swiss albino mice were purchased from the laboratory animal house of Misr International University. Ehrlich carcinoma cells were purchased from the National Cancer Institute, Cairo, Egypt. *In vivo* animal experiments were conducted in compliance with the National Institutes of health guide for the care and use of laboratory animals. Both Experiment design and methodology, including; tumor volume and percentage of tumor inhibition are, according to Gaballah *et al.* 2017 [3], are summarized in Figure (7). Moreover, handling of blood samples and determination of complete blood count and liver enzymes. CBC was estimated using Abbott CELL-DYN® 1800 automated hematology, while liver enzymes were measured in serum using commercial kits (Instrumentation Laboratory SpA, Inova diagnostics, Milano, Italy) according to Nafie *et al.* 2020 [4].

References

1. Mosmann, T. Rapid colorimetric assay for cellular growth and survival: Application to proliferation and cytotoxicity assays. *J. Immunol. Methods.* **1983**, *65*, 55–63.
2. Nafie, M.S.; Tantawy, M.A.; Elmgeed, G.A. Screening of different drug design tools to predict the mode of action of steroidal derivatives as anti-cancer agents. *Steroids* **2019**, *152*, 108485. DOI: 10.1016/j.steroids.2019.108485.
3. Gaballah, H.H.; Gaber, R.A.; Mohamed, D.A. Apigenin potentiates the antitumor activity of 5-FU on solid Ehrlich carcinoma: Crosstalk between apoptotic and JNK-mediated autophagic cell death platforms, *Toxicol. Appl. Pharmacol.* **2017**, *316*, 27–35. doi: 10.1016/j.taap.2016.12.012.
4. Nafie, M.S.; Abdel Daim, M.M.; Ali, I.A.I.; Nabil, Z.I.; Tantawy, M.A.; Abdel-Rahman, M.A. Antitumor efficacy of the Egyptian Scorpion Venom Androctonus Australis: in vitro and in vivo study, *J. Basic Appl. Zool.* **2020,** *81*. doi.org/10.1186/s41936-020-00147-1.

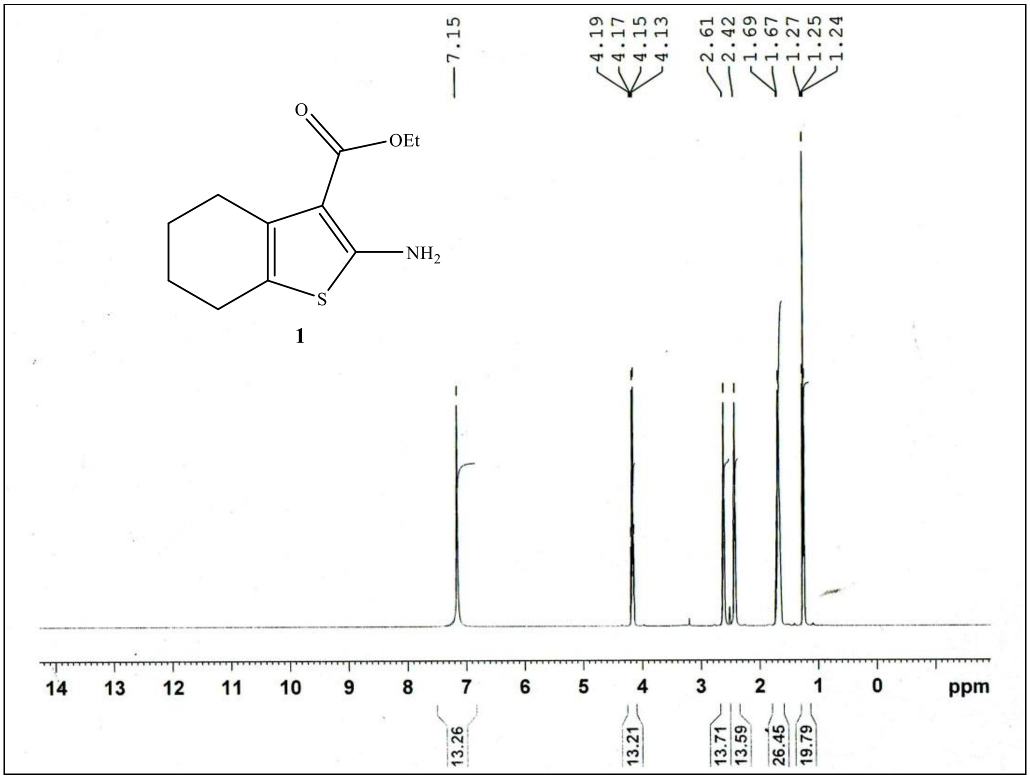

FigS2. ^{1}H NMR of **1** DMSO-d_6

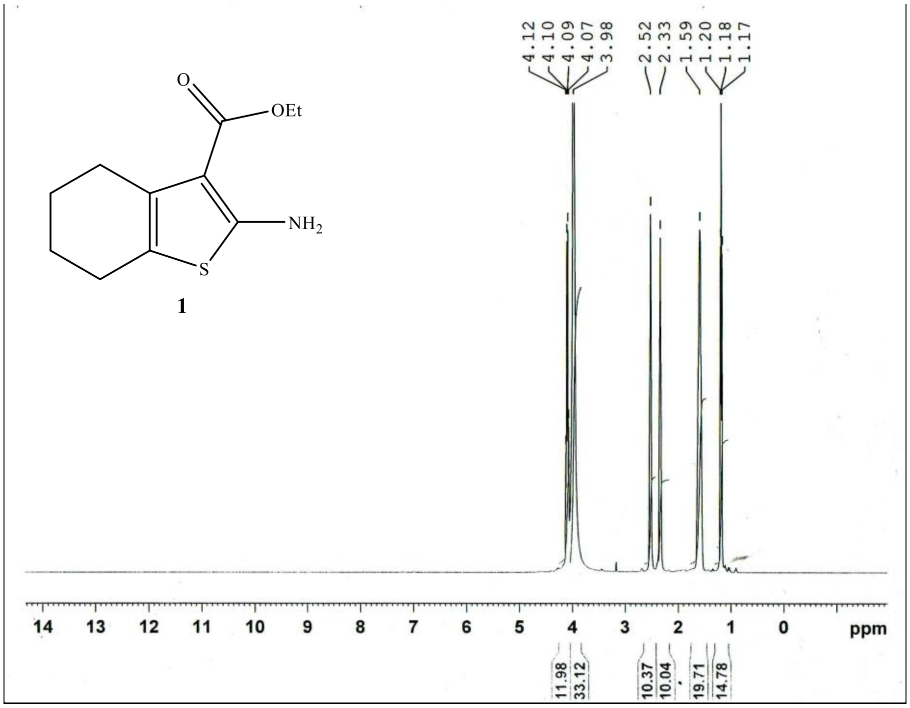

FigS3. ^{1}H NMR of **1** DMSO-d_6 + D_2O

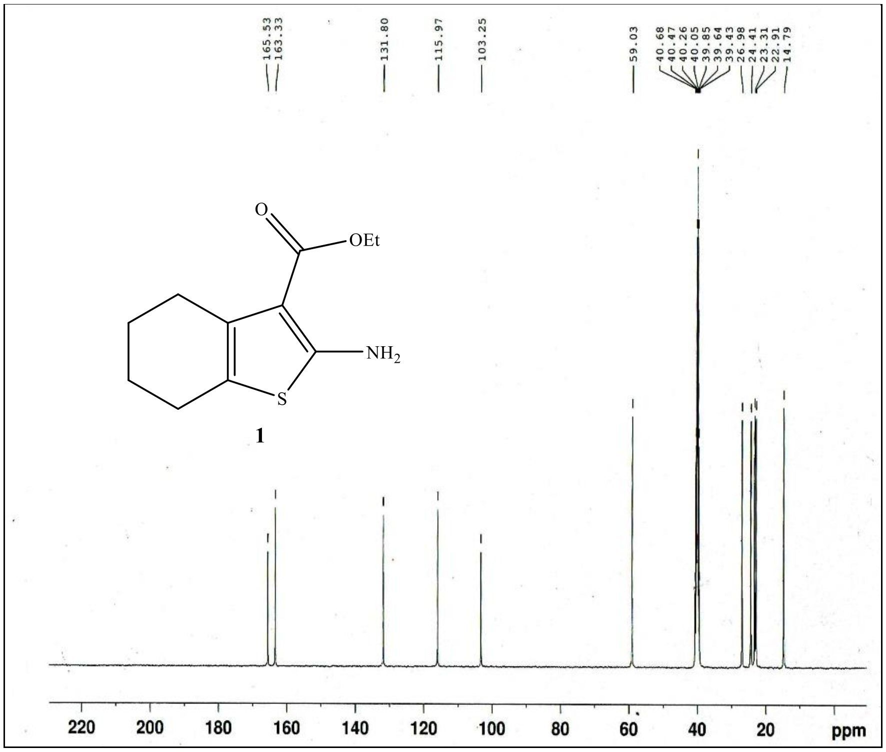

Fig. S4. ^{13}C NMR of **1** DMSO-d_6

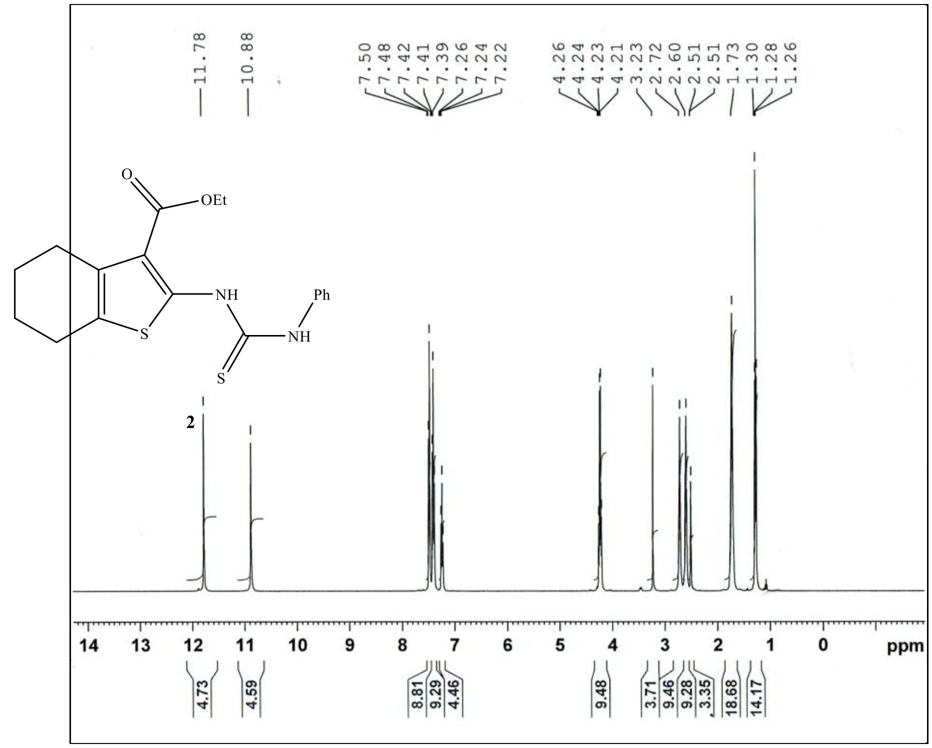

Fig. S5. ^{1}H NMR of **2 +** DMSO-d_6

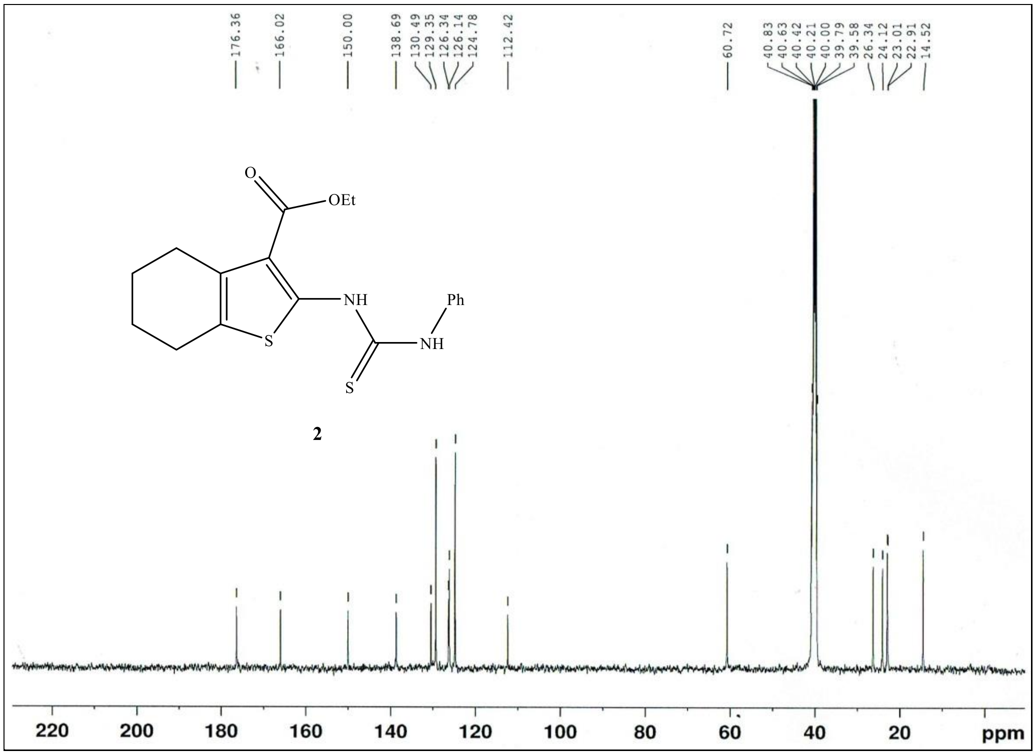

Fig. S6. ^{13}C NMR of **2** + DMSO-d_6

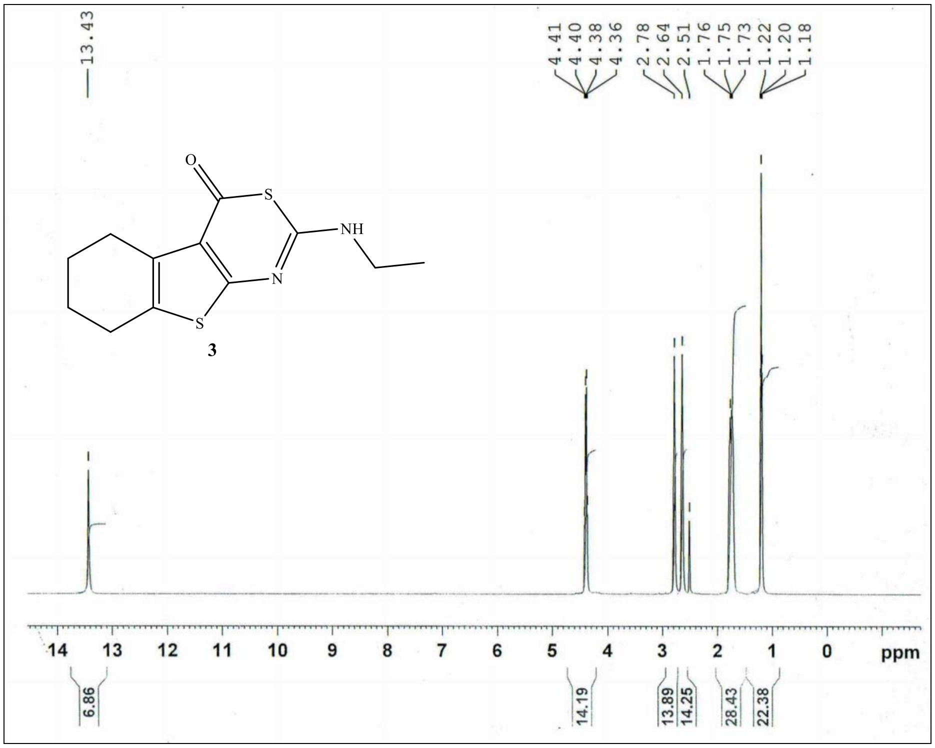

Fig. S7. ^{1}H NMR of **3 +** DMSO-d_6

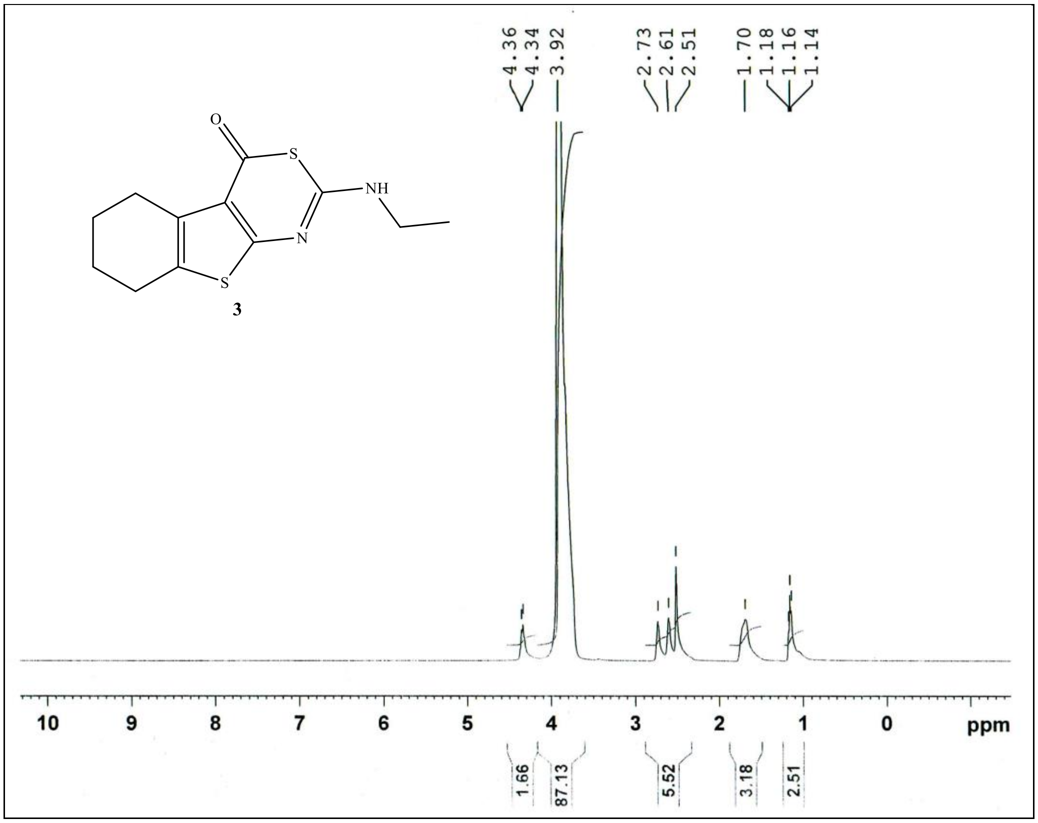

Fig. S8. ^{1}H NMR of **3** in DMSO-d6 + D_2O

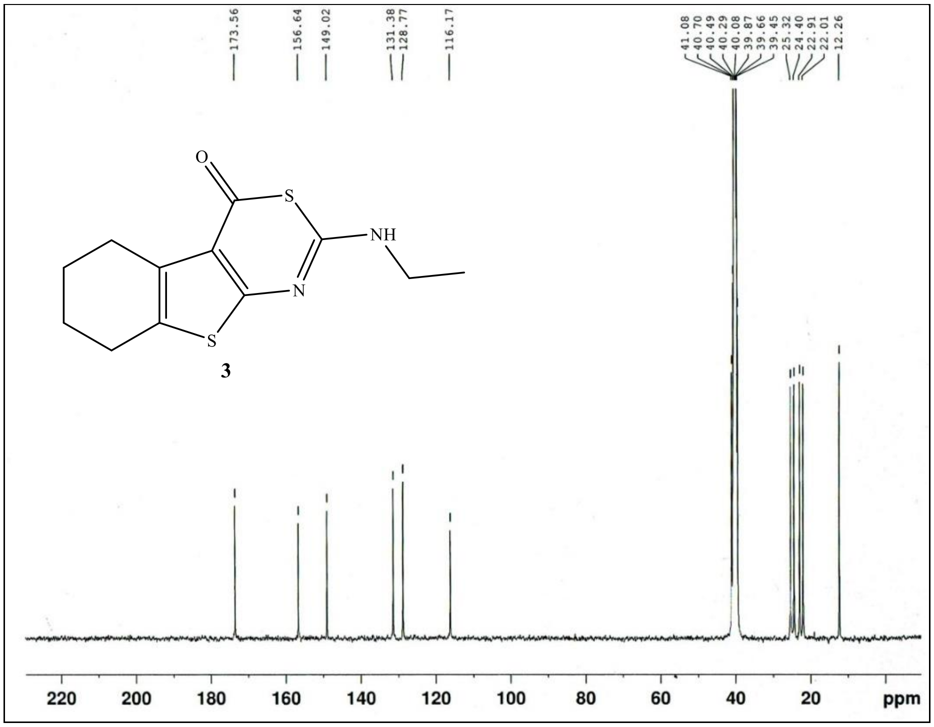

Fig. S9. ^{13}C NMR of **3** + DMSO-d_6

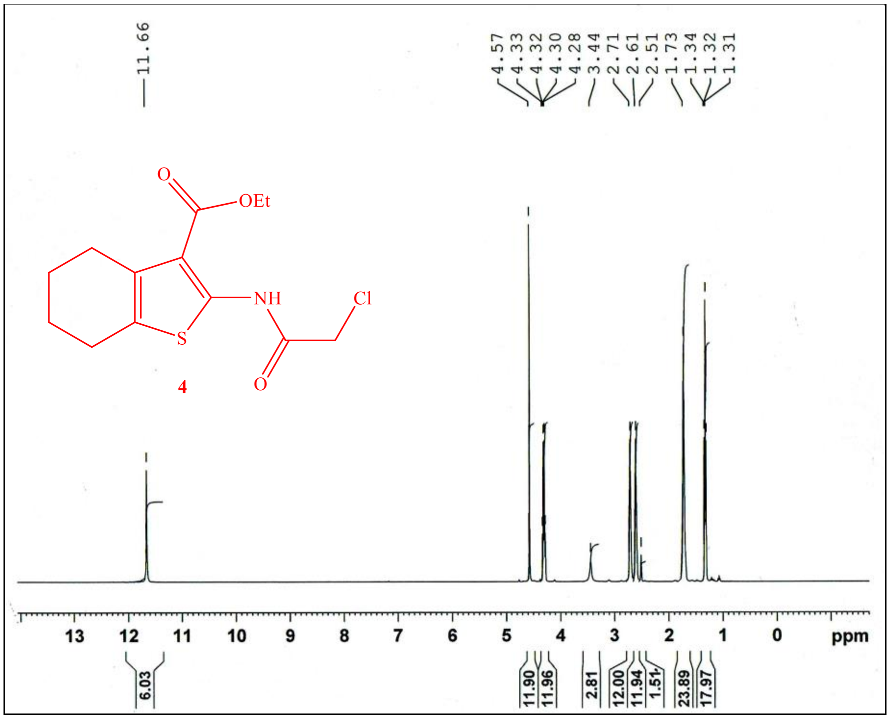

Fig. S10. ^{1}H NMR of **4** + DMSO-d_6

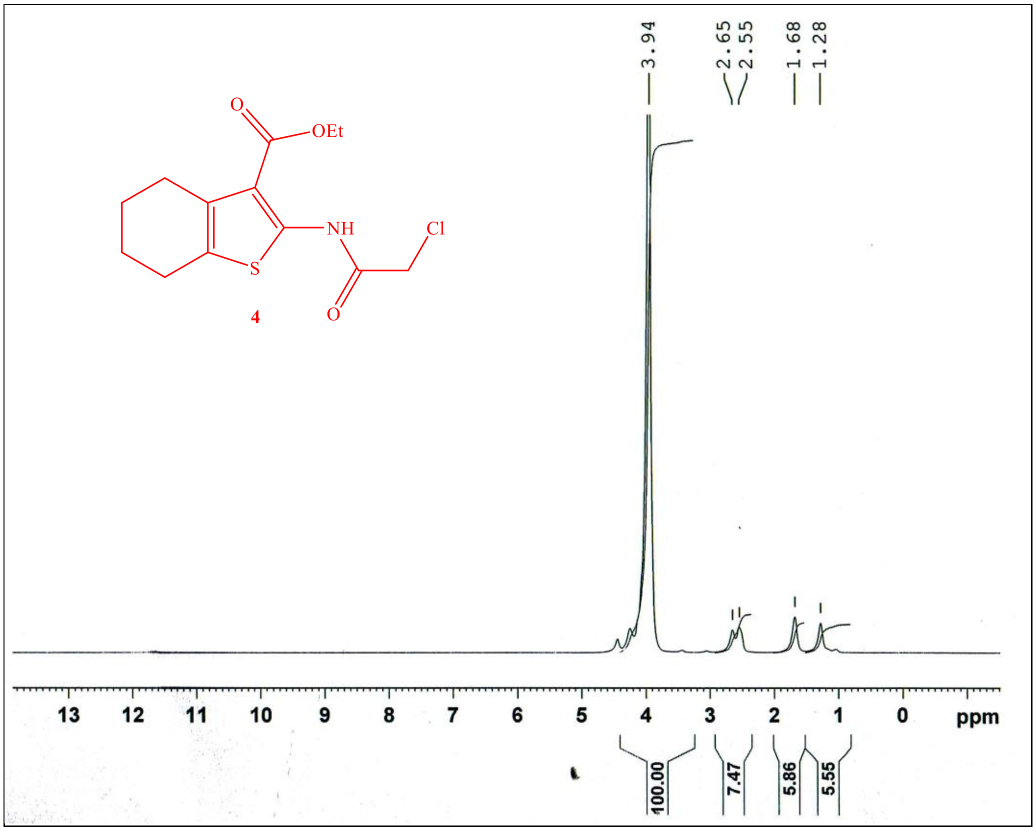

Fig. S11. ^{1}H NMR of **4** in DMSO-d_6 + D_2O

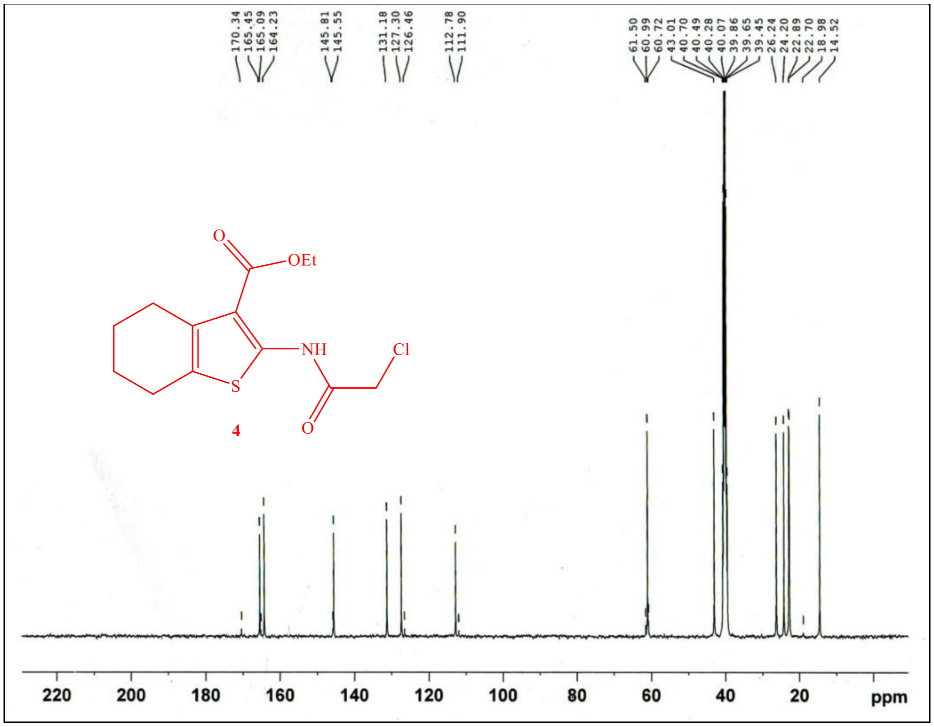

Fig. S12. ^{13}C NMR of **4** in DMSO-d_6

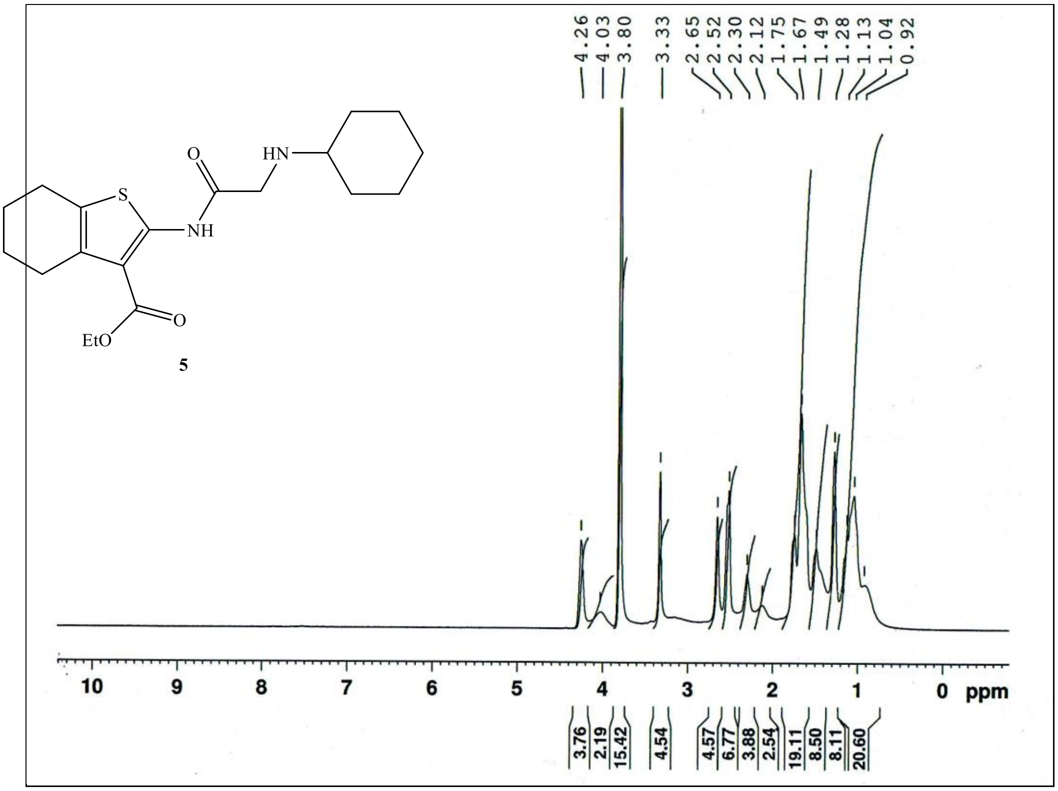

Fig. S13. ^{1}H NMR of **5** in DMSO-d_6

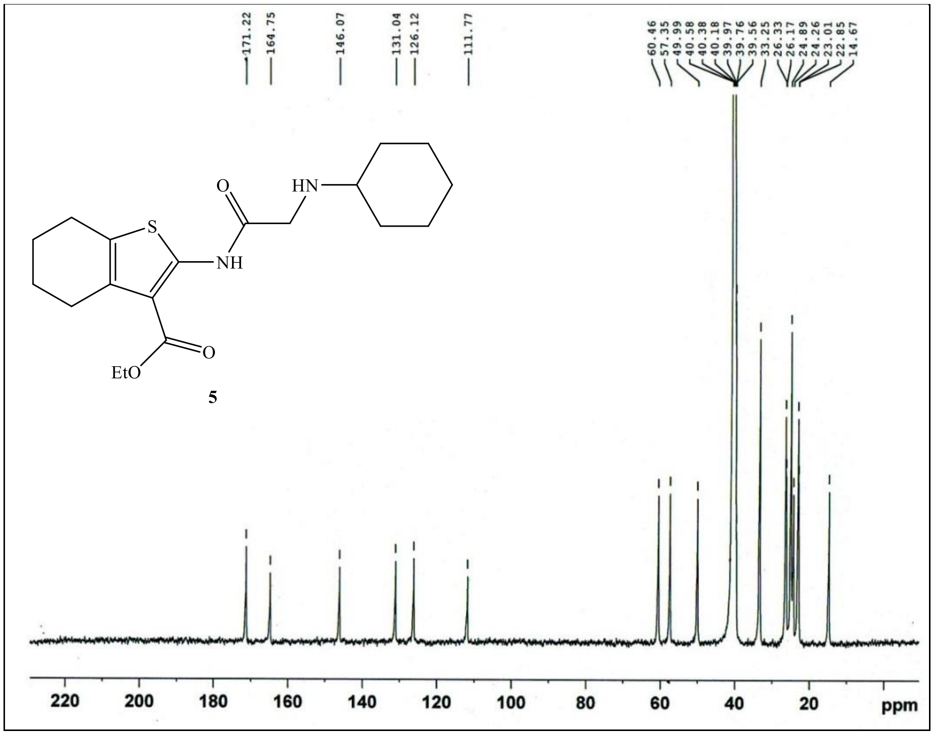

Fig. S14. ^{13}C NMR of **5** in DMSO-d_6

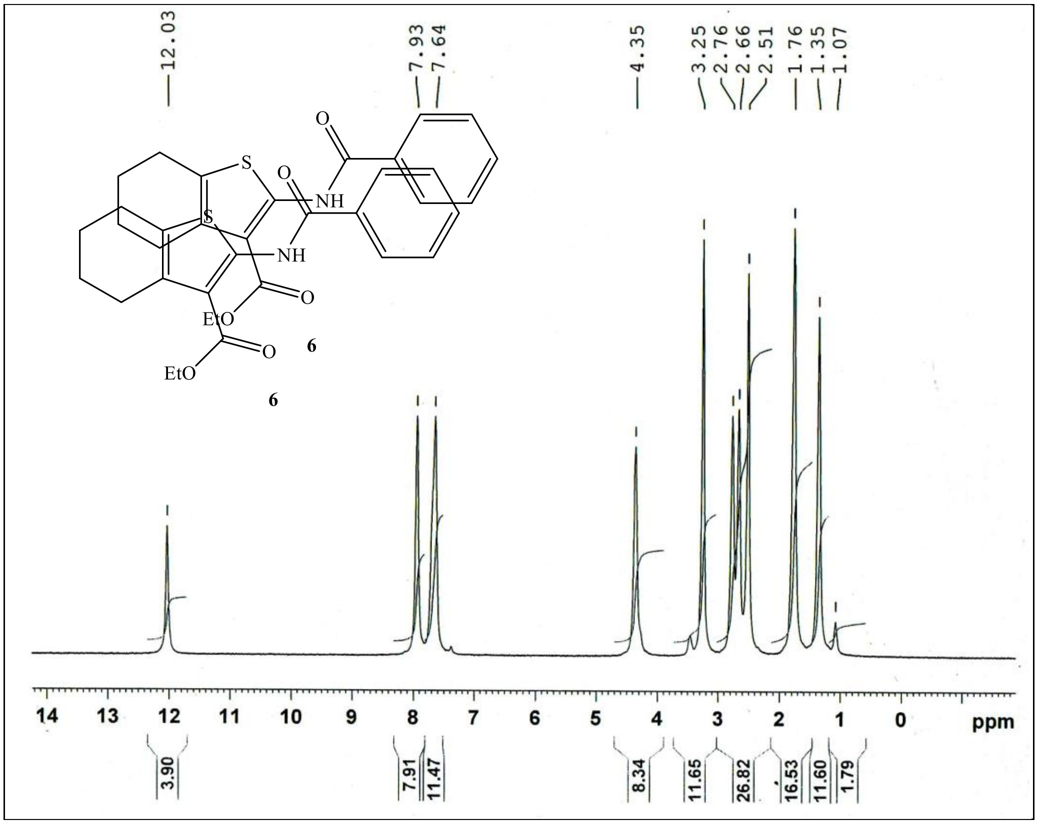

Fig. S15. ^{1}H NMR of **6** in DMSO-d_6

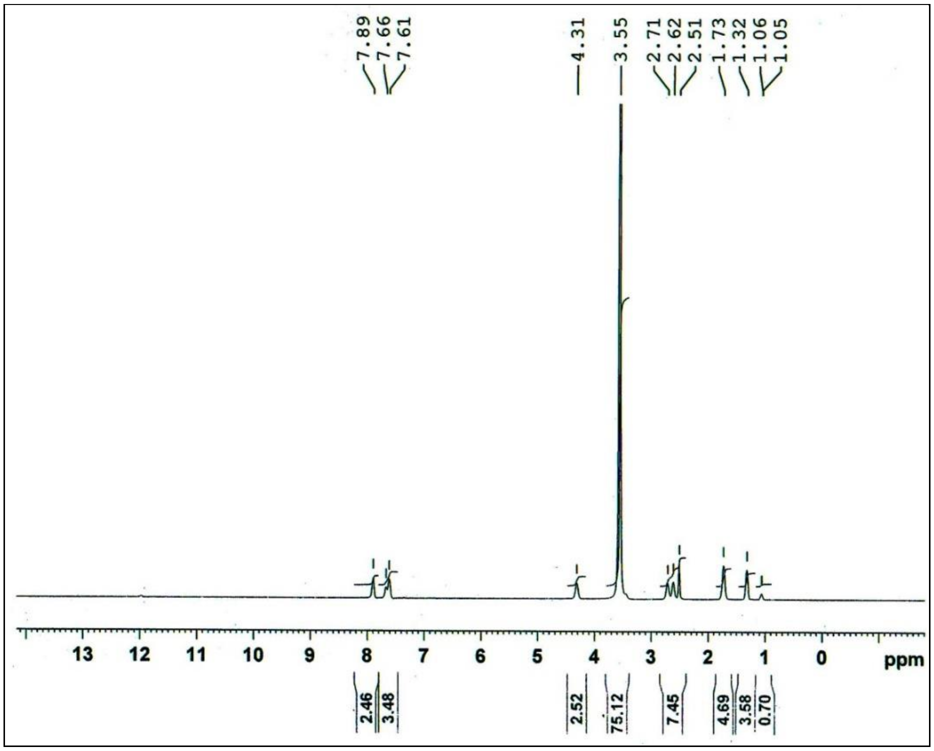

Fig. S16. ^{1}H NMR of **6** in DMSO-d_6 + D_2O

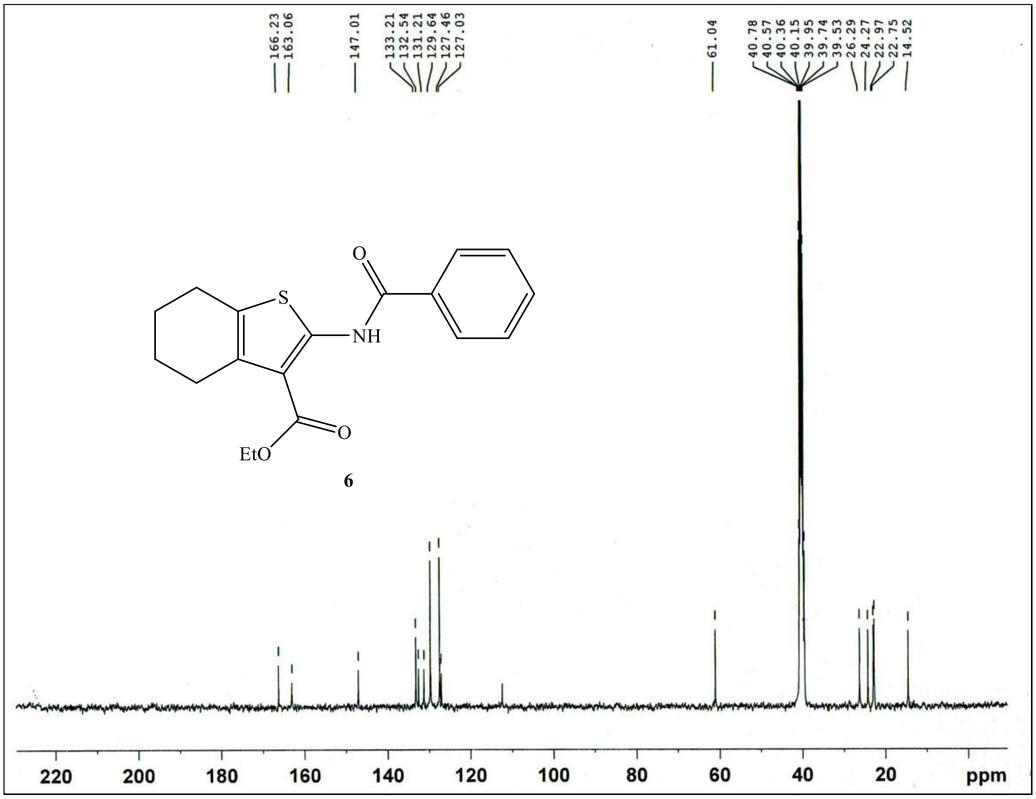

Fig. S17. ^{13}C NMR of **6** in DMSO-d_6

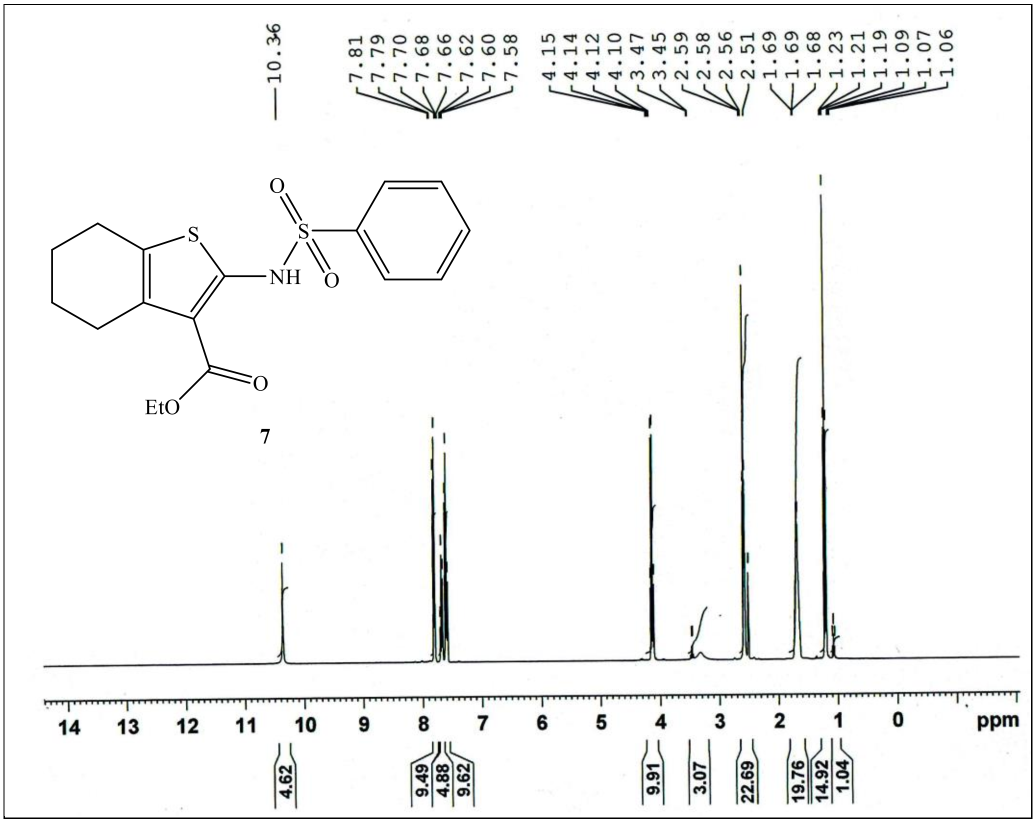

Fig. S18. ^{1}H NMR of **7** in DMSO-d_6

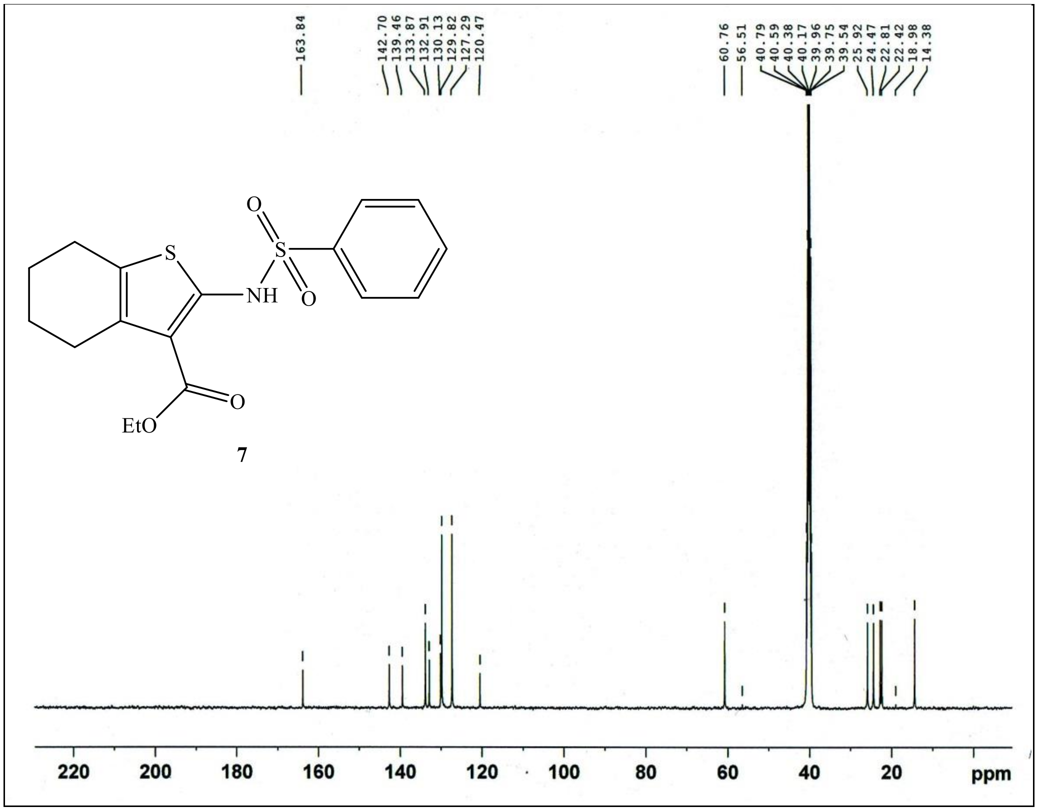

Fig. S19. ^{13}C NMR of **7** in DMSO-d_6

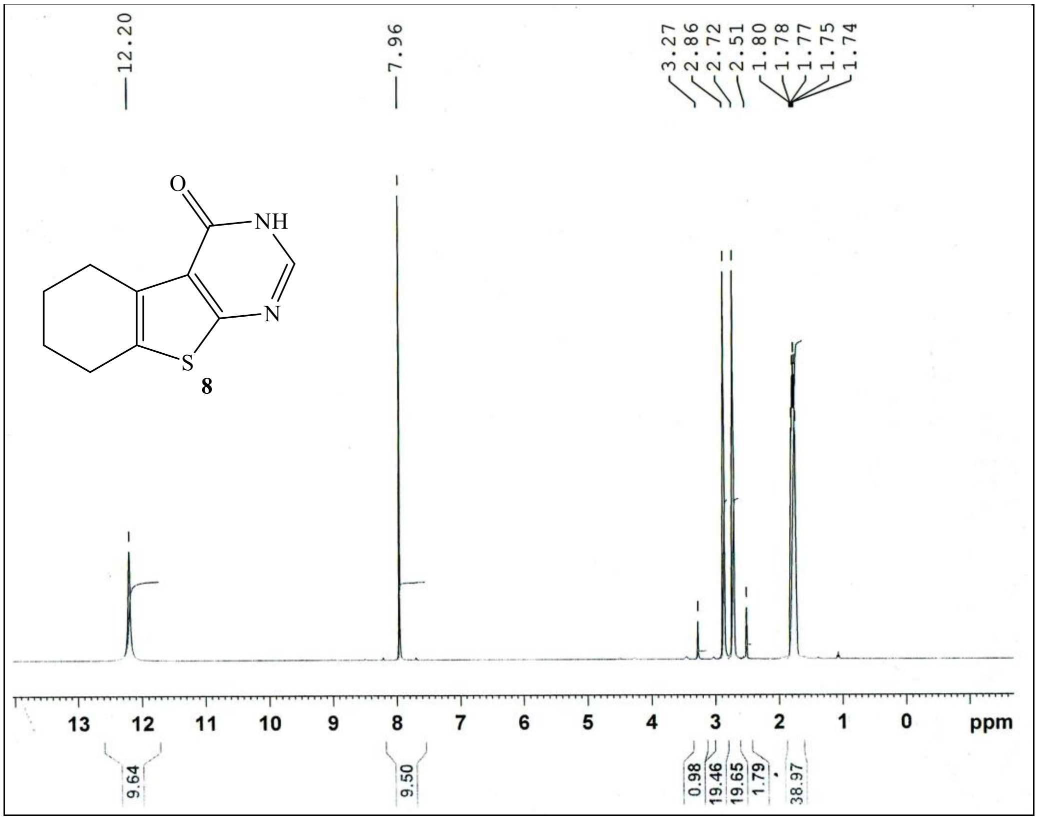

Fig. S20. ^{1}H NMR of **8** in DMSO-d_6

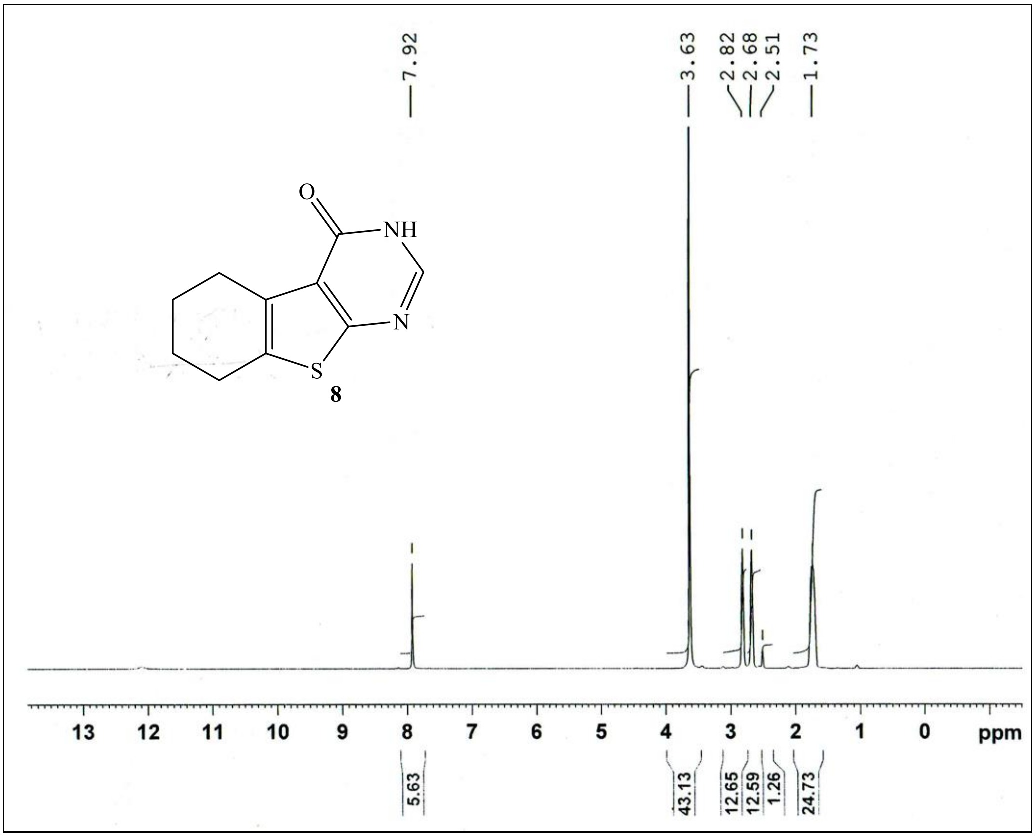

Fig. S21. ^{1}H NMR of **8** in DMSO-d_6 + D_2O

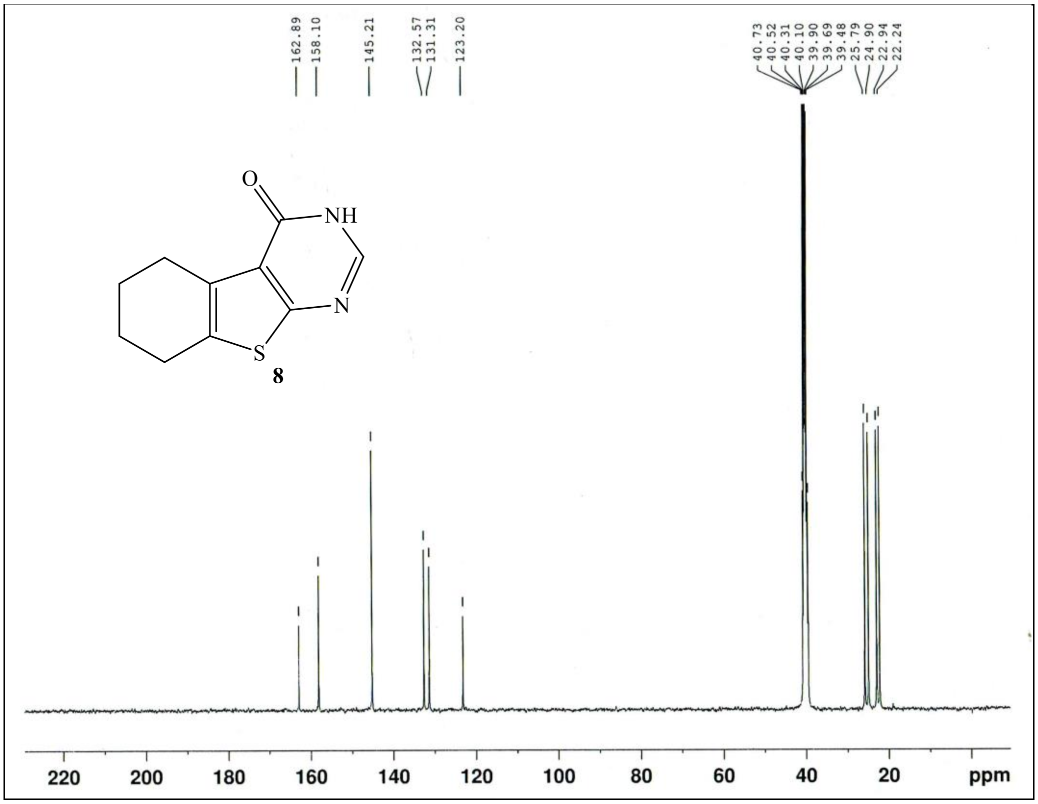

Fig. S22. ^{13}C NMR of **8** in DMSO-d_6

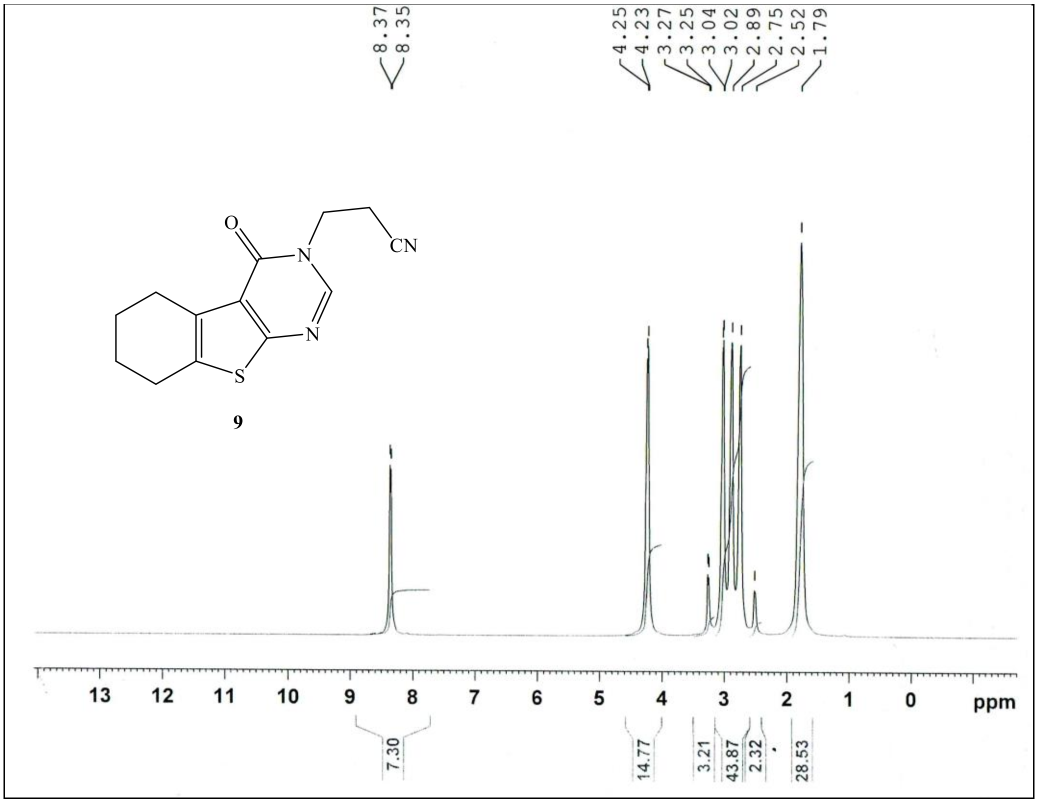

Fig. S23. ^{1}H NMR of **9** in DMSO-d_6

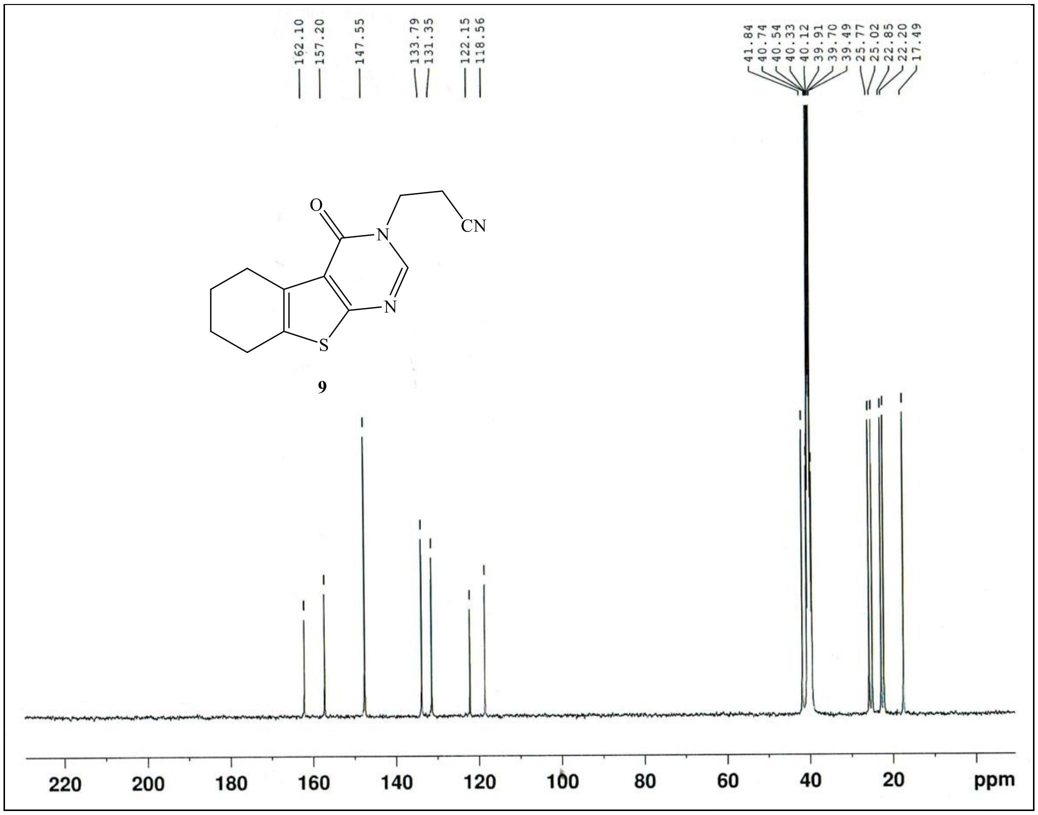

Fig. S24. ^{13}C NMR of **9** in DMSO-d_6

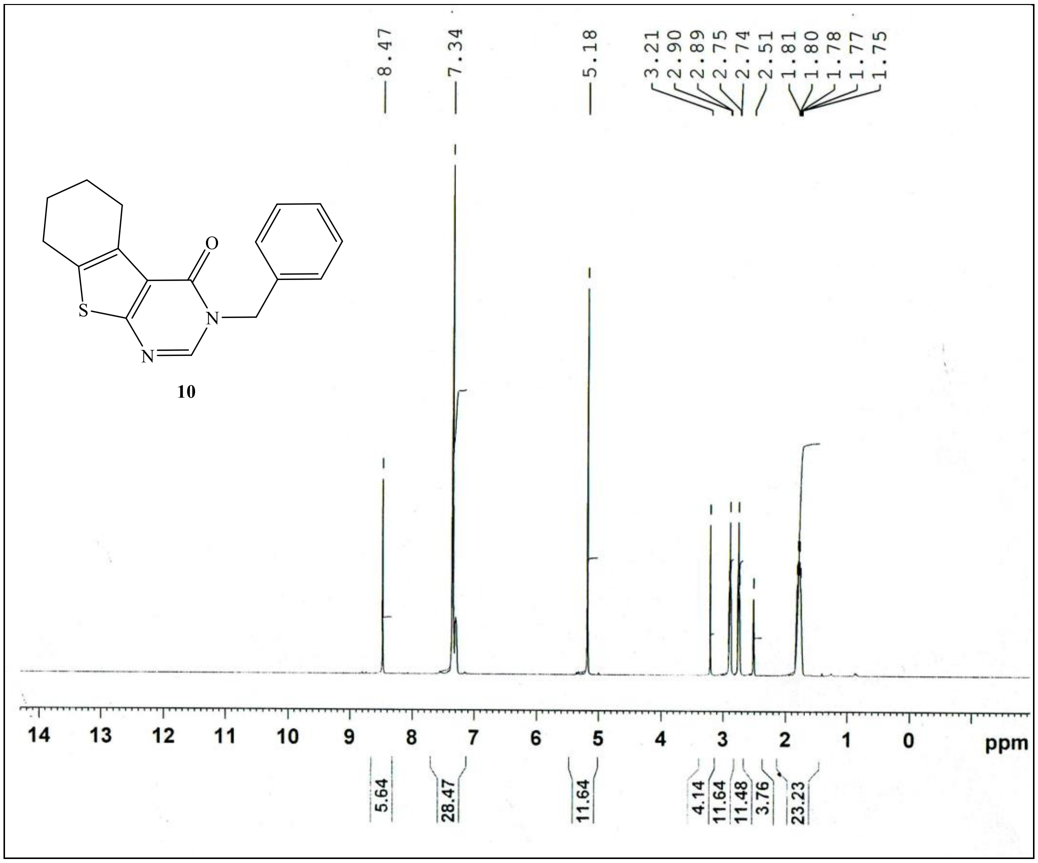

Fig. S25. ^{1}H NMR of **10** in DMSO-d_6

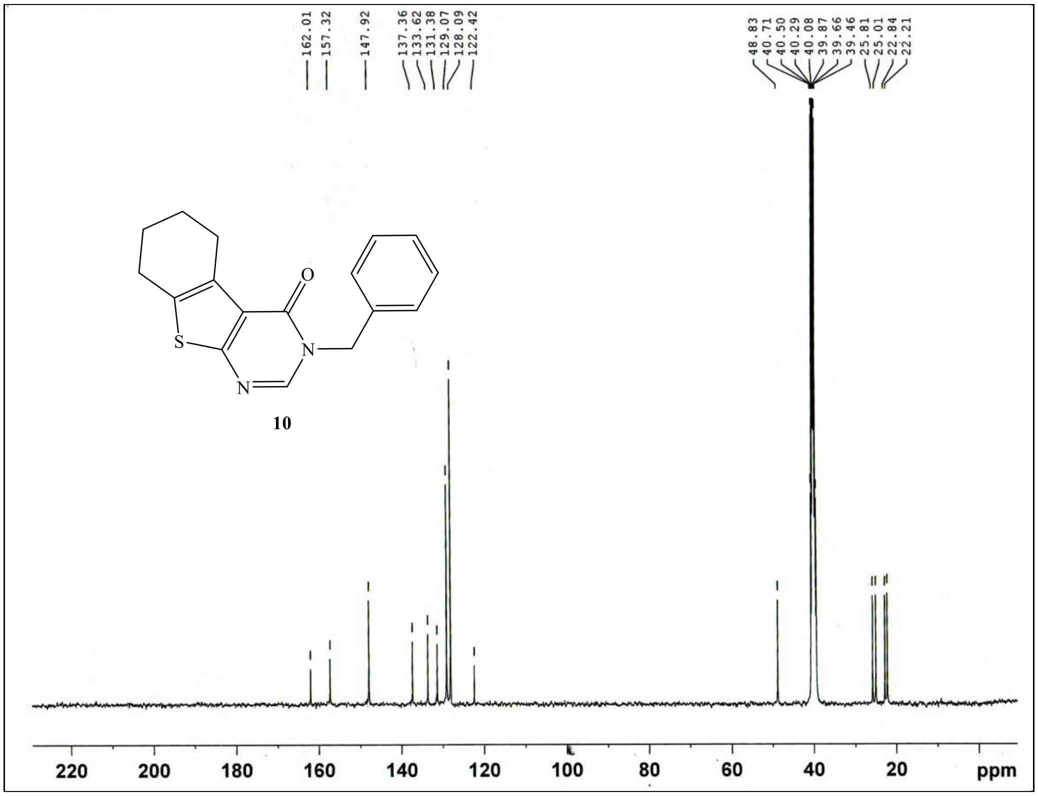

Fig. 2S26. ^{13}C NMR of **10** in DMSO-d_6

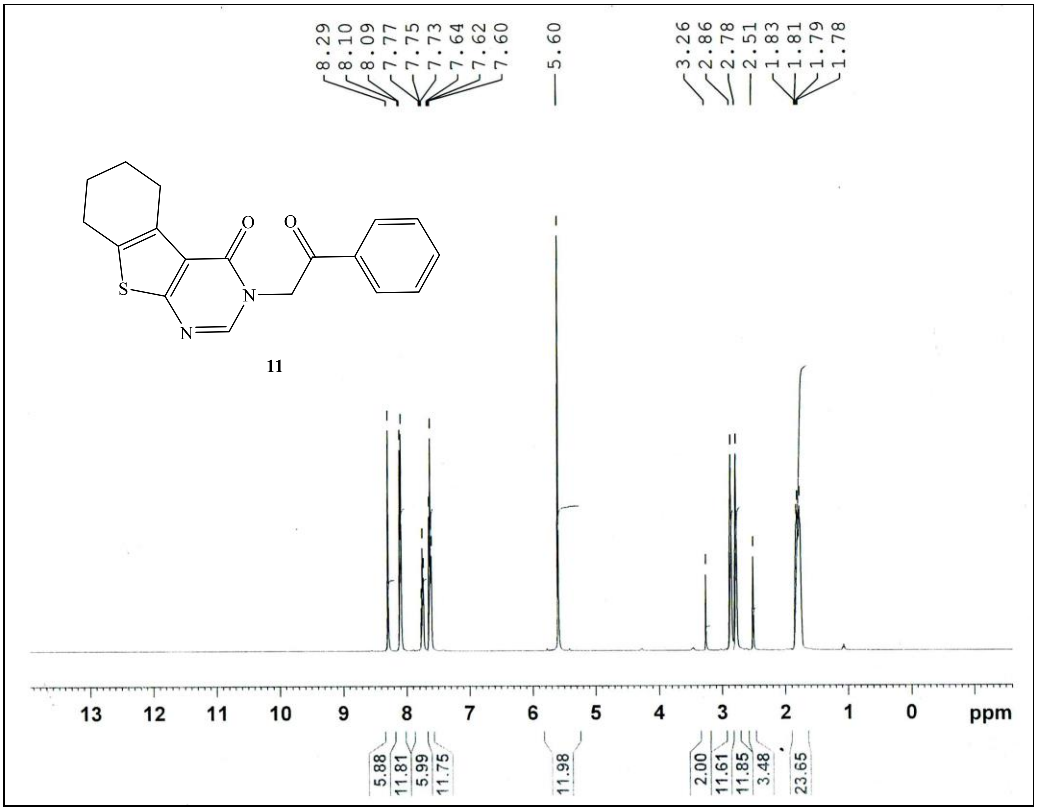

Fig. S27. ^{1}H NMR of **11** in DMSO-d_6

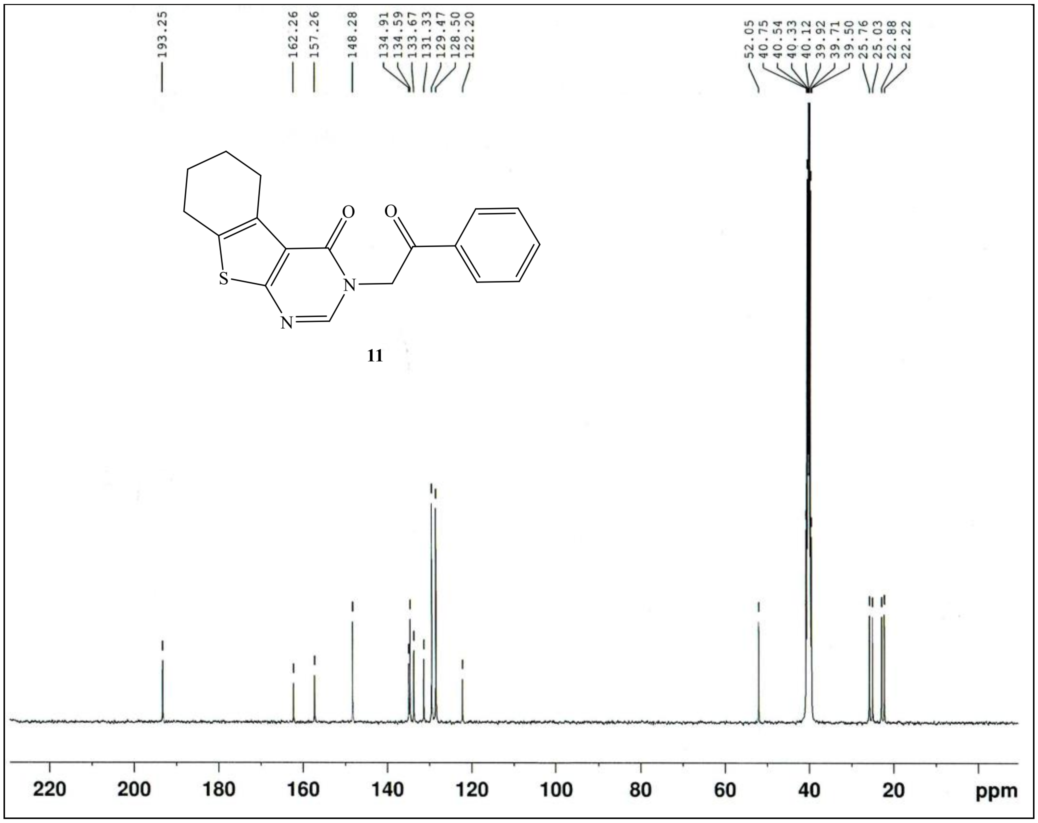

Fig. S28. ^{13}C NMR of **11** in DMSO-d_6

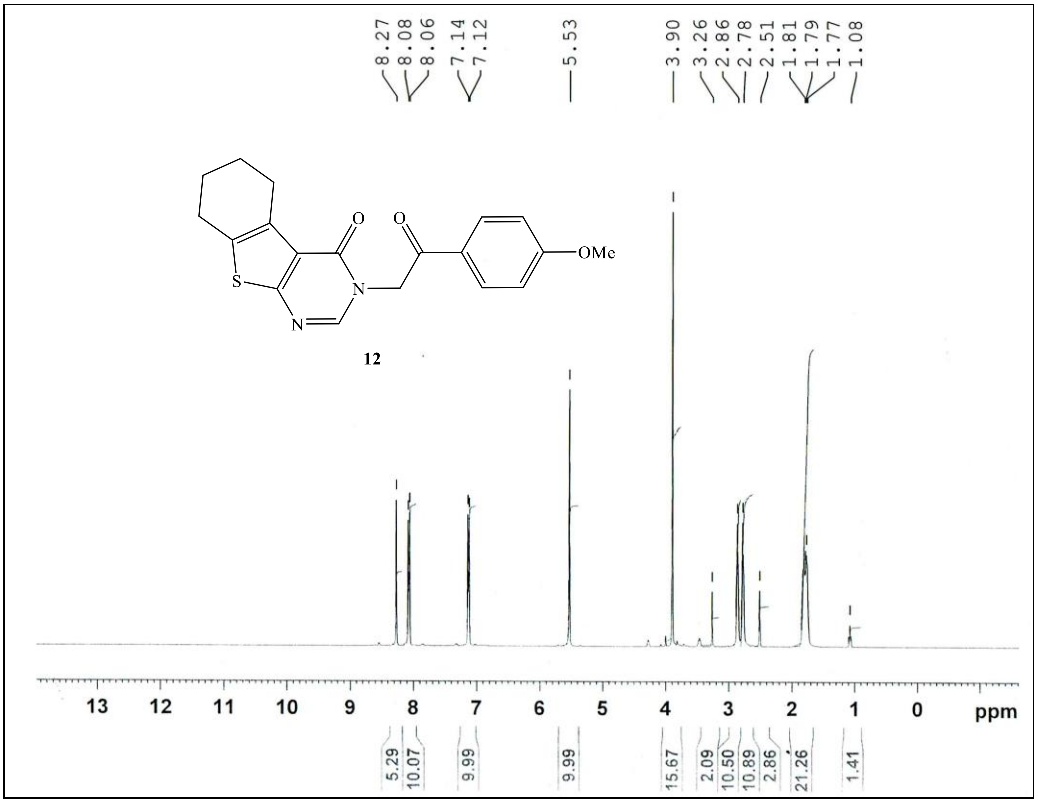

Fig. S29. ^{1}H NMR of **12** in DMSO-d_6

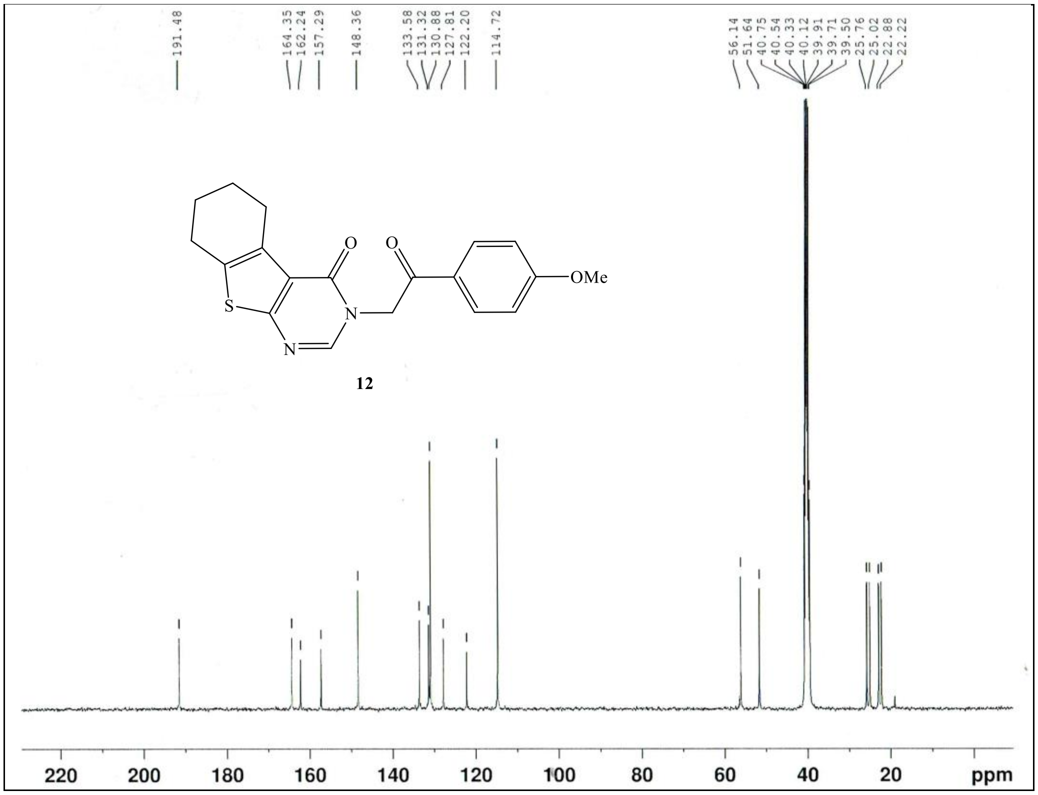

Fig. S30. ^{13}C NMR of **12** in DMSO-d_6

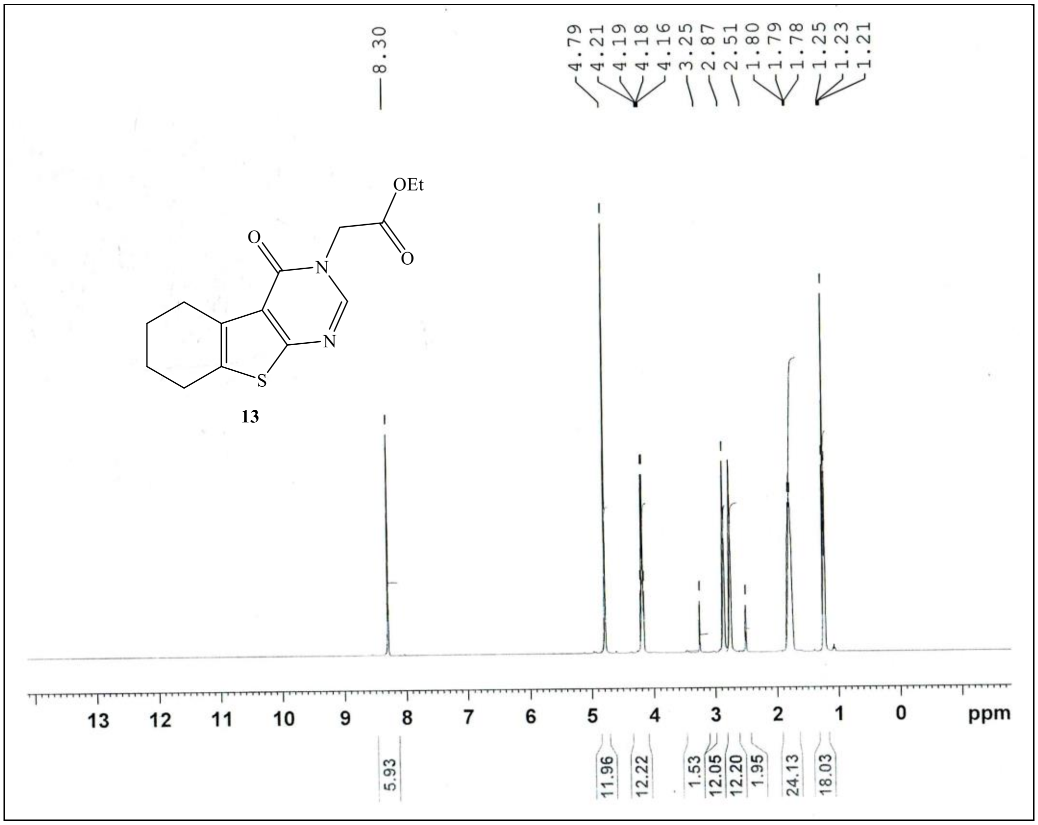

Fig. S31. ^{1}H NMR of **13** in DMSO-d_6

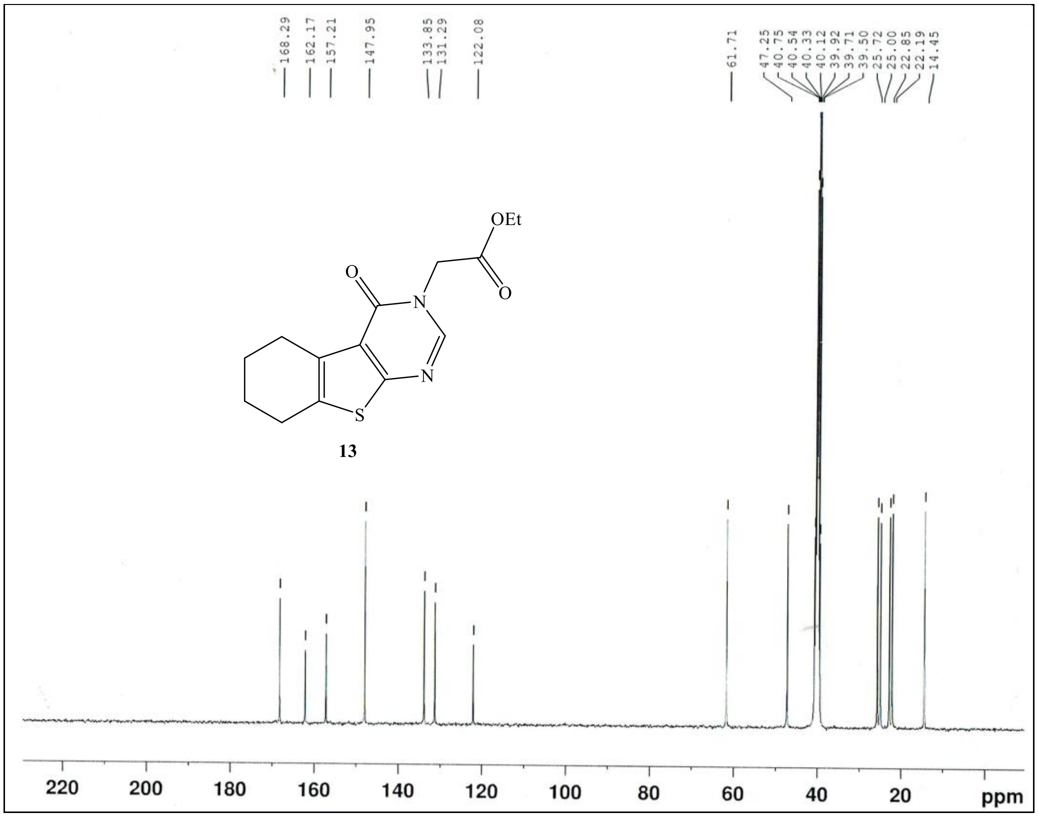

Fig. S32. ^{13}C NMR of **13** in DMSO-d_6

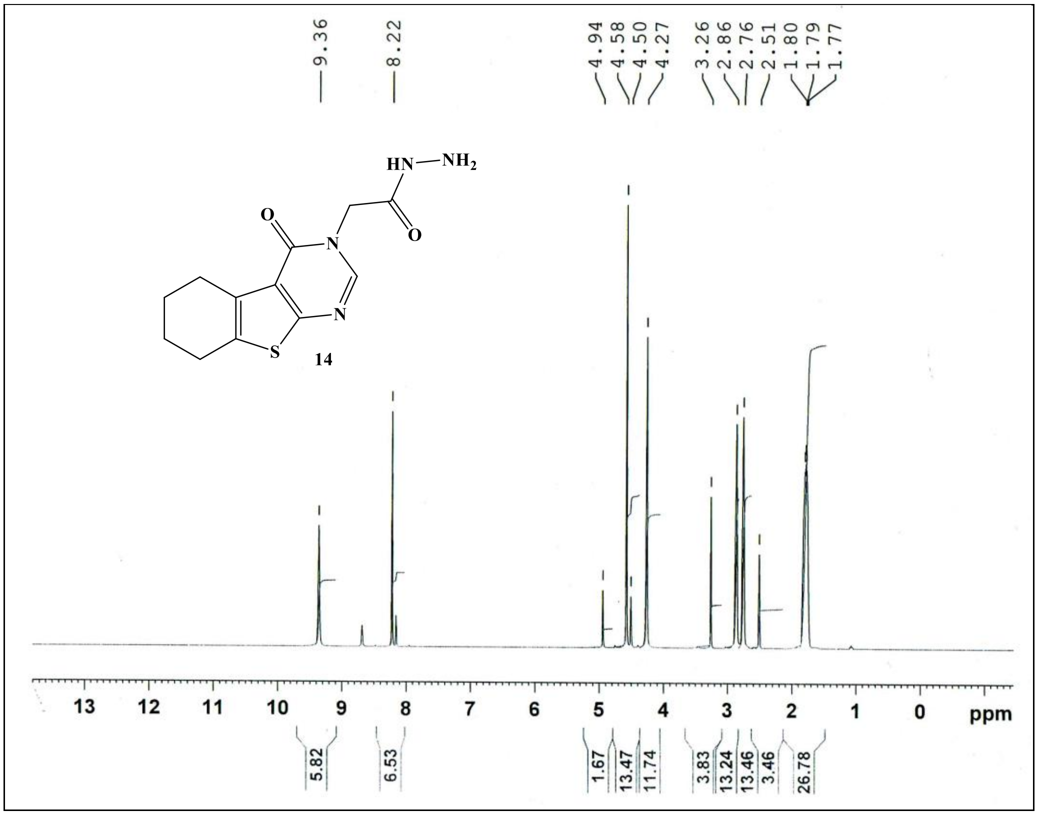

Fig. S33. ¹H NMR of **14** in DMSO-d_6

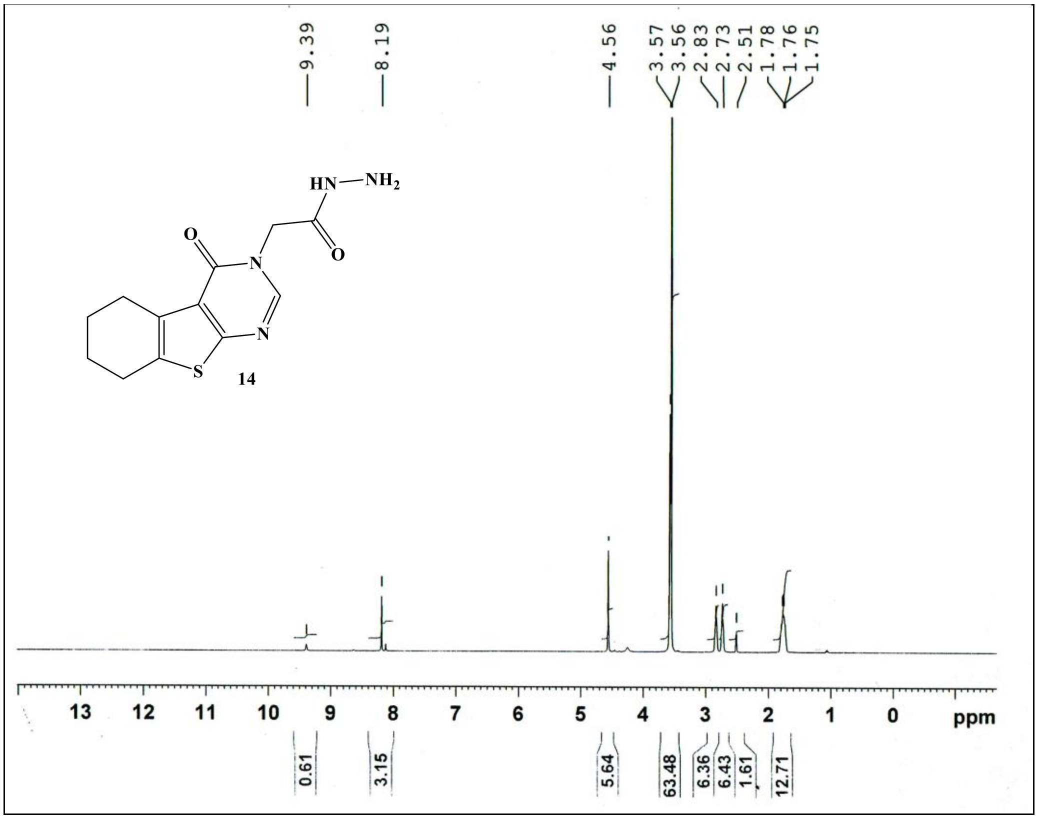

Fig. S34. ^{1}H NMR of **14** in DMSO-d_6 + D_2O

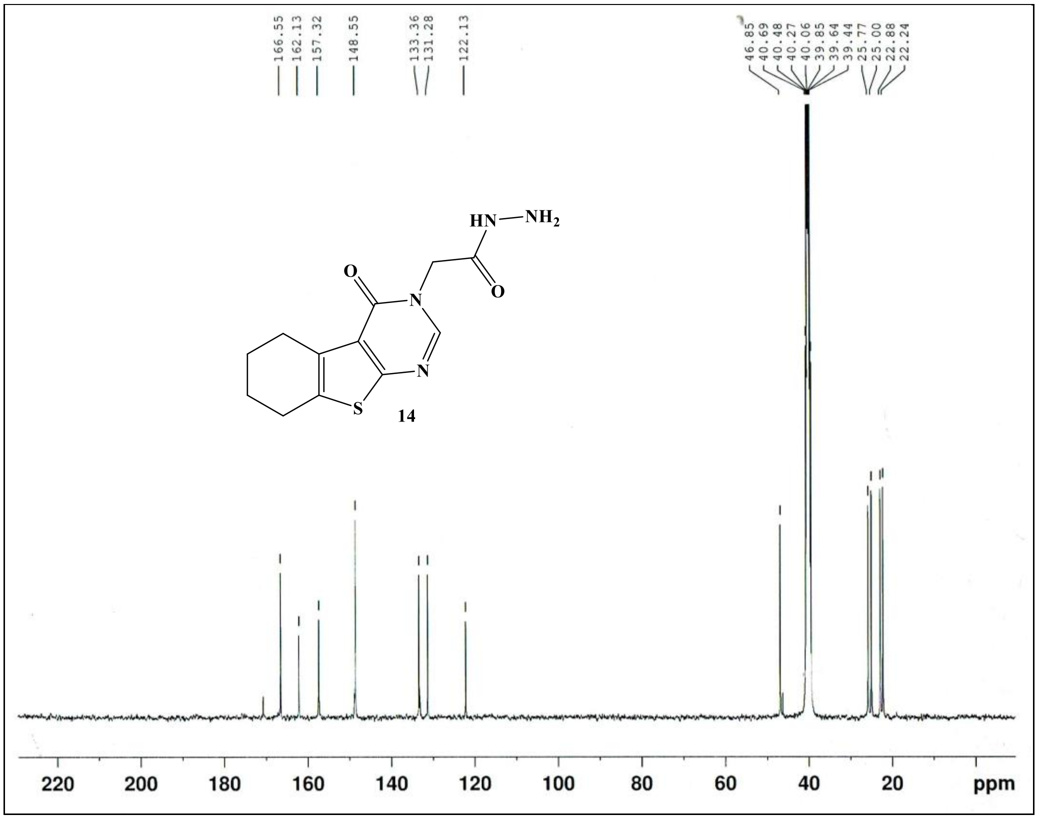

Fig. S35. ^{13}C NMR of **14** in DMSO-d_6

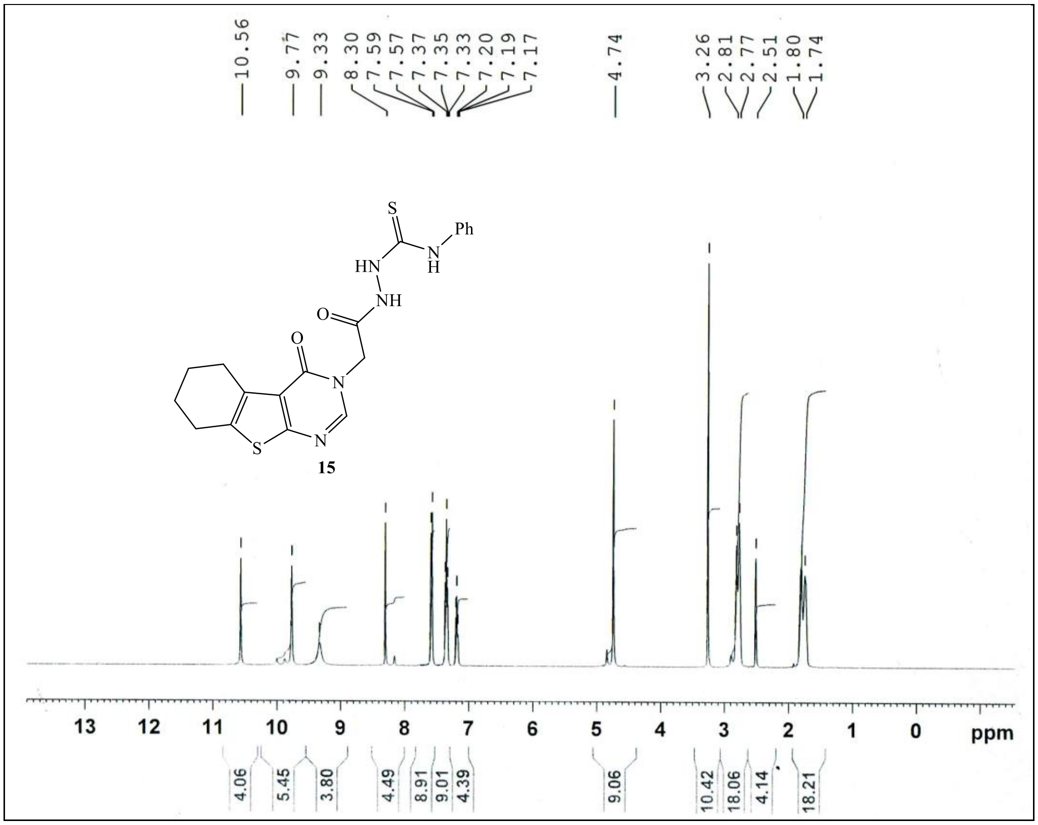

Fig. S36. ^{1}H NMR of **15** in DMSO-d_6

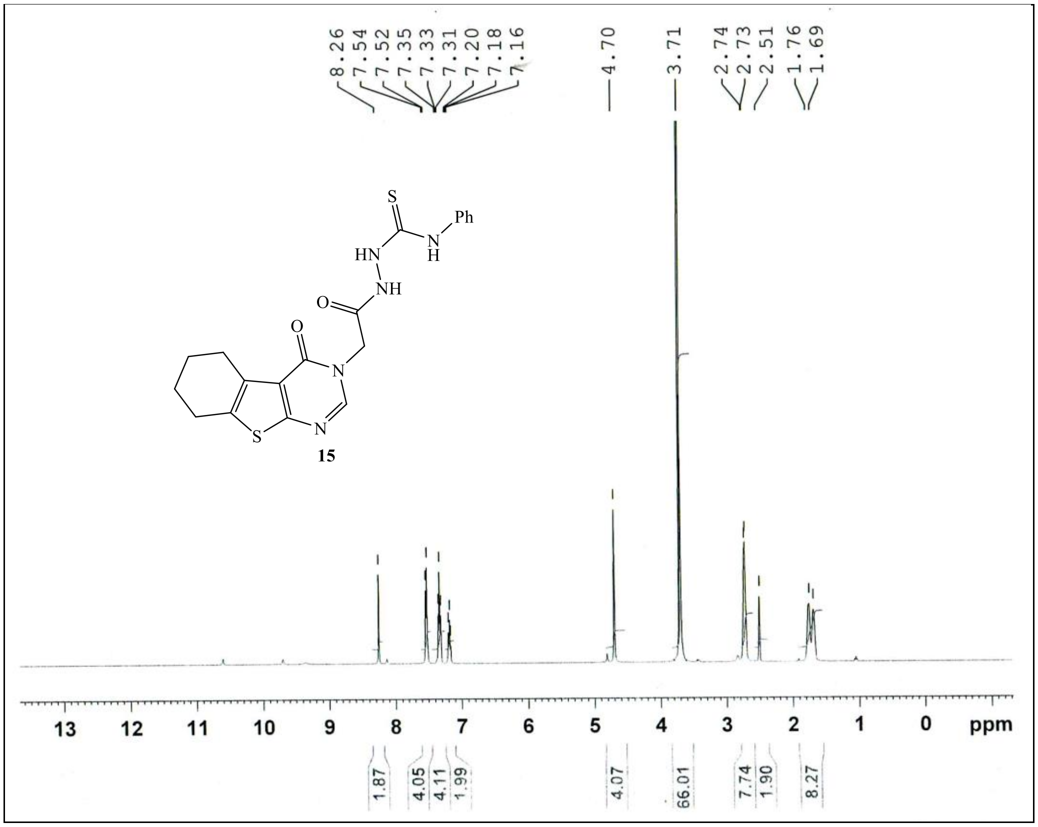

Fig. S37. ^{1}H NMR of **15** in DMSO-d_6 + D_2O

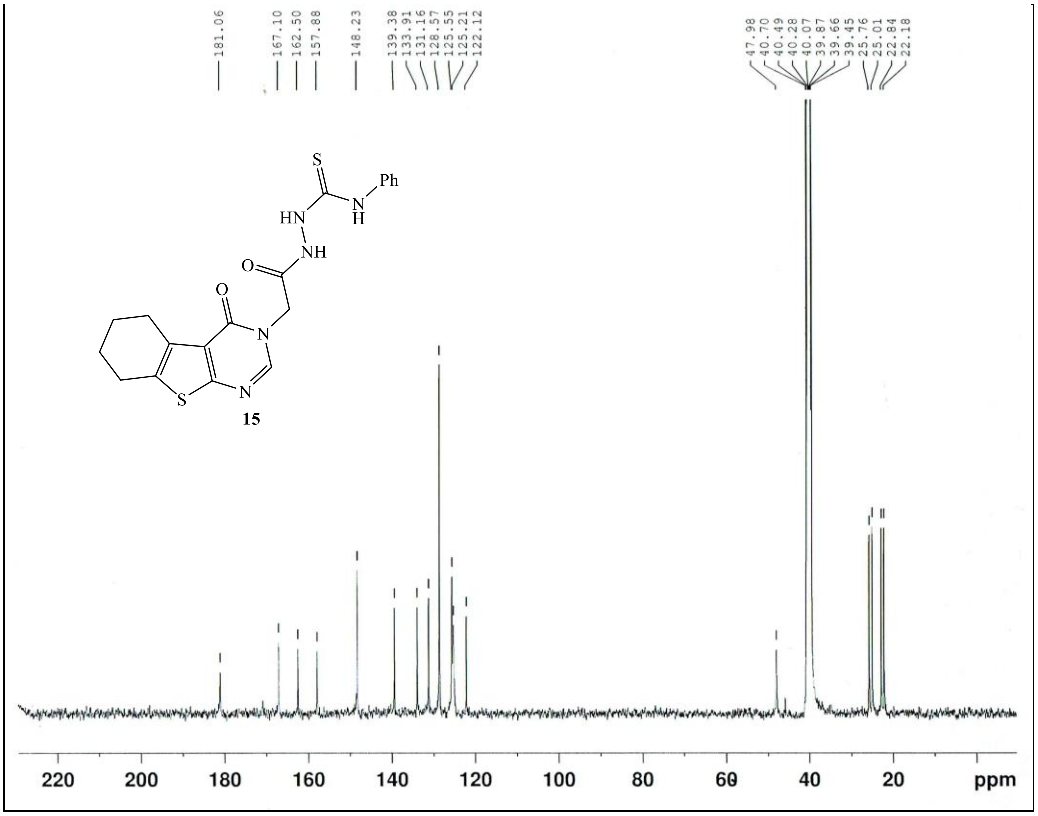

Fig. S38. ^{13}C NMR of **15** in DMSO-d_6

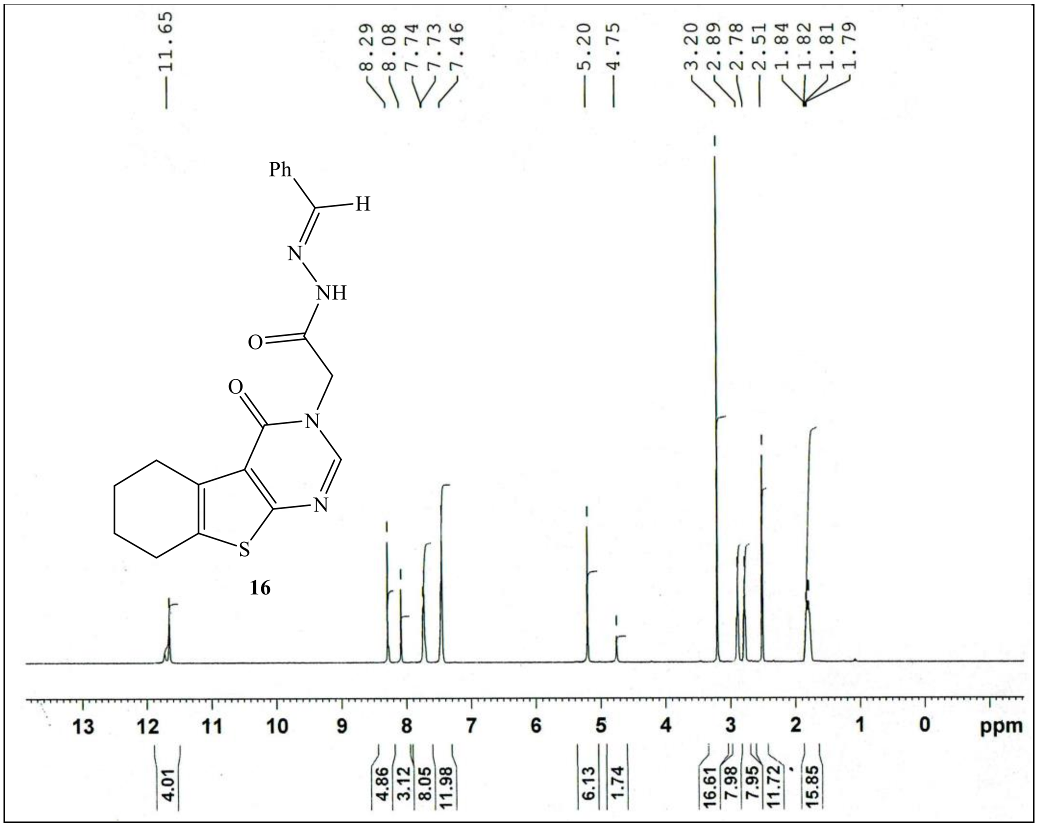

Fig. S39. ^{1}H NMR of **16** in DMSO-d_6

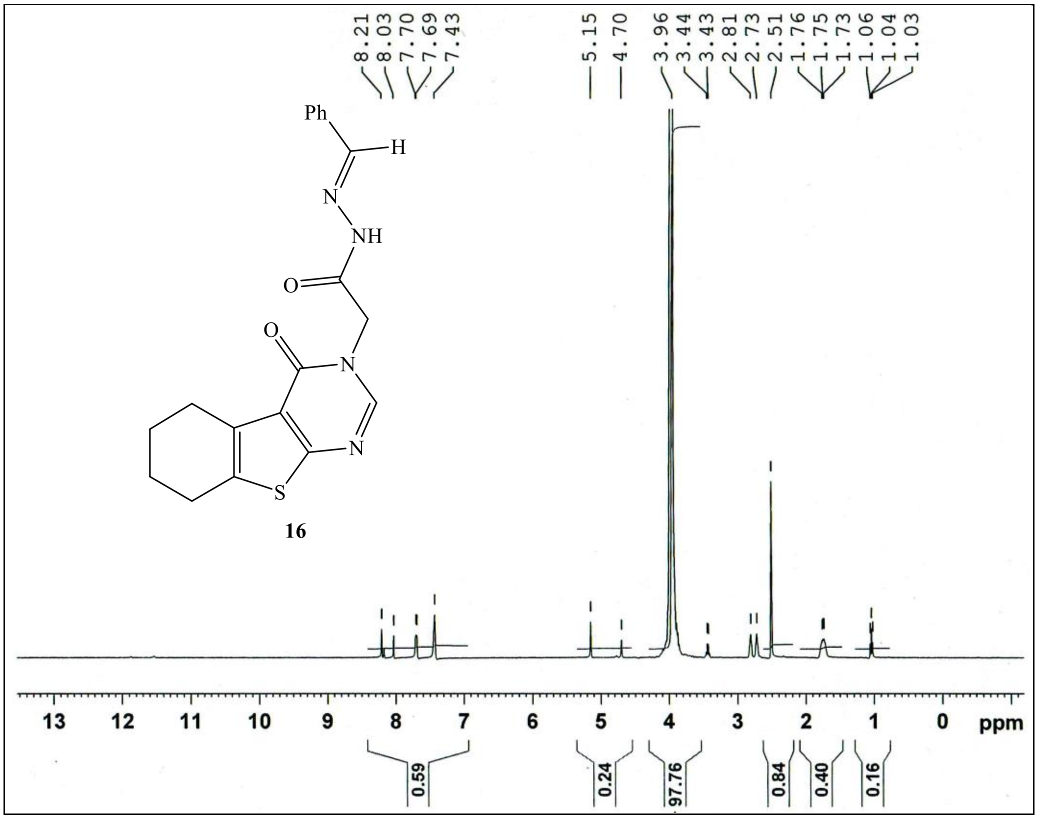

Fig. S40. ^{1}H NMR of **16** in DMSO-d_6 + D_2O

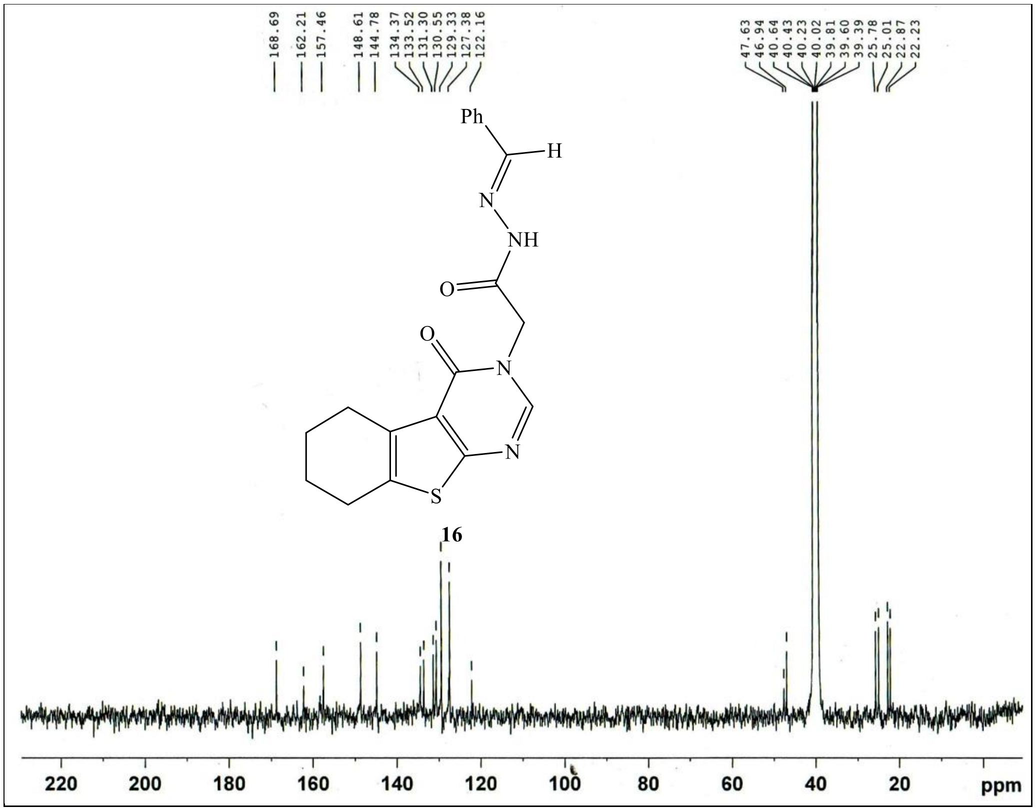

Fig. S41. ^{13}C NMR of **16** in DMSO-d_6

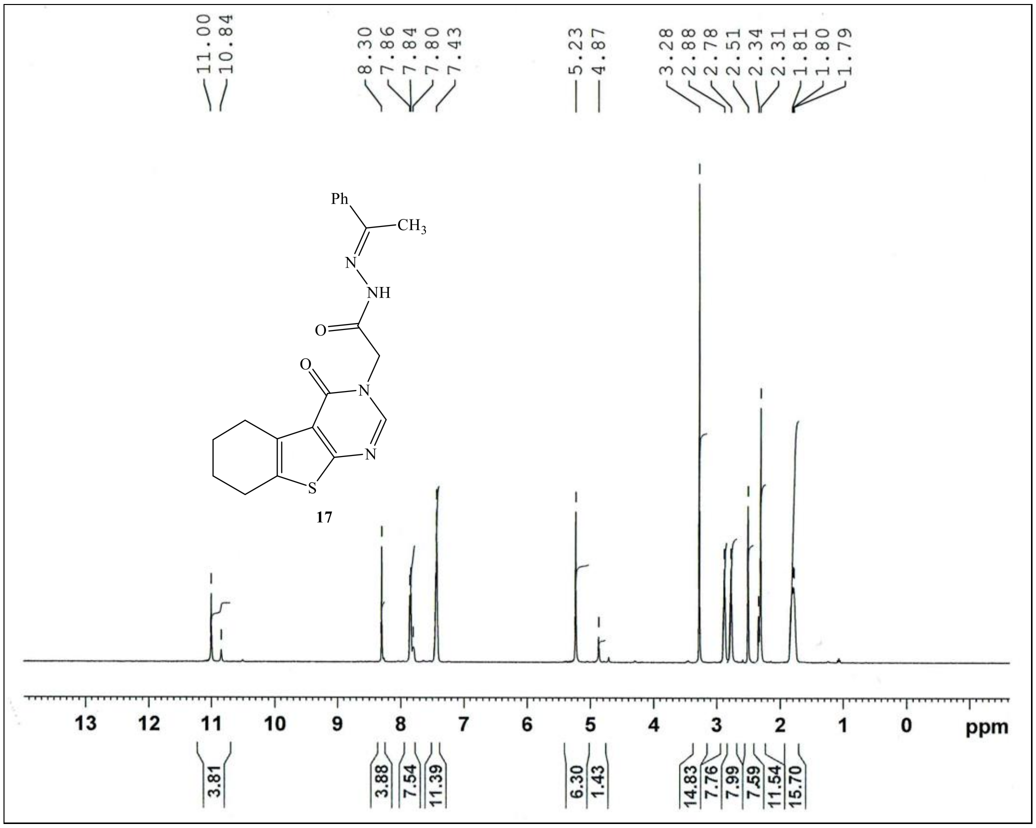

Fig. S42. ^{1}H NMR of **17** in DMSO-d_6

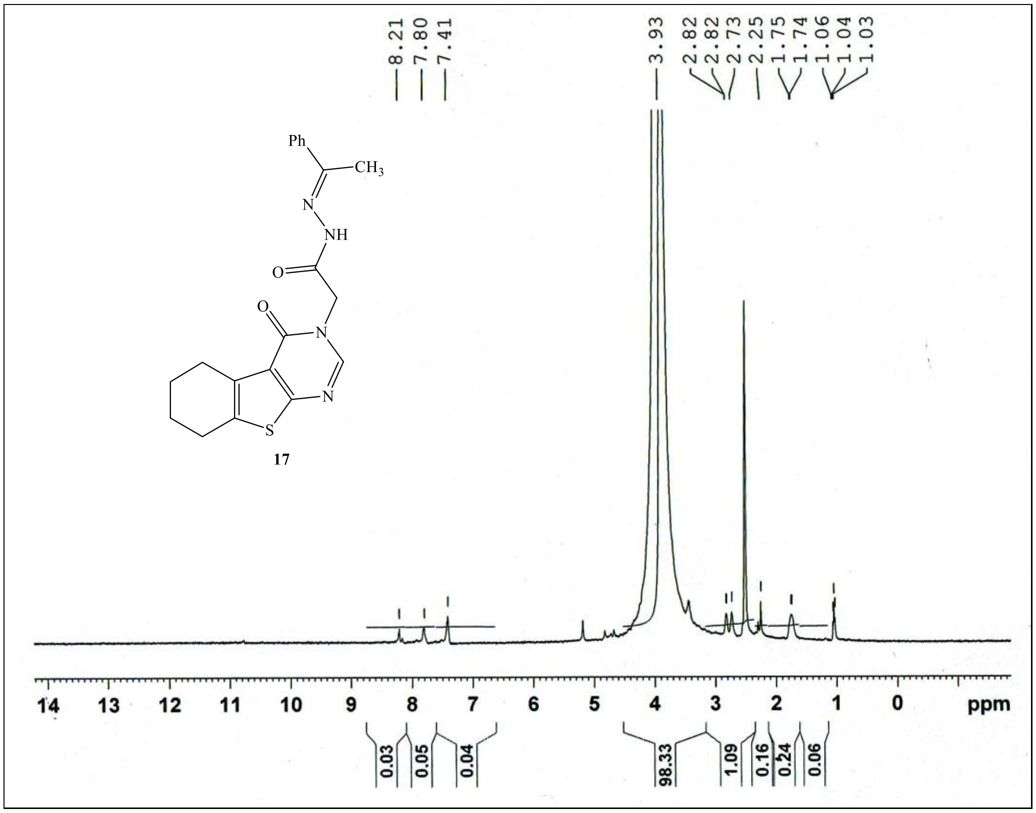

Fig. S43. ^{1}H NMR of **17** in DMSO-d_6 + D_2O

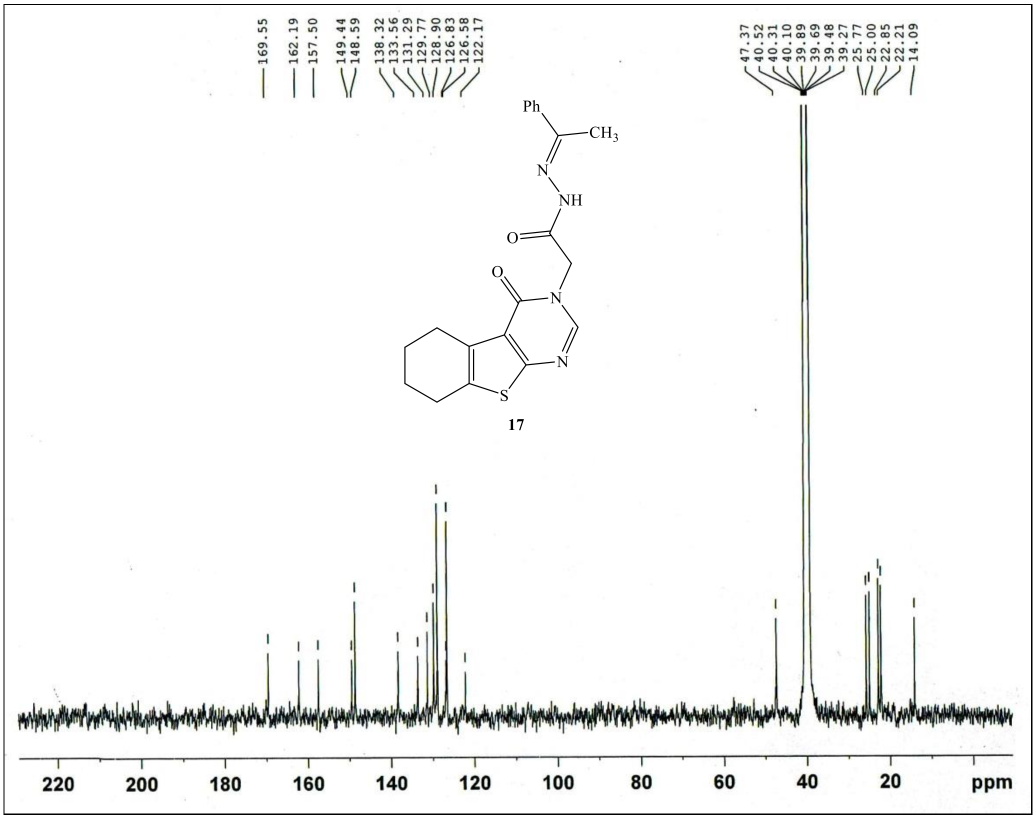

Fig. S44. ^{13}C NMR of **17** in DMSO-d_6

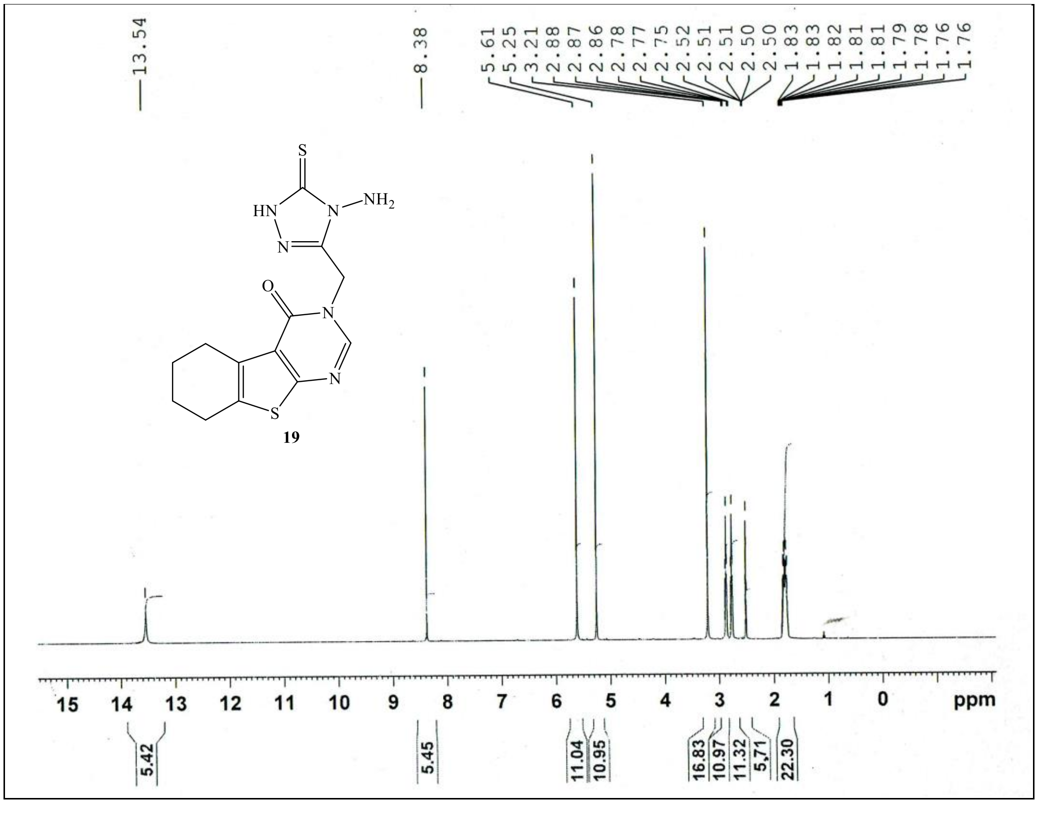

Fig. S45. ^{1}H NMR of **19** in DMSO-d_6

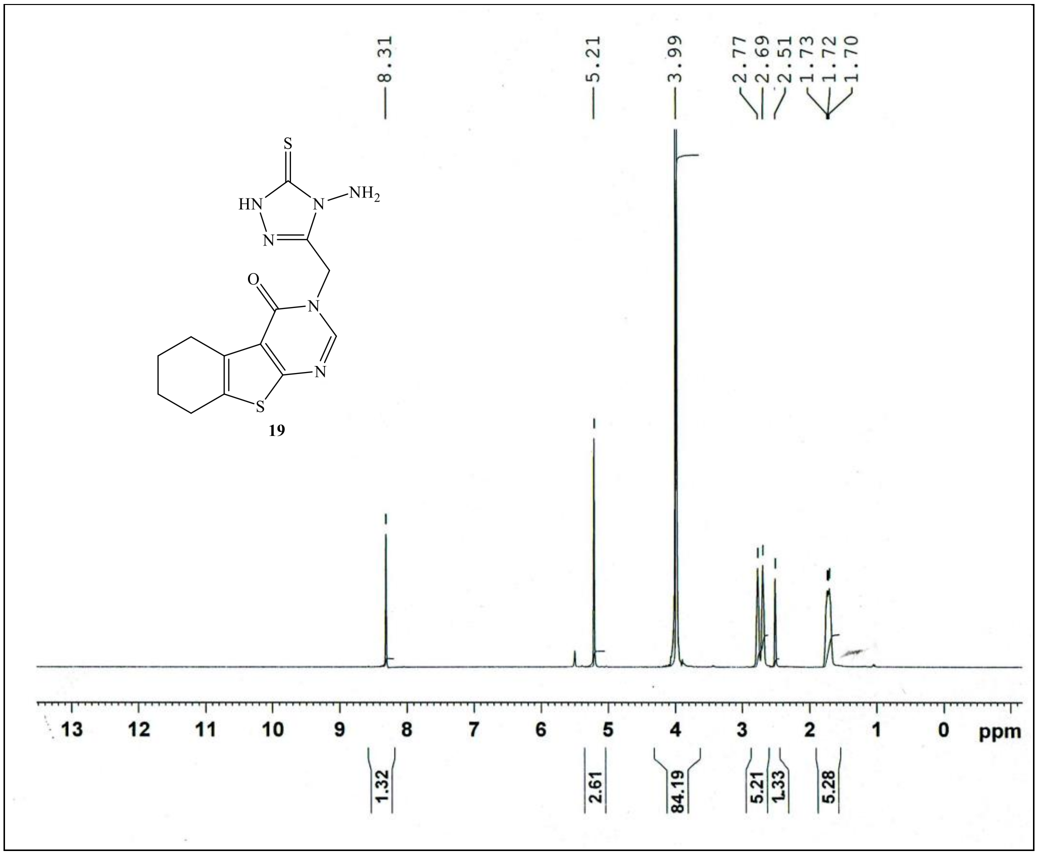

Fig. S46. ^{1}H NMR of **19** in DMSO-d_6 + D_2O

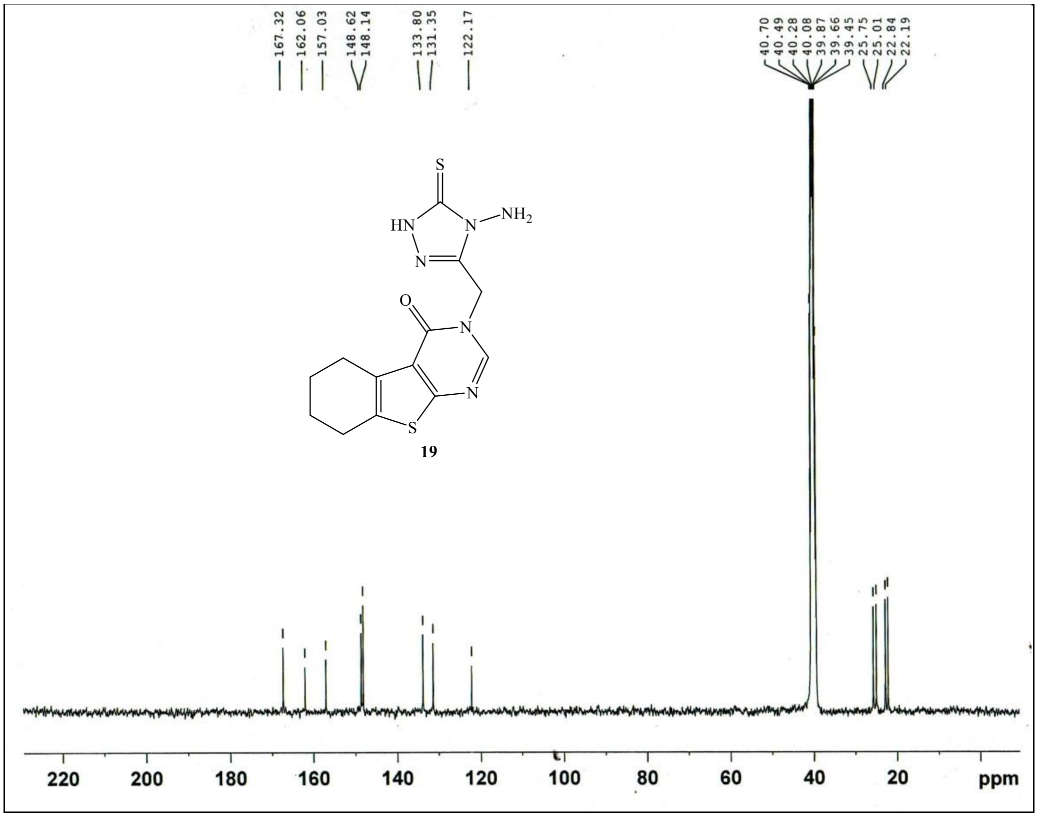

Fig. S47. ^{13}C NMR of **19** in DMSO-d_6

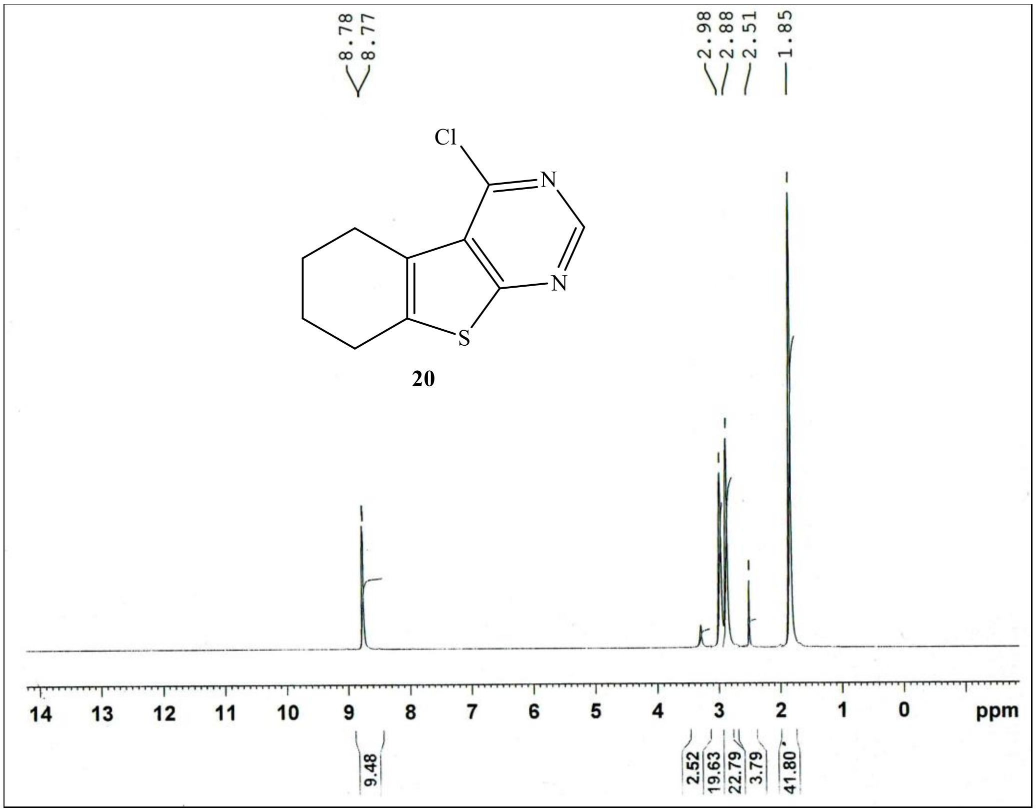

Fig. S48. ^{1}H NMR of **20** in DMSO-d_6

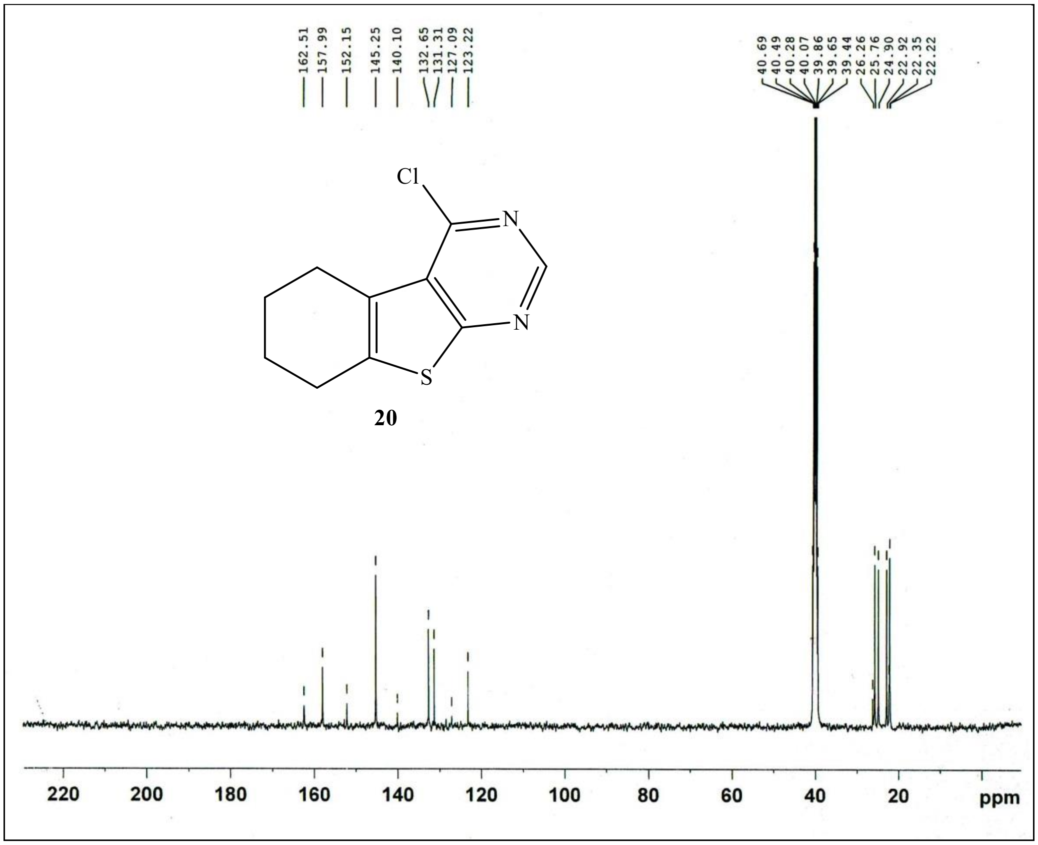

Fig. S49. ^{13}C NMR of **20** in DMSO-d_6

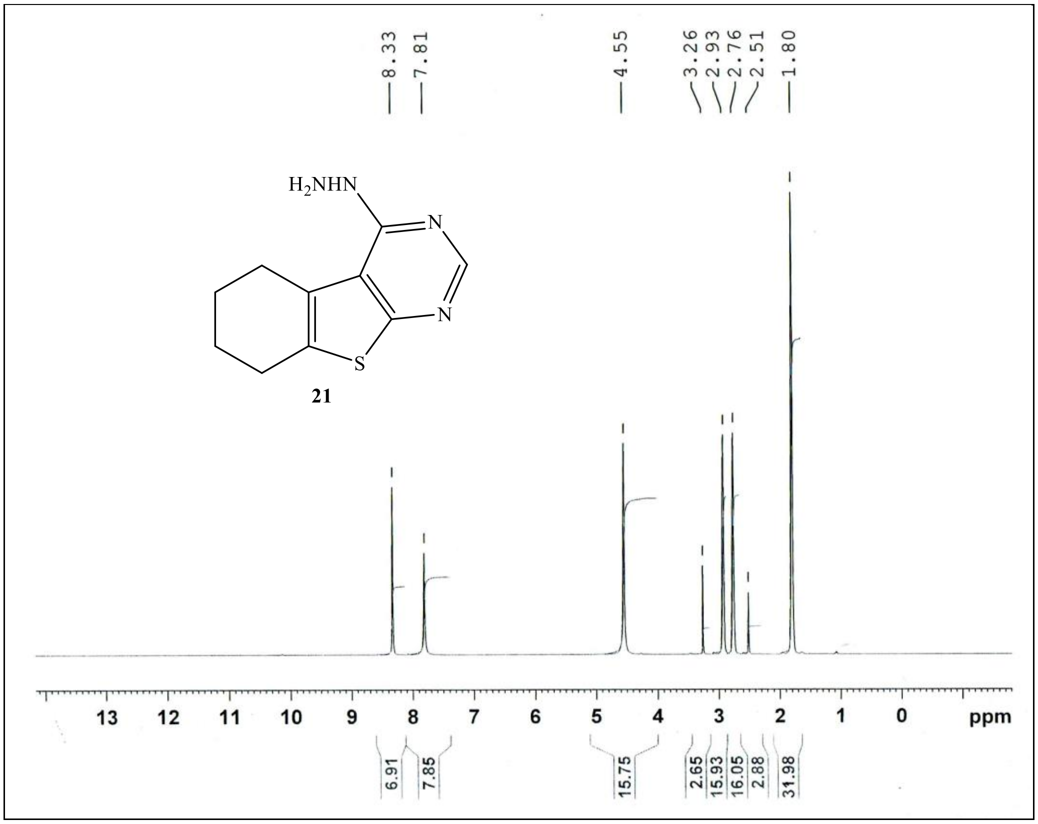

Fig. S50. ^{1}H NMR of **21** in DMSO-d_6

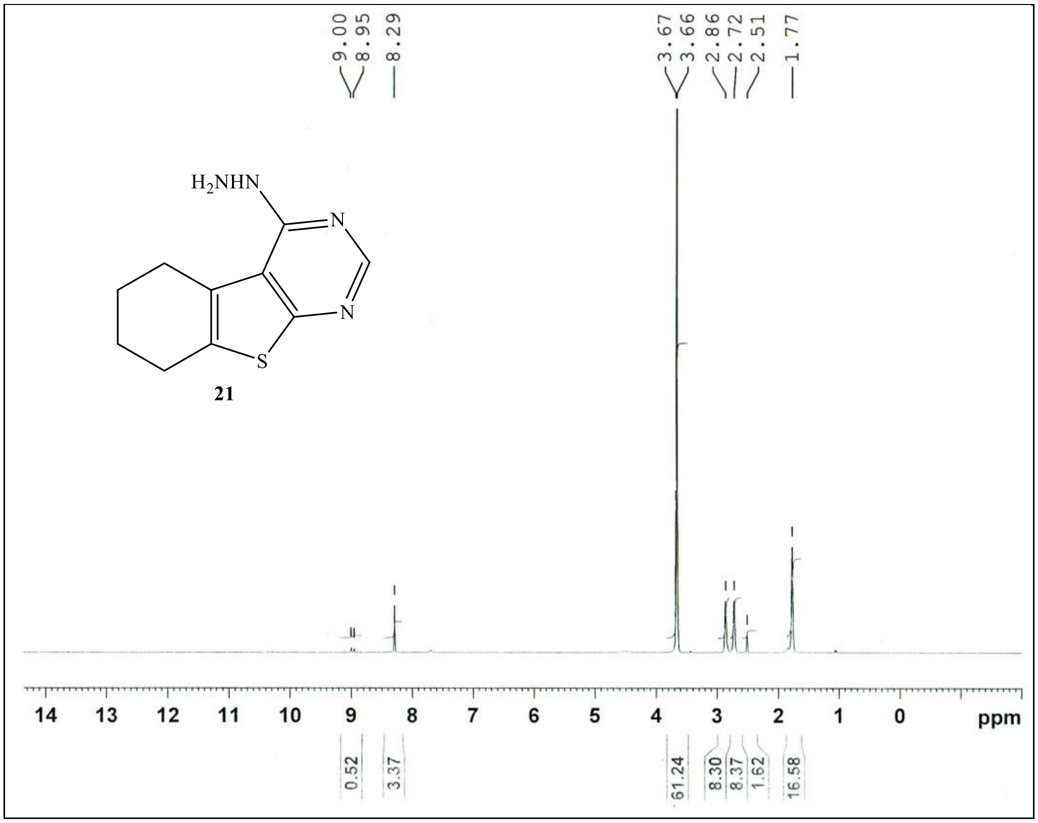

Fig. S51. ^{1}H NMR of **21** in DMSO-d_6 + D_2O

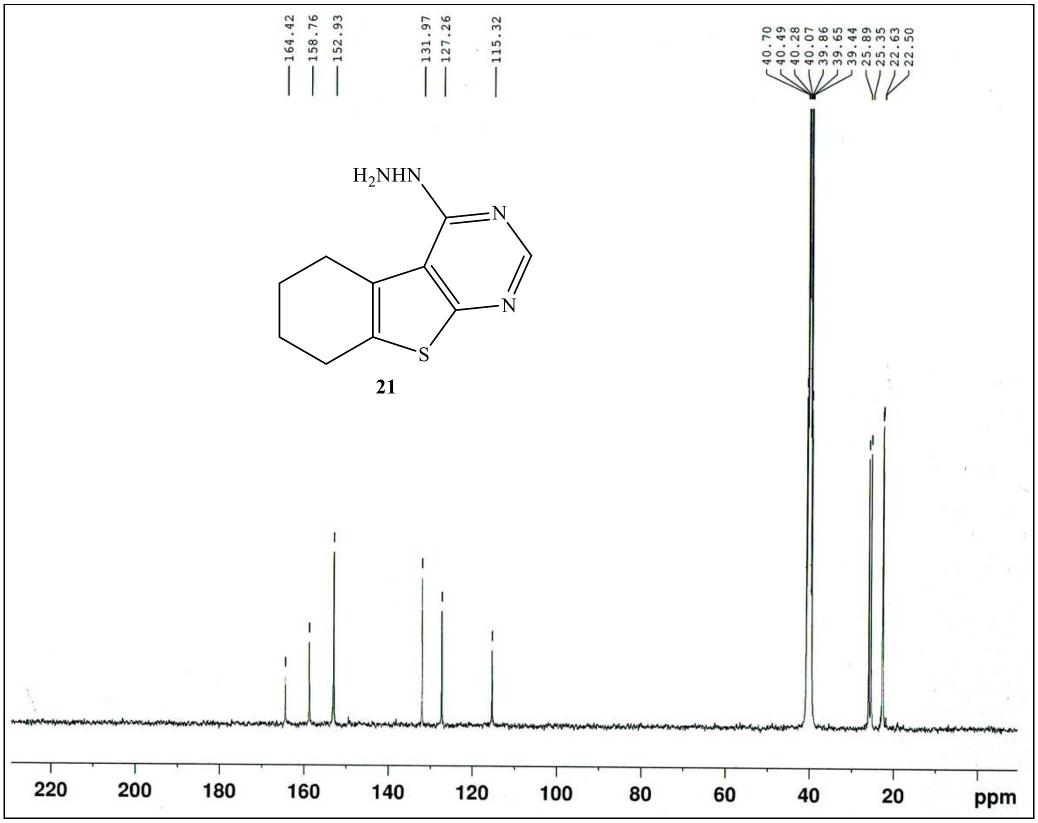

Fig. S52. ^{13}C NMR of **21** in DMSO-d_6

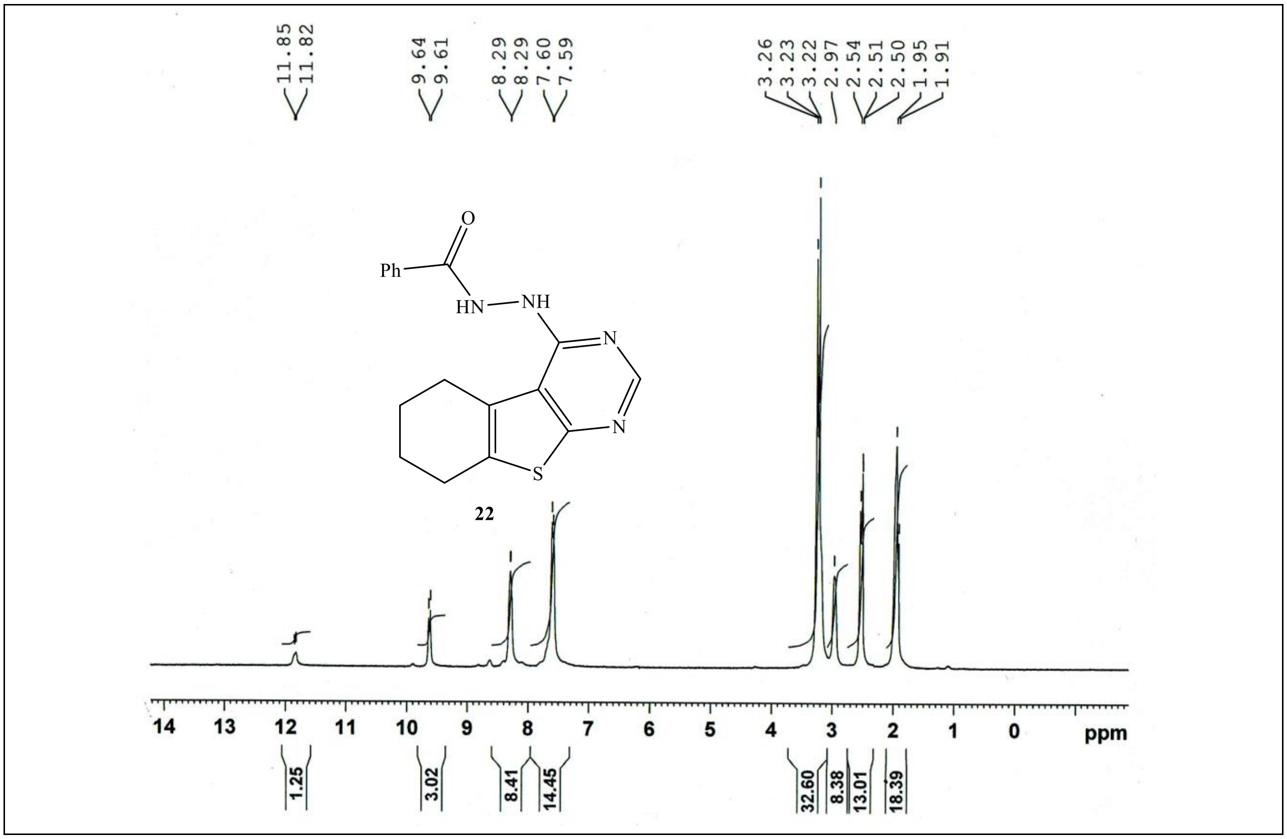

Fig. S53. ^{1}H NMR of **22** in DMSO-d_6 + D_2O

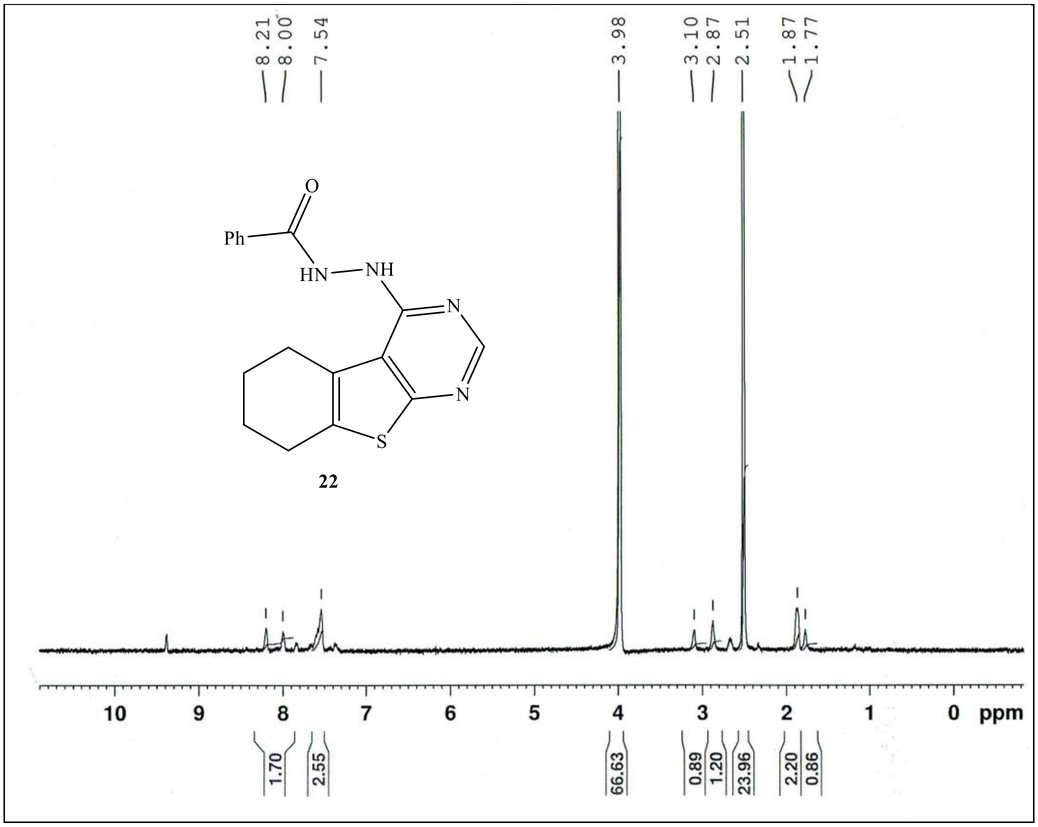

Fig. S54. ^{1}H NMR of **22** in DMSO-d_6 + D_2O

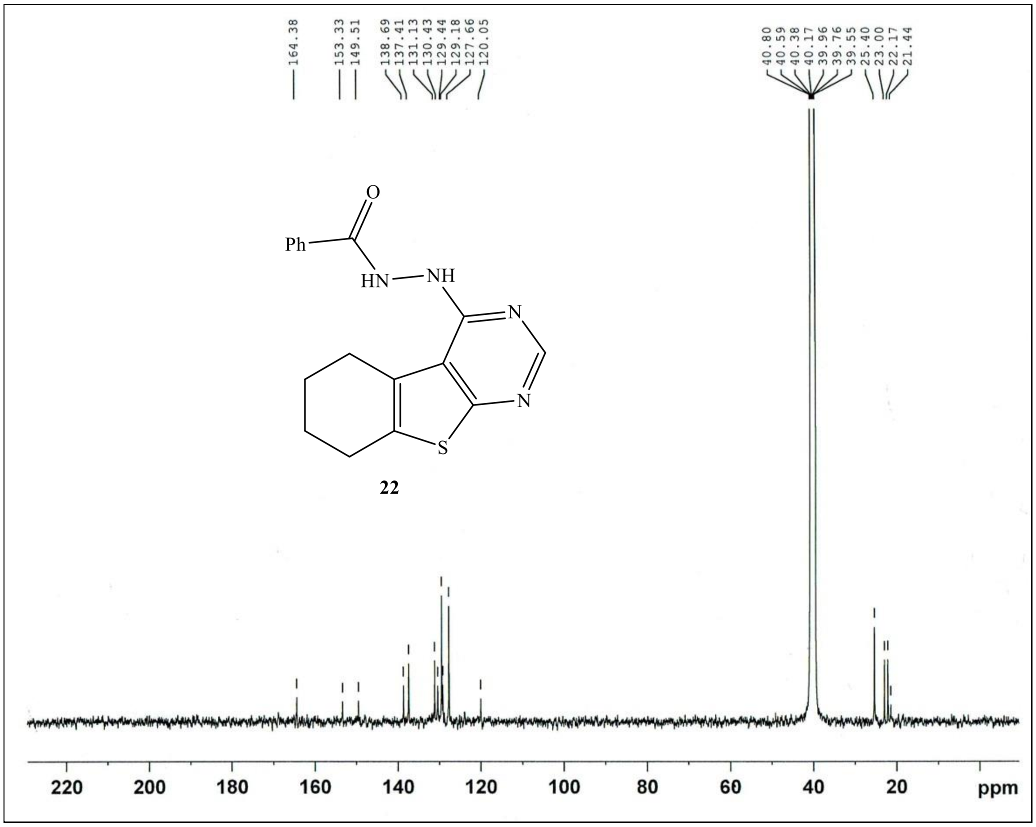

Fig. S55. ^{13}C NMR of **22** in DMSO-d_6

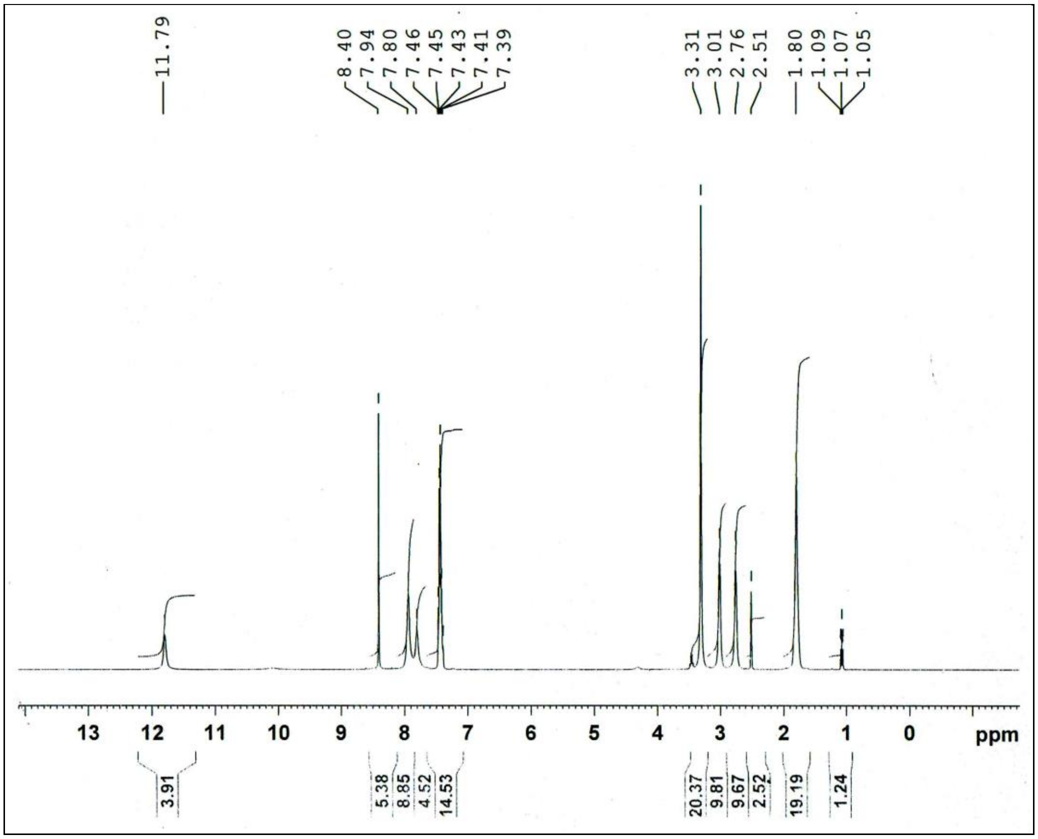

Fig. S56. ^{1}H NMR of **23** in DMSO-d_6

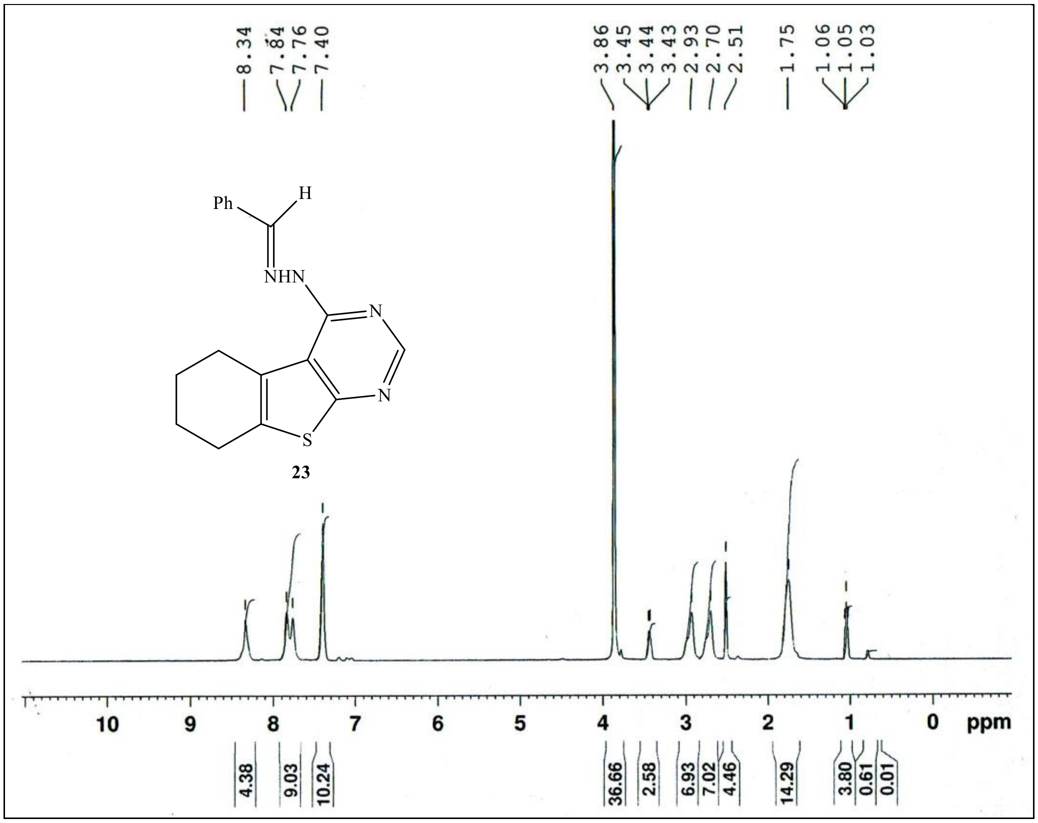

Fig. S57. ^{1}H NMR of **23** in DMSO-d_6 + D_2O

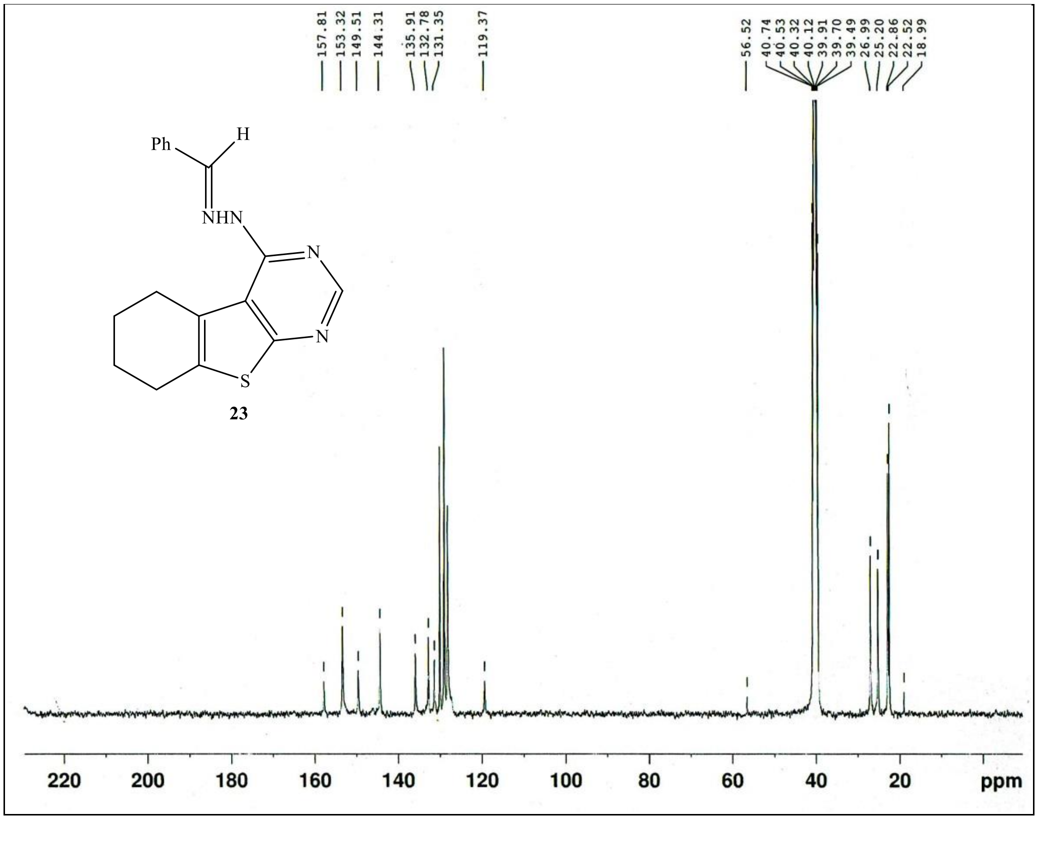

Fig. S58. ^{13}C NMR of **23** in DMSO-d_6

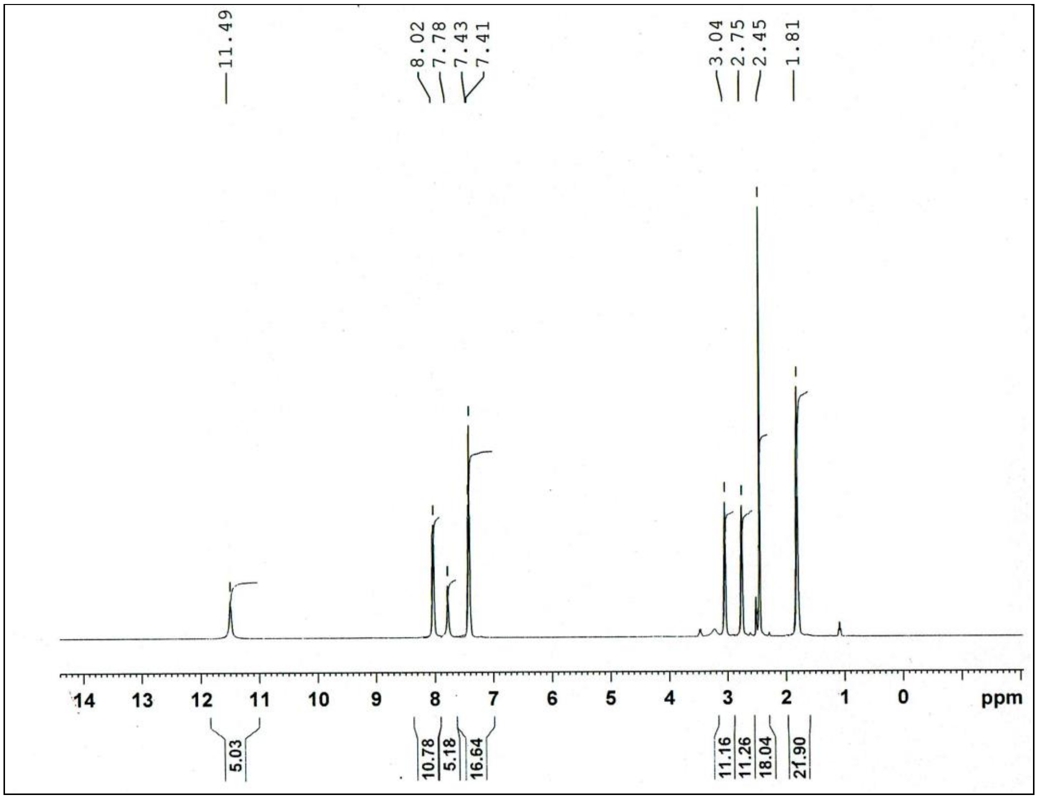

Fig. S59. ^{1}H NMR of **24** in DMSO-d_6

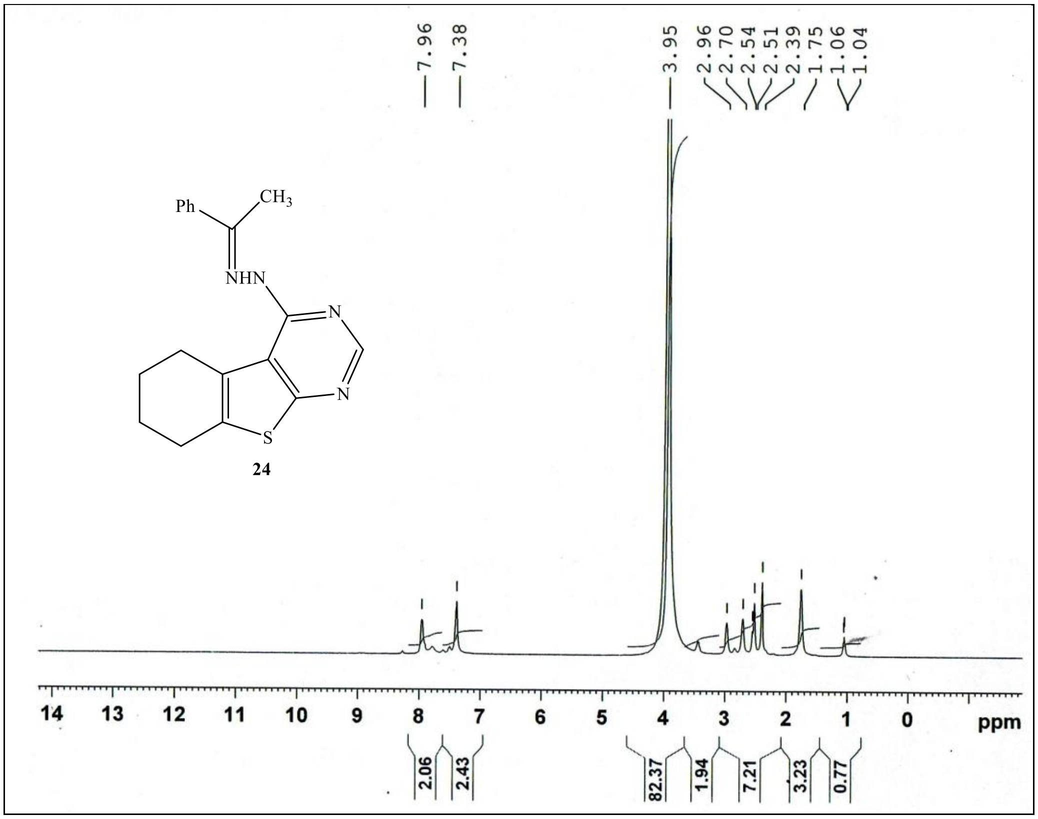

Fig. S60. ¹H NMR of **24** in DMSO-d_6 + D_2O

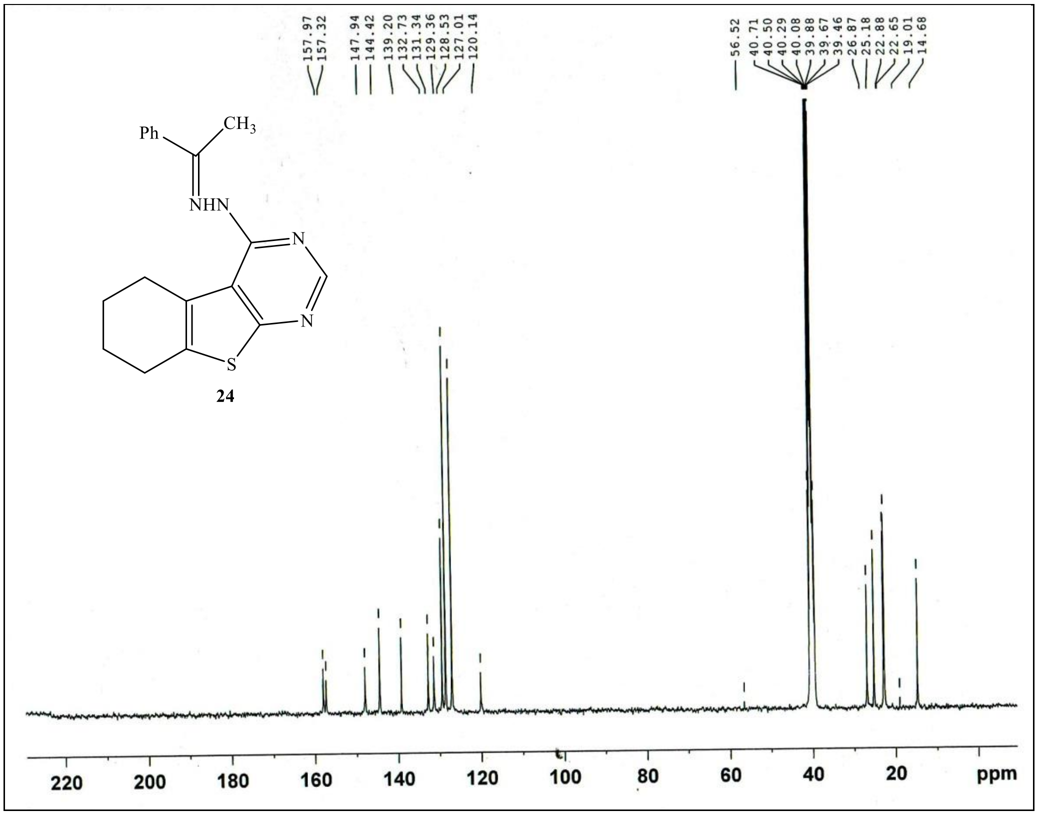

Fig. S61. ^{13}C NMR of **24** in DMSO-d_6

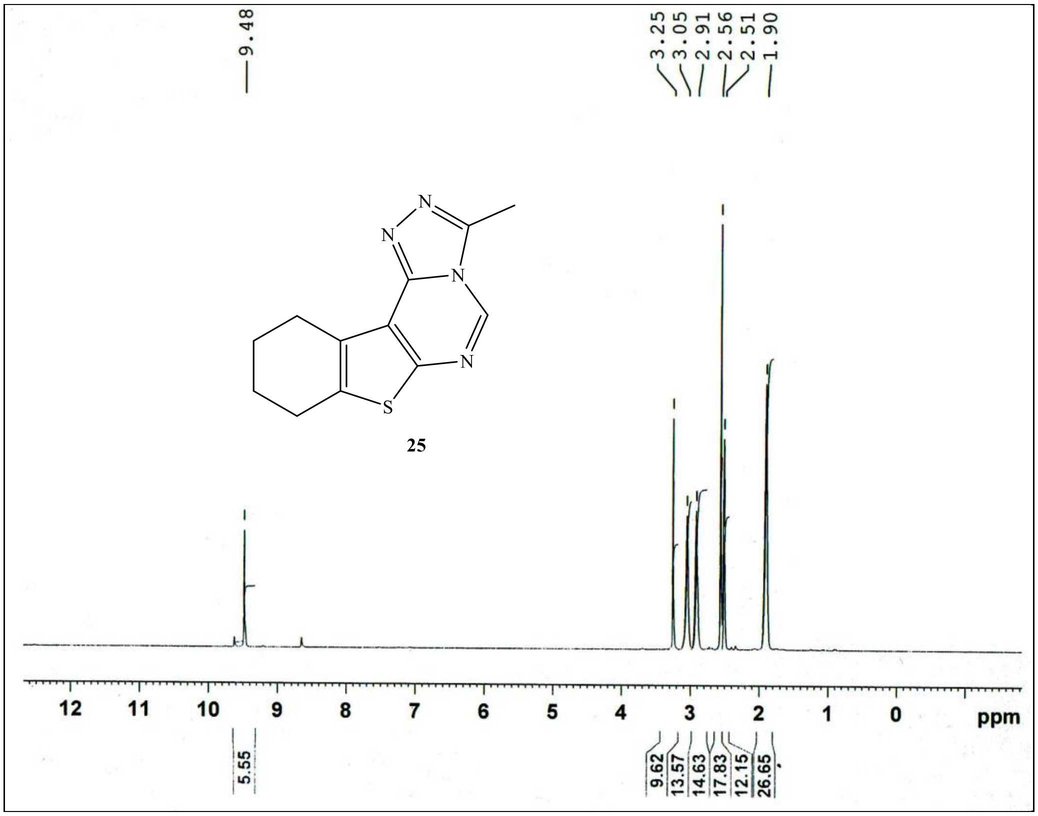

Fig. S62. ^{1}H NMR of **25** in DMSO-d_6

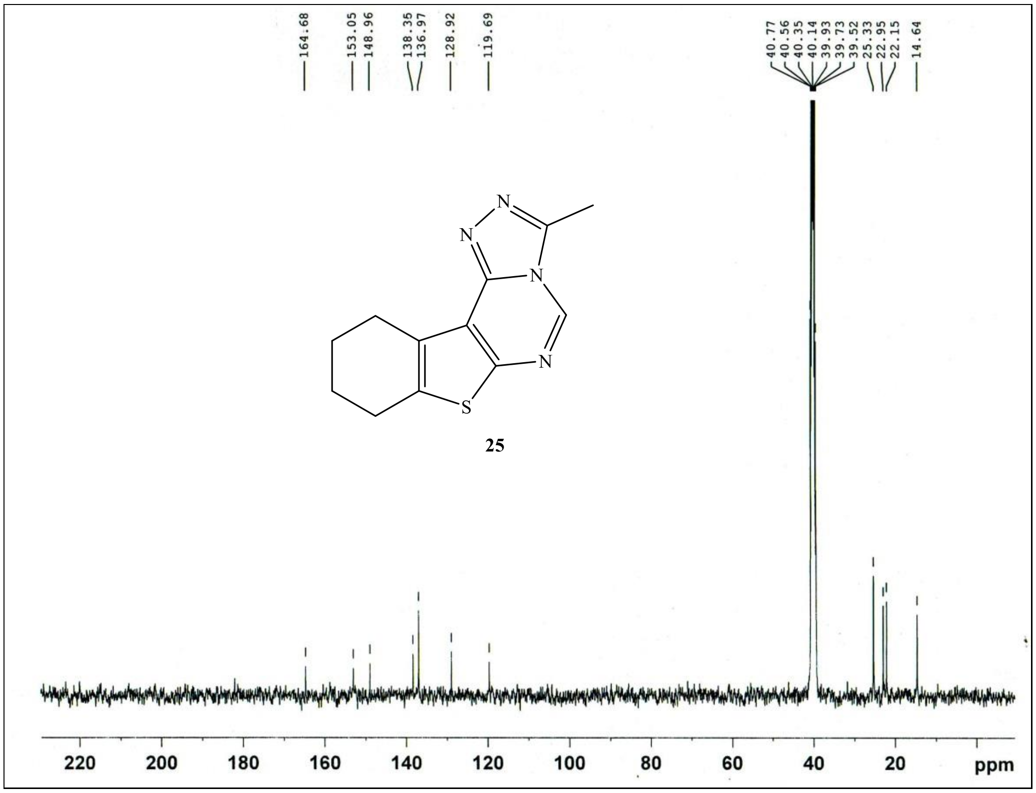

Fig. S63. ^{13}C NMR of **25** in DMSO-d_6

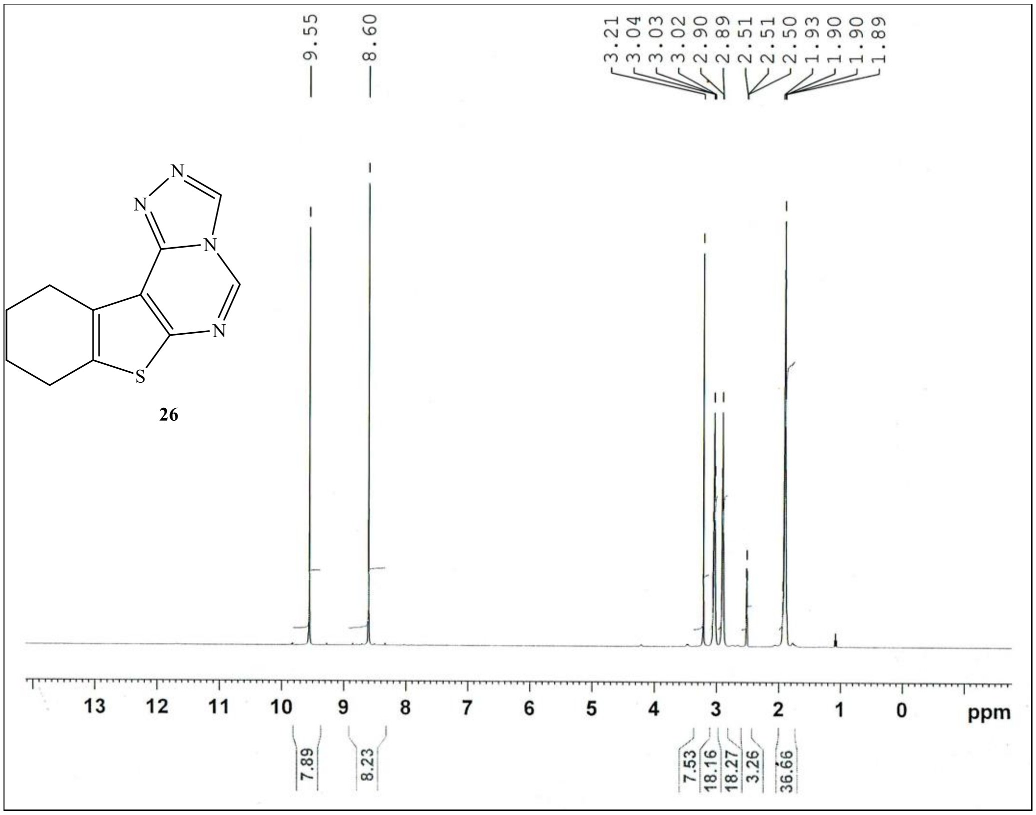

Fig. S64. ^{1}H NMR of **26** in DMSO-d_6

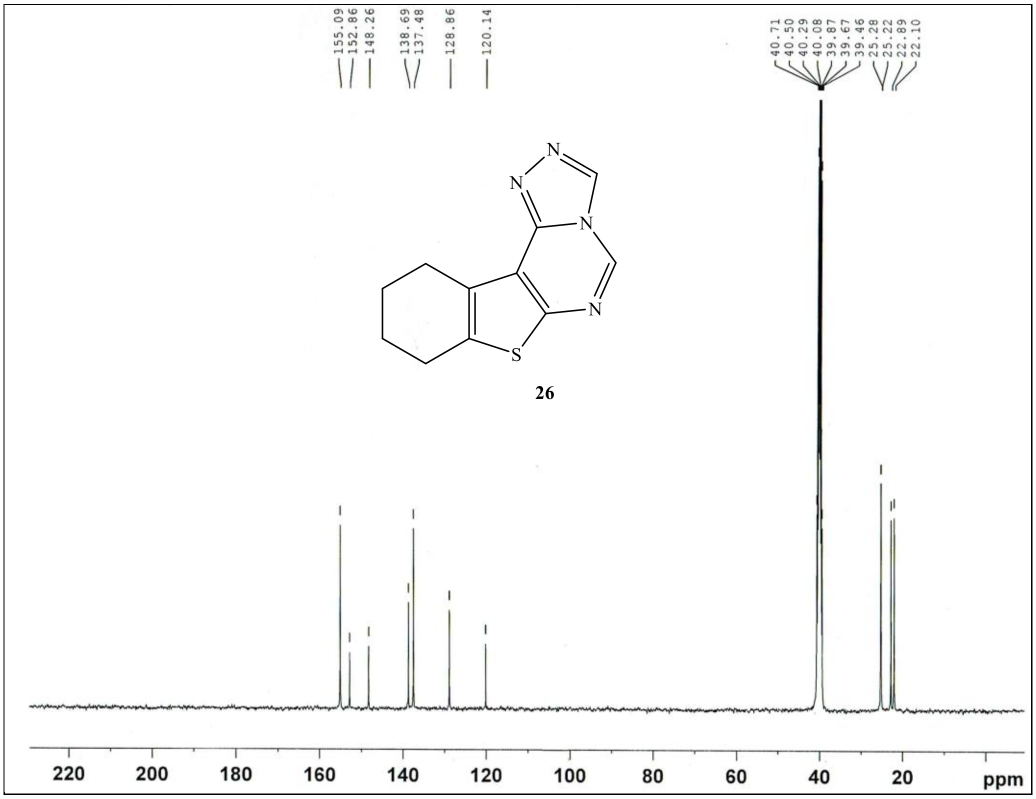

Fig. S65. ^{13}C NMR of **26** in DMSO-d_6

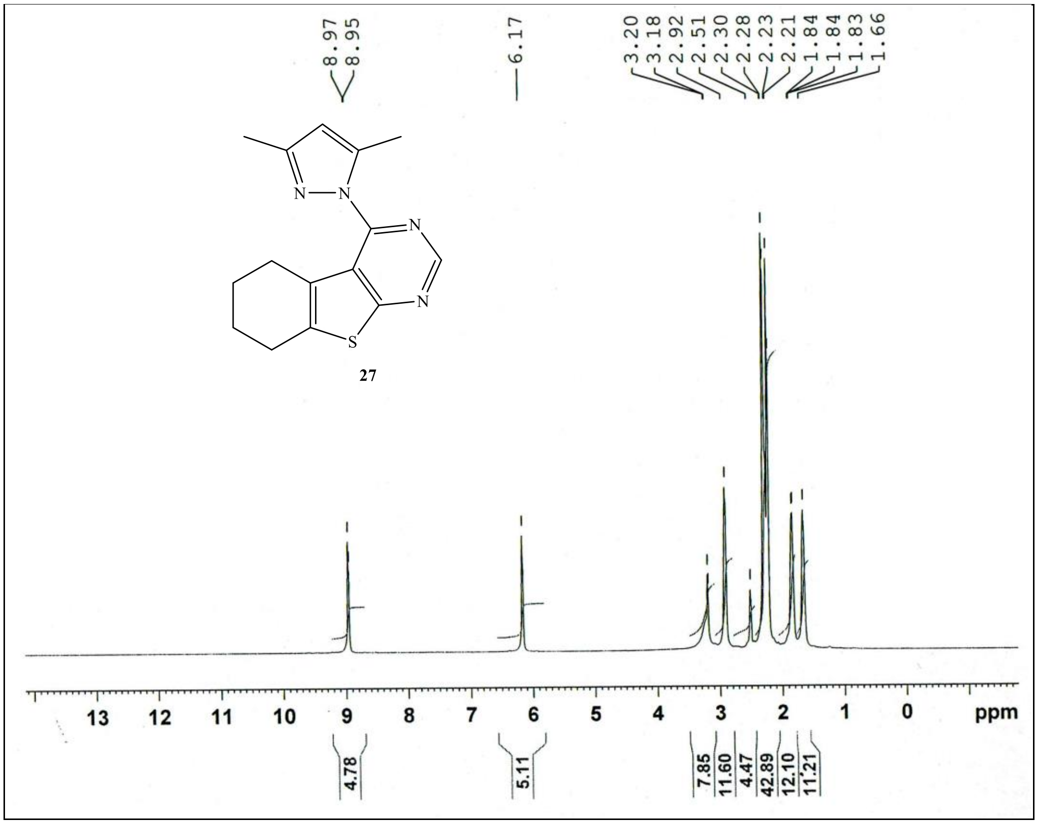

Fig. S66. ^{1}H NMR of **27** in DMSO-d_6

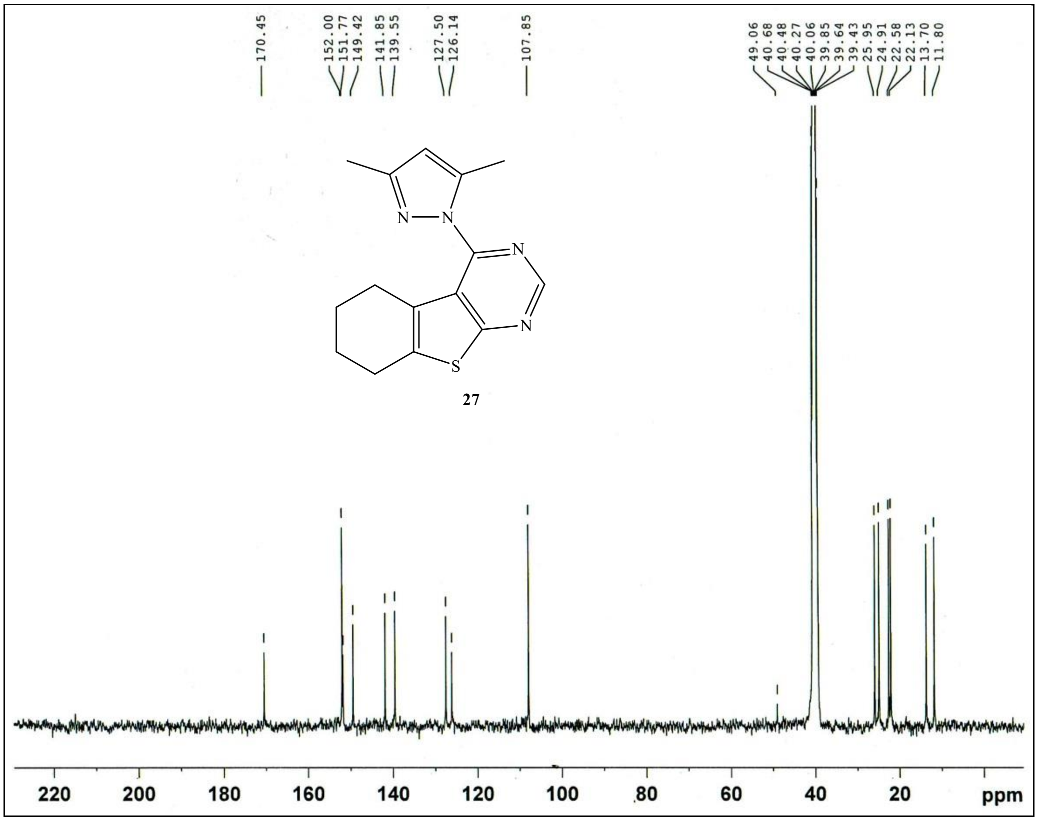

Fig. S67. ^{13}C NMR of **27** in DMSO-d_6

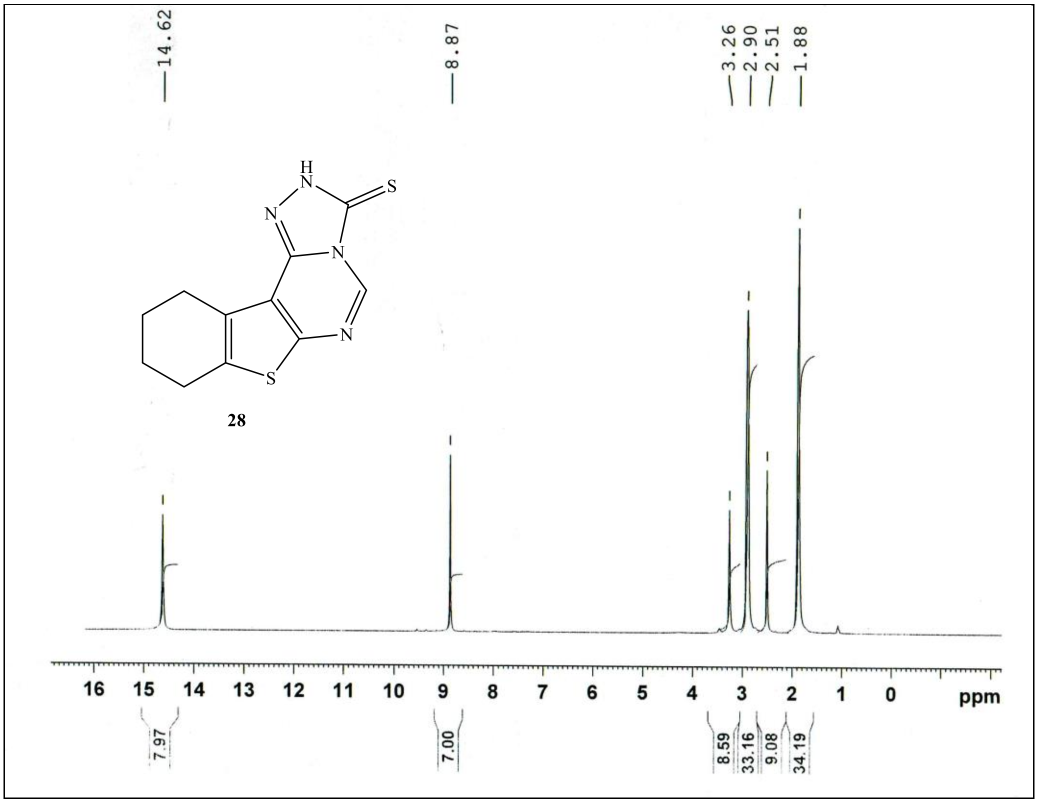

Fig. S68. ^{1}H NMR of **28** in DMSO-d_6

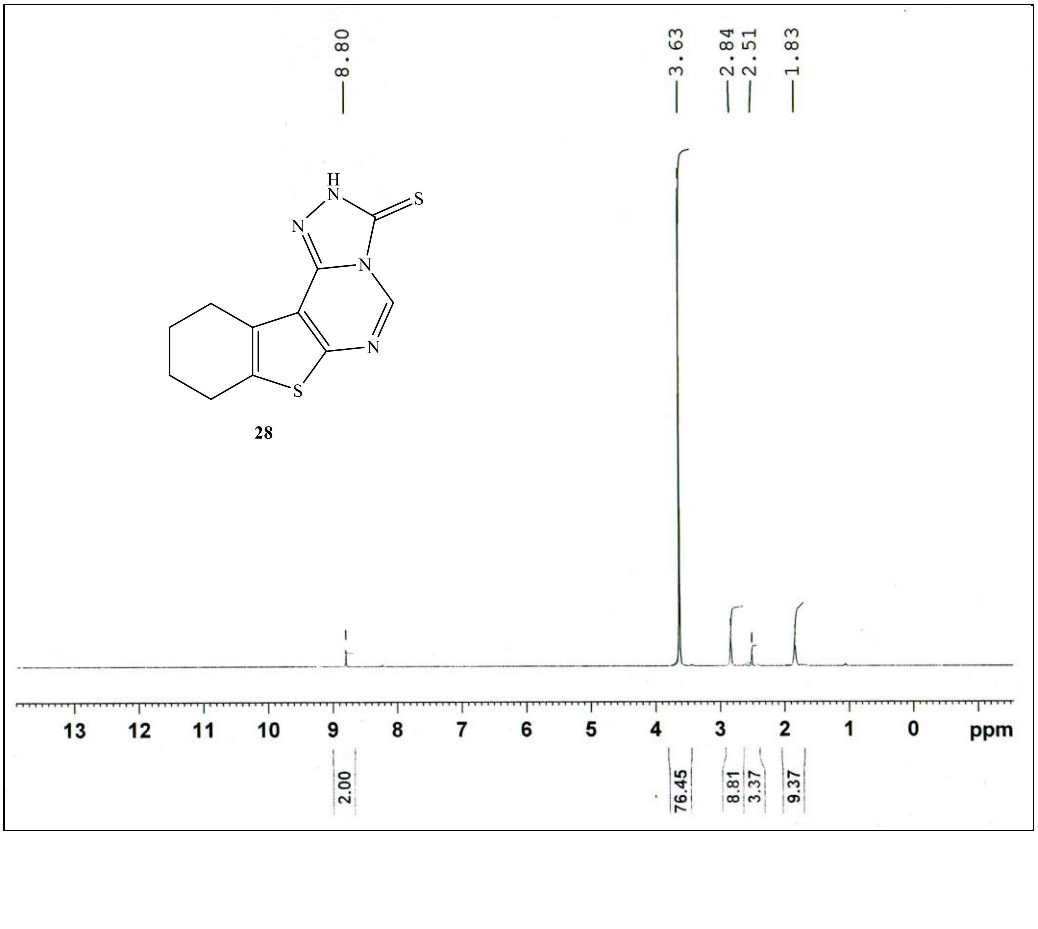

Fig. S69. ^{1}H NMR of **28** in DMSO-d_6 + D_2O

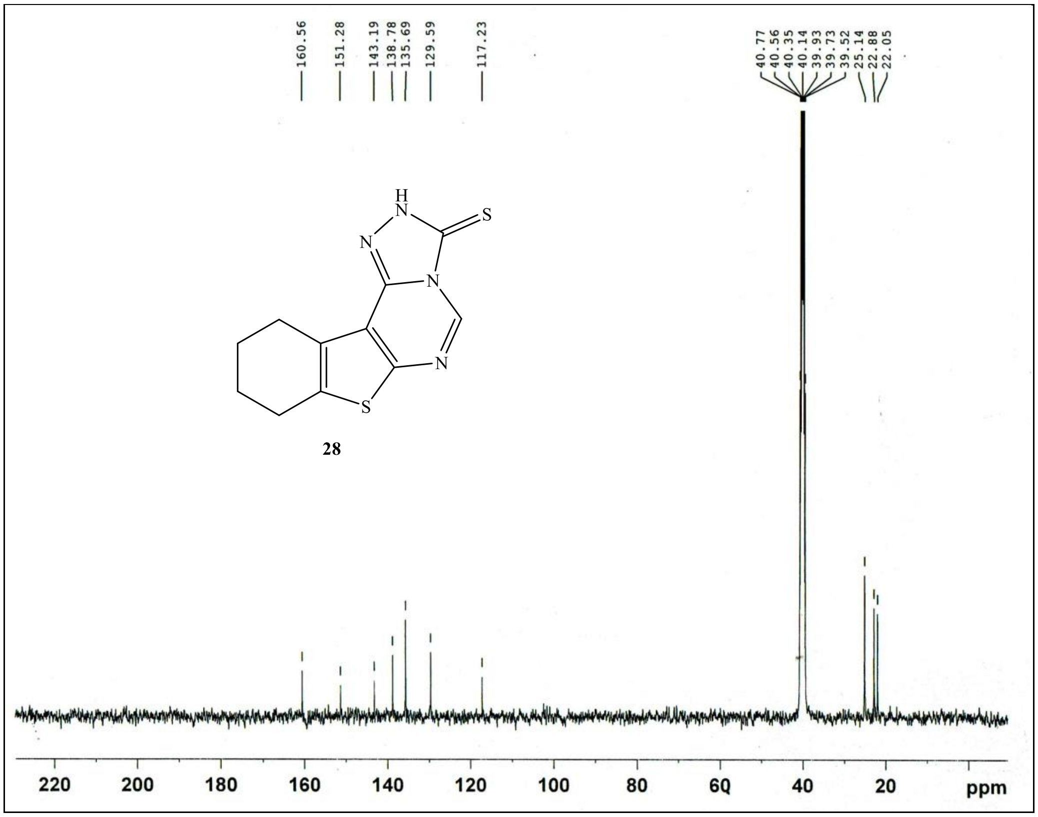

Fig. S70. ^{13}C NMR of **28** in DMSO-d_6

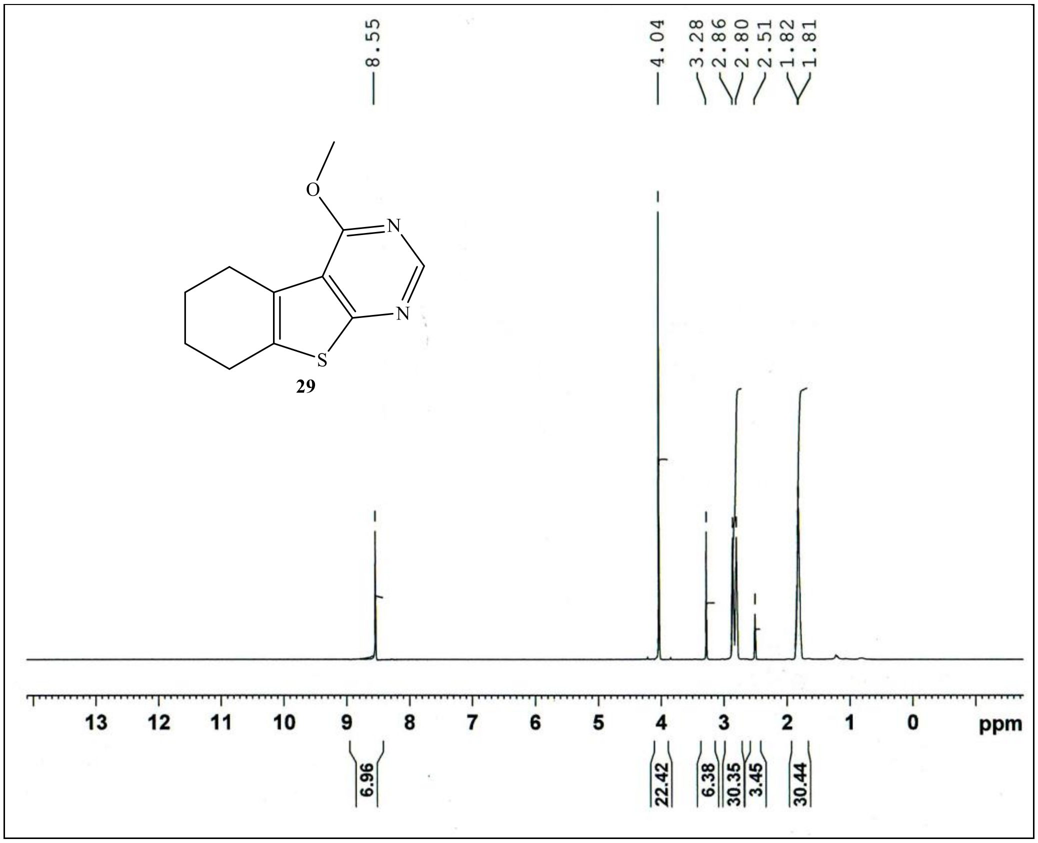

Fig. S71. ^{1}H NMR of **29** in DMSO-d_6

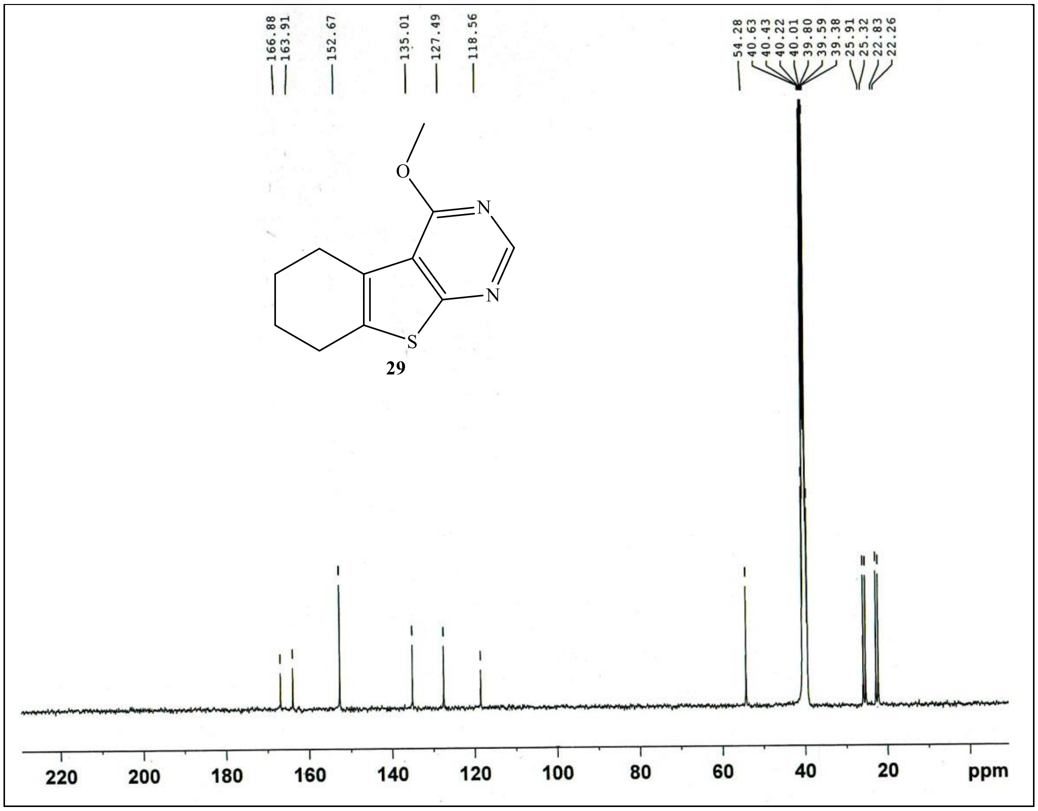

Fig. S72. ^{13}C NMR of **29** in DMSO-d_6

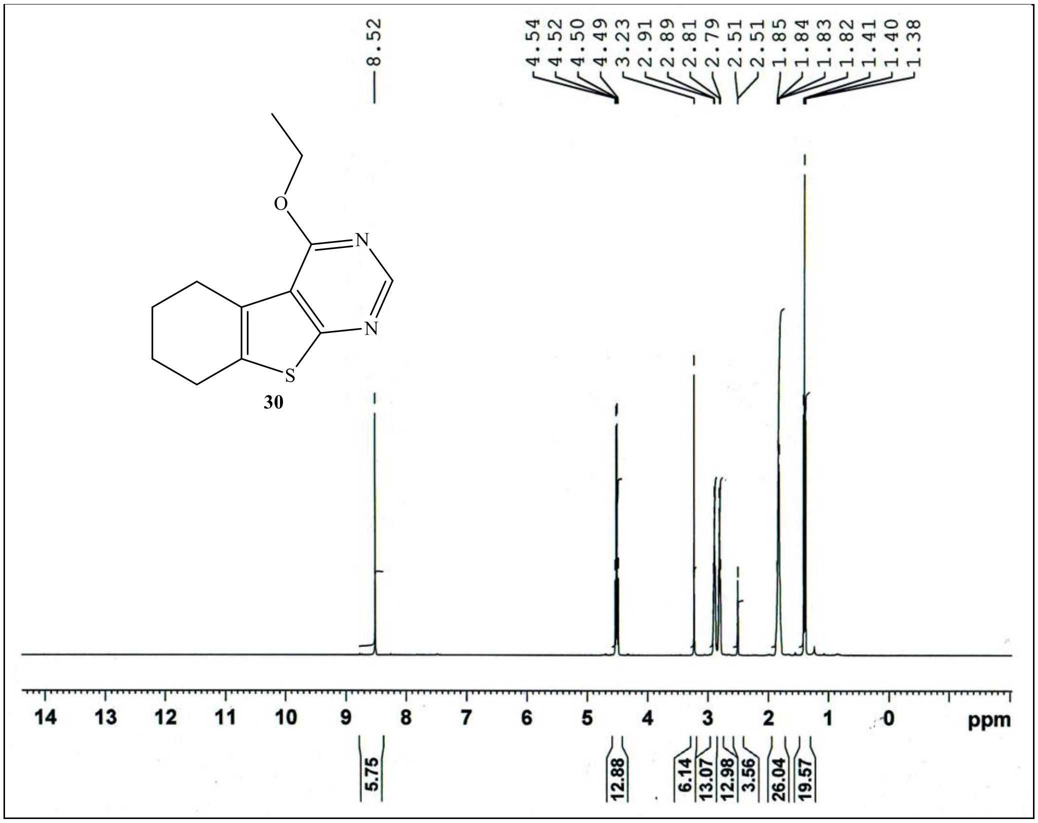

Fig. S73. ^{1}H NMR of **30** in DMSO-d_6

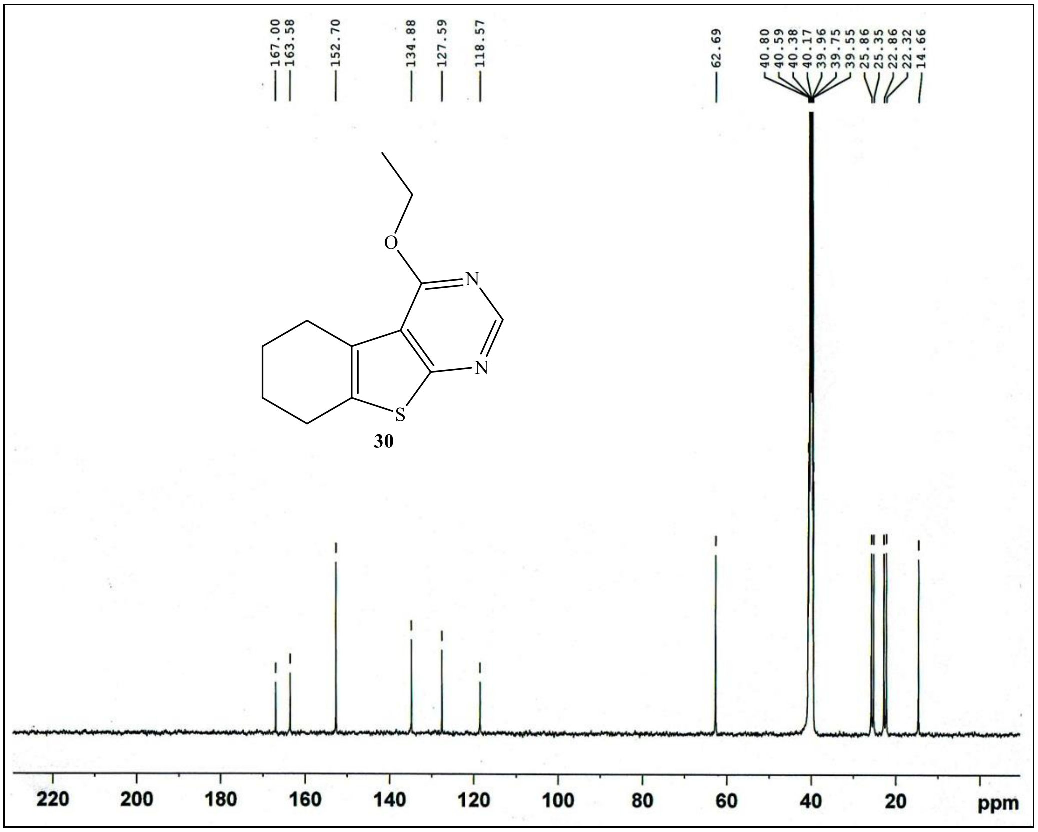

Fig. S74. ^{13}C NMR of **30** in DMSO-d_6

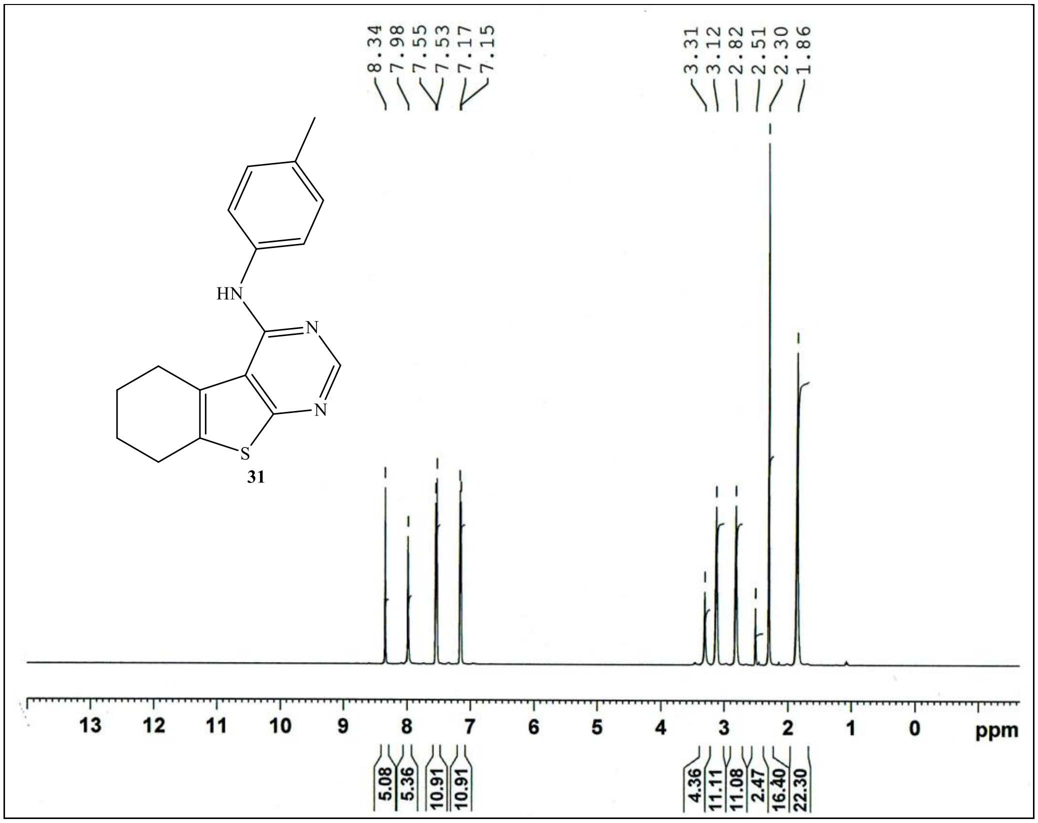

Fig. S75. ^{1}H NMR of **31** in DMSO-d_6

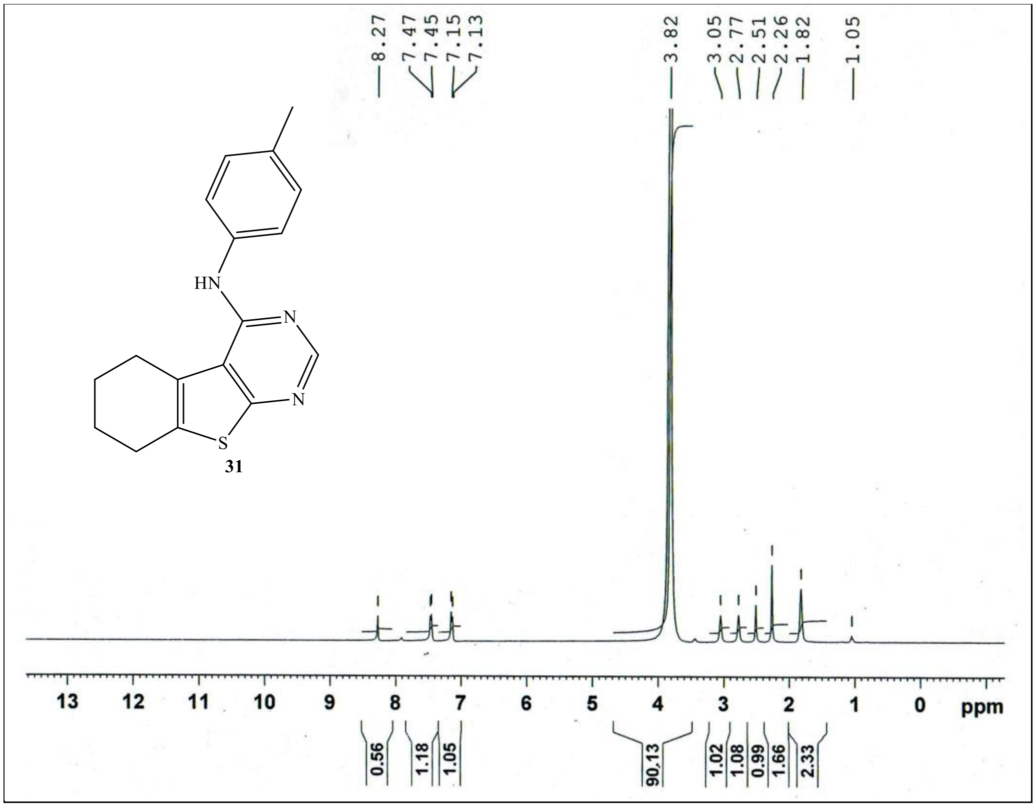

Fig. S76. ^{1}H NMR of **31** in DMSO-d_6 + D_2O

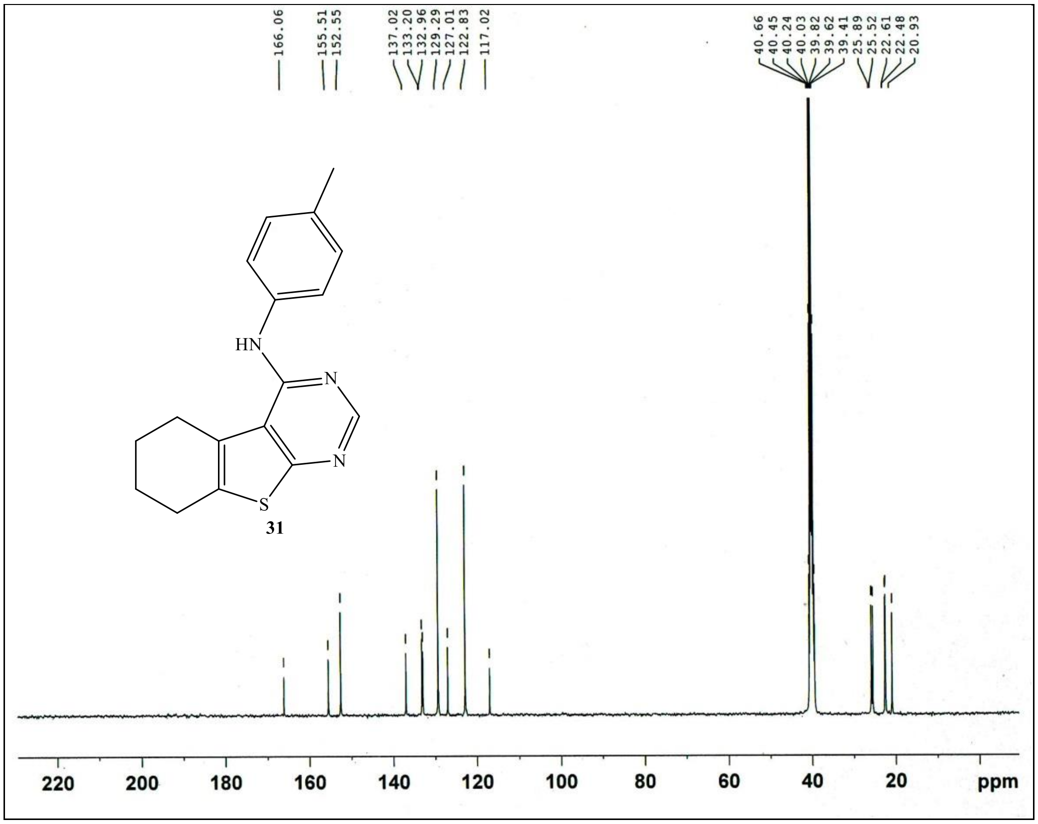

Fig. S77. ^{13}C NMR of **31** in DMSO-d_6

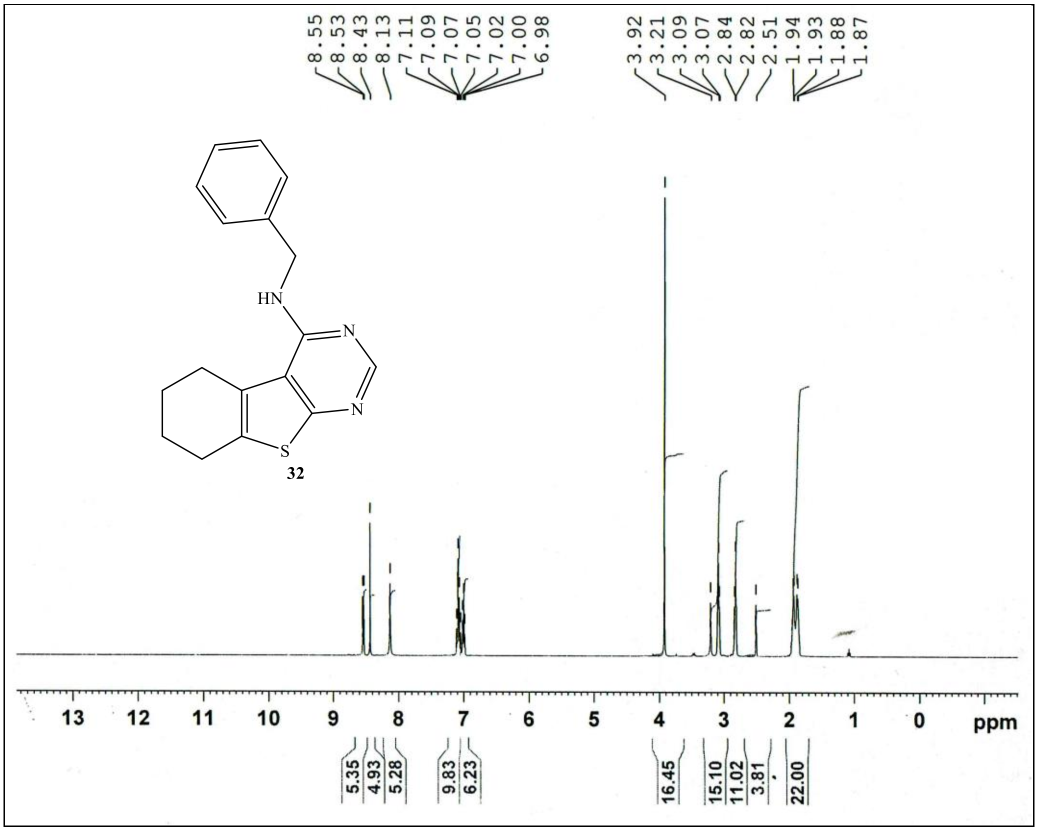

Fig. S78. ^{1}H NMR of **32** in DMSO-d_6

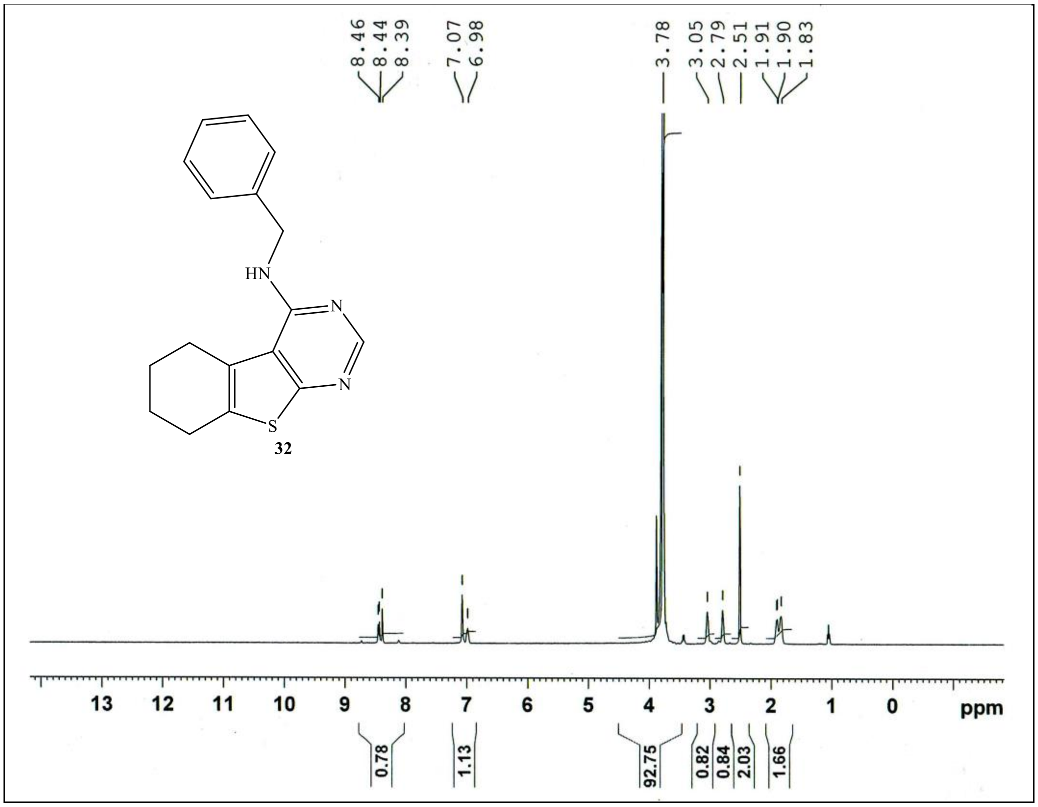

Fig. S79. ^{1}H NMR of **32** in DMSO-d_6 + D_2O

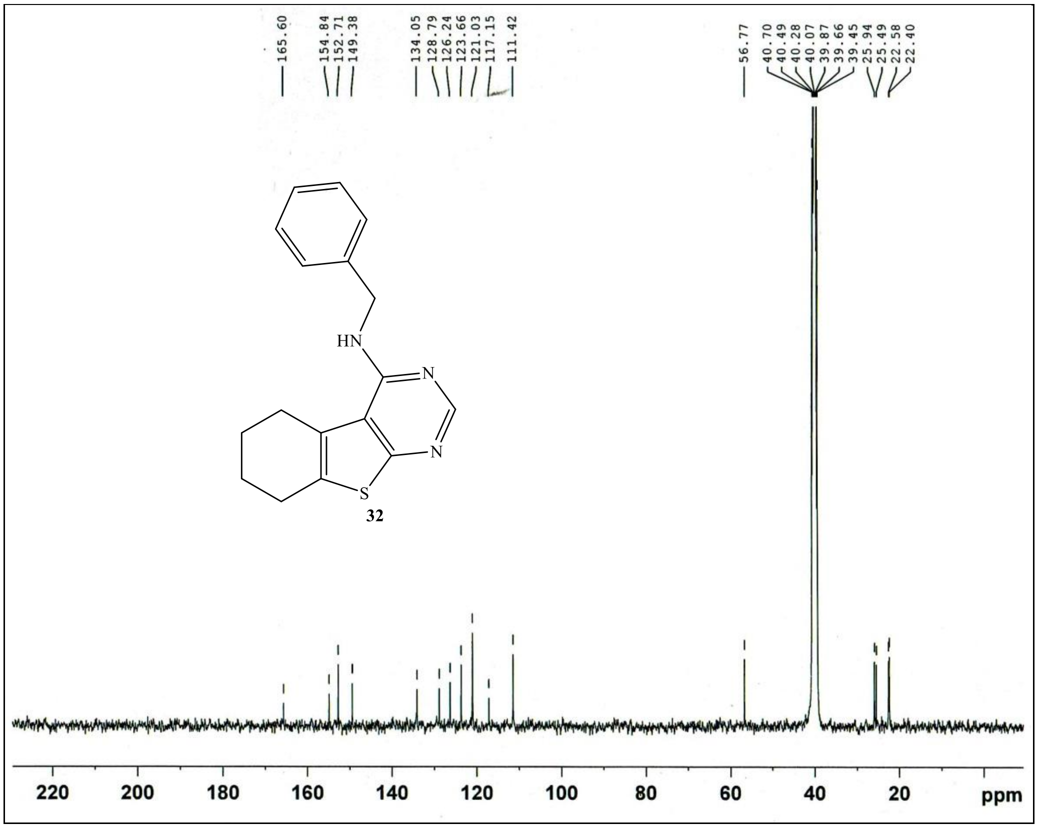

Fig. S80. ^{13}C NMR of **32** in DMSO-d_6

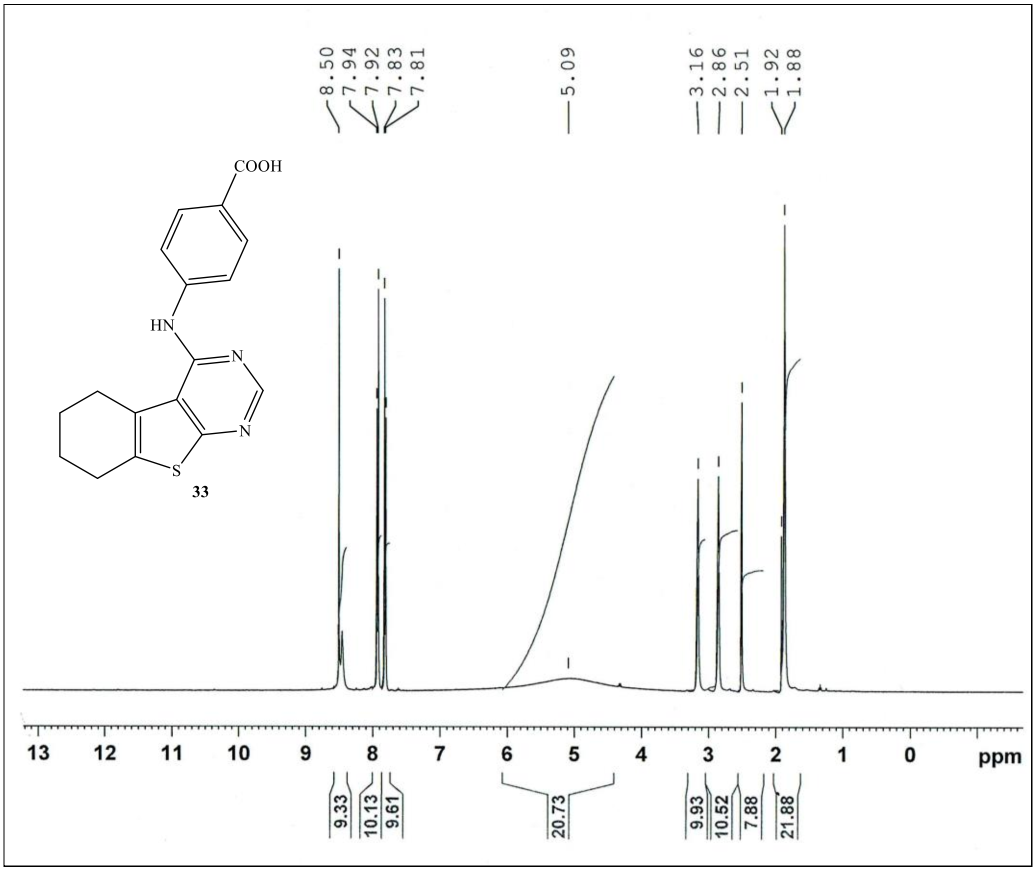

Fig. S81. ^{1}H NMR of **33** in DMSO-d_6

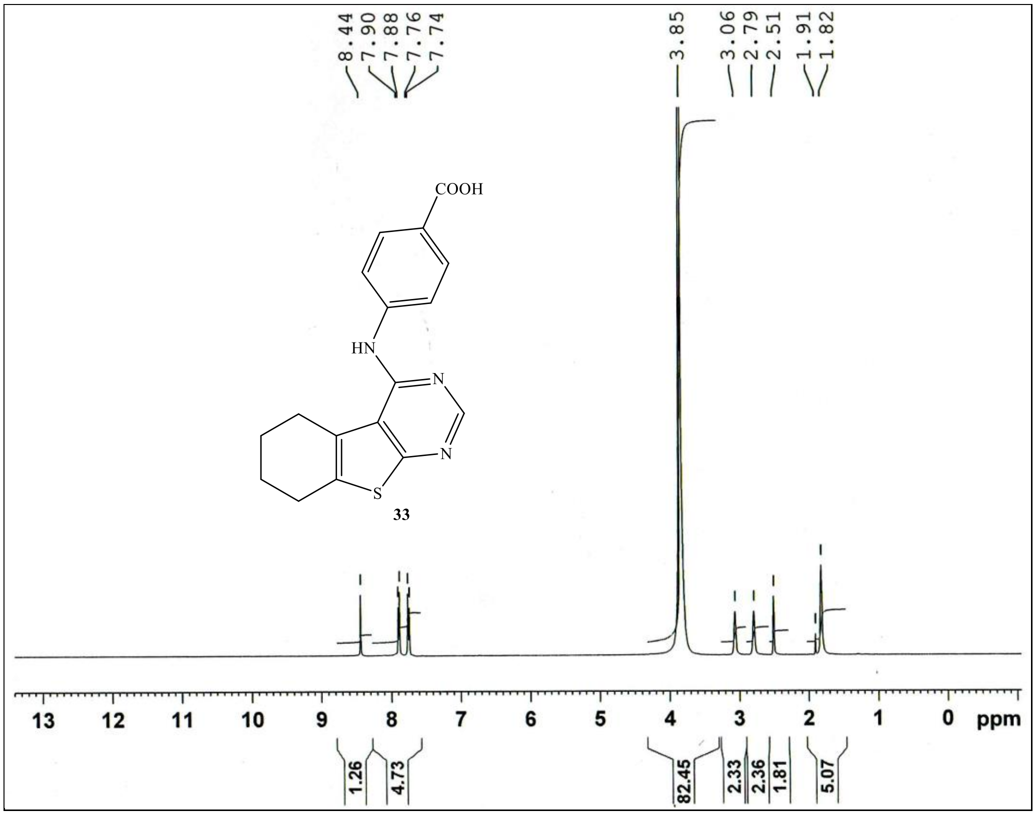

Fig. S82. ^{1}H NMR of **33** in DMSO-d_6 + D_2O

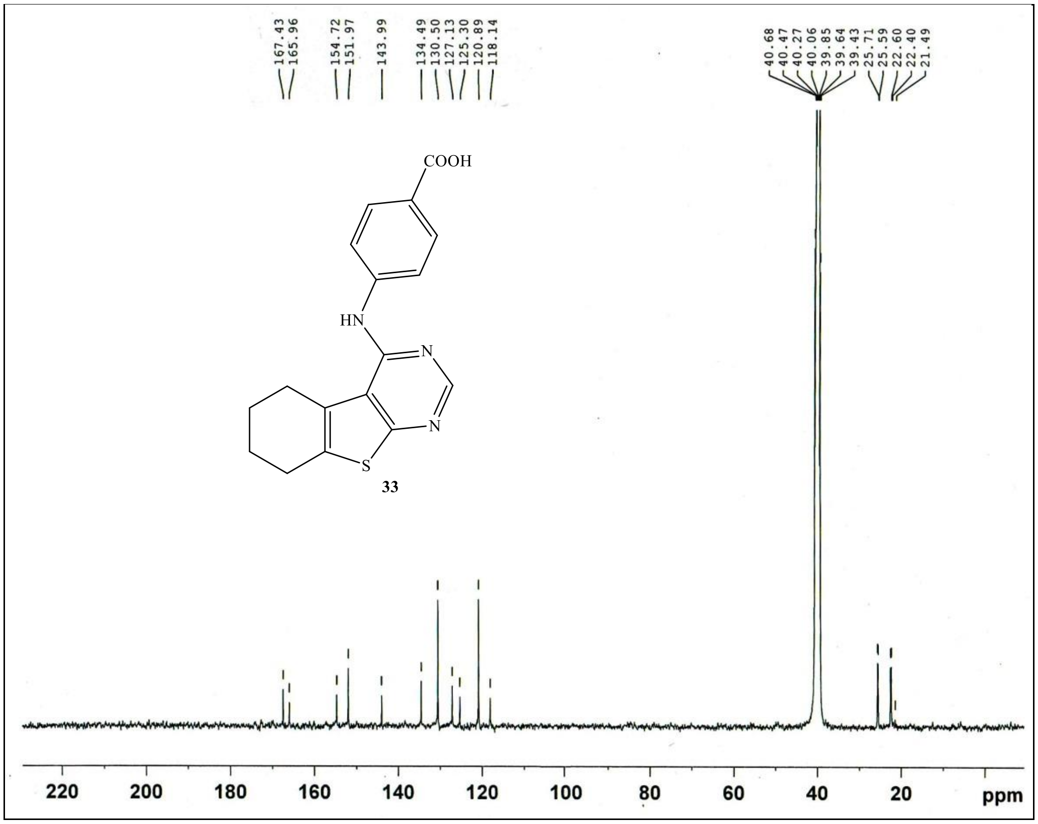

Fig. S83. ^{13}C NMR of **33** in DMSO-d_6

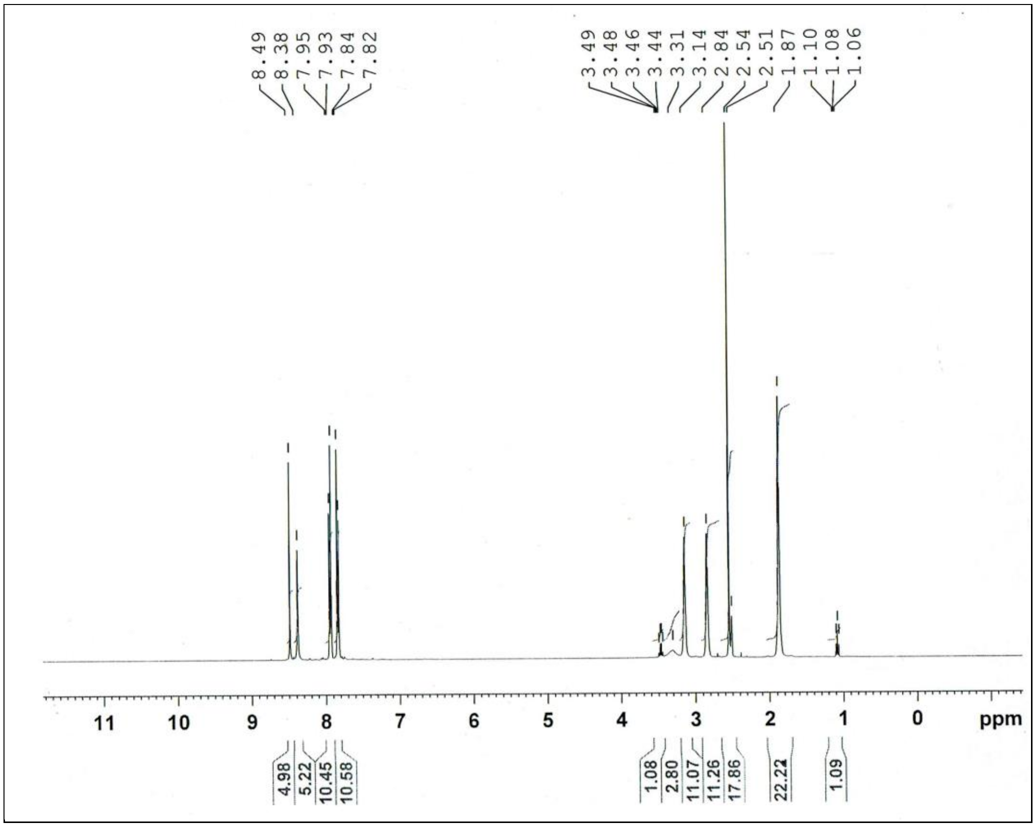

Fig. S84. ^{1}H NMR of **34** in DMSO-d_6

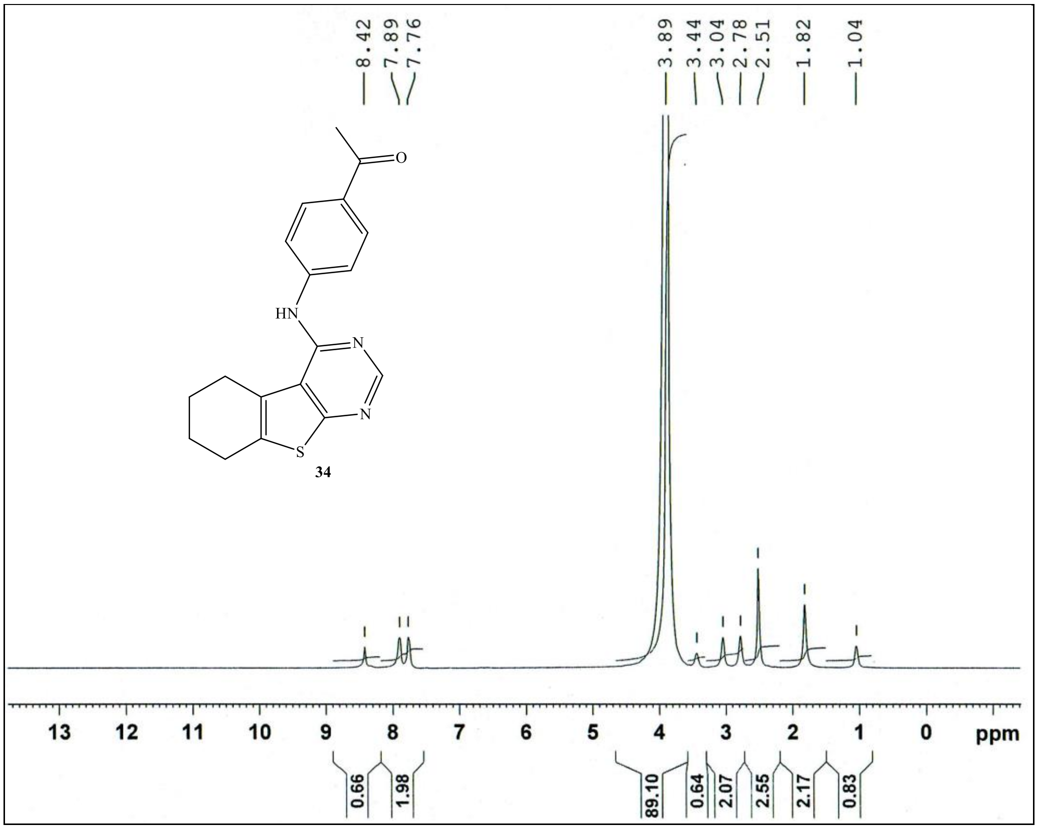

Fig. S85. ^{1}H NMR of **34** in DMSO-d_6 + D_2O

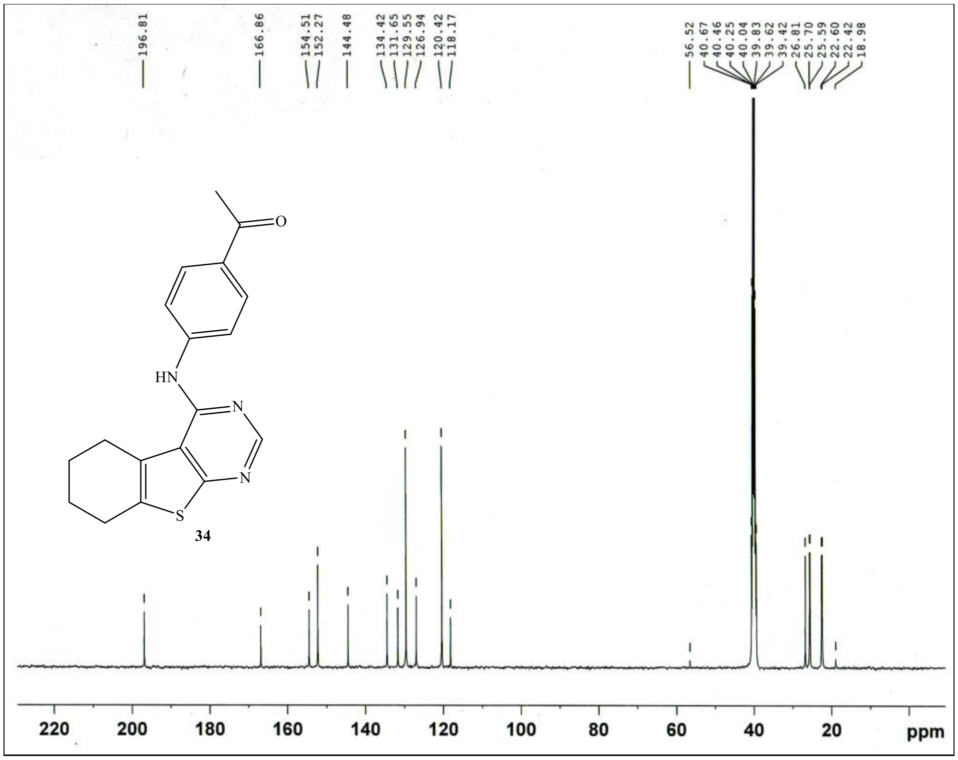

Fig. S86. ^{13}C NMR of **34** in DMSO-d_6

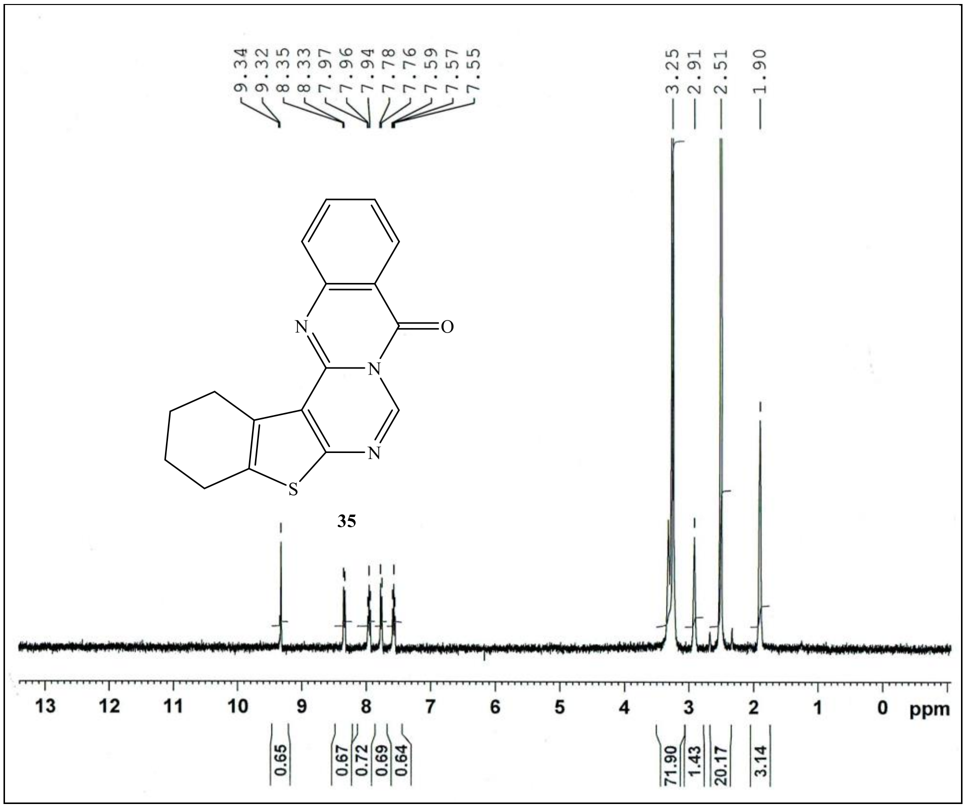

Fig. S87. ^{1}H NMR of **35** in DMSO-d_6
